# Loading of Dicarboxylatoplatinum(II)‐NHC Complexes in Bacterial Ghosts as an Advanced Development in Cancer Therapy

**DOI:** 10.1002/ardp.70108

**Published:** 2025-09-27

**Authors:** Amelie Scherfler, Klaus Wurst, Stefan Schwaiger, Francesco Baschieri, Martin Hermann, Daniel Baecker, Irena Pashkunova‐Martic, Brigitte Kircher, Hristo P. Varbanov

**Affiliations:** ^1^ Department of Pharmaceutical Chemistry, Institute of Pharmacy University of Innsbruck Innsbruck Austria; ^2^ Department of General, Inorganic, and Theoretical Chemistry University of Innsbruck Innsbruck Austria; ^3^ Department of Pharmacognosy, Institute of Pharmacy, Center for Molecular Biosciences Innsbruck University of Innsbruck Innsbruck Austria; ^4^ Institute of Pathophysiology Medical University of Innsbruck Innsbruck Austria; ^5^ Department of Anesthesiology & Critical Care Medicine Medical University of Innsbruck Innsbruck Austria; ^6^ Department of Pharmaceutical and Medicinal Chemistry, Institute of Pharmacy Freie Universität Berlin Berlin Germany; ^7^ Department of Biomedical Imaging and Image‐Guided Therapy, Division of Structural and Molecular Preclinical Imaging Medical University of Vienna and General Hospital of Vienna Vienna Austria; ^8^ Department of Internal Medicine V, Haematology & Oncology, Immunobiology and Stem Cell Laboratory Medical University of Innsbruck Innsbruck Austria; ^9^ Tyrolean Cancer Research Institute Innsbruck Austria

**Keywords:** bacterial ghosts, cytotoxicity, NHC ligands, ovarian cancer, platinum(II) complexes

## Abstract

This study aimed to improve the drug‐like properties of benzimidazole‐based Pt(II)‐N‐heterocyclic carbene (NHC) complexes, particularly by enhancing their water solubility and delivery to cancer cells. Accordingly, four new Pt(II) complexes of the benzimidazol‐2‐ylidene type, featuring monodentate carboxylato ligands, were prepared and their structures confirmed through a combination of spectroscopic and crystallographic techniques. Their stability in aqueous solution and cell culture medium was investigated by ^1^H NMR spectroscopy and HPLC‐MS analysis. Cytotoxicity was assessed using the MTT assay in ovarian cancer cell lines (A2780wt (cisplatin sensitive) and A2780cis (cisplatin resistant)) and a noncancerous bone marrow stromal cell line (HS‐5). Most complexes exhibited cytotoxicity comparable to or exceeding that of carboplatin, with preferential activity toward cancer cells. Loading of all four Pt(II) complexes into bacterial ghost cells (BGs) derived from two different nonpathogenic bacterial strains, *Escherichia coli (E. coli)* Nissle 1917 and *E. coli* NM522 notably enhanced the intracellular accumulation and cytotoxicity. Furthermore, mechanistic studies demonstrated that all tested compounds, regardless of formulation, induced apoptosis. Their potential to trigger immunogenic cell death was also evaluated, though only a modest effect was observed on selected hallmarks. Collectively, these findings highlight the potential of dicarboxylatoplatinum(II)‐NHC complexes, particularly loaded into BG‐based formulations, as promising anticancer drug candidates.

## Graphical abstract

To enhance the drug‐like properties of benzimidazole‐based Pt(II)‐NHC complexes, derivatives with carboxylato leaving groups were developed, improving solubility without compromising potency against ovarian cancer cells. Loading in bacterial ghosts derived from *Escherichia coli* further increased cellular accumulation and cytotoxic activity, highlighting a promising formulation strategy for platinum‐based therapeutics.

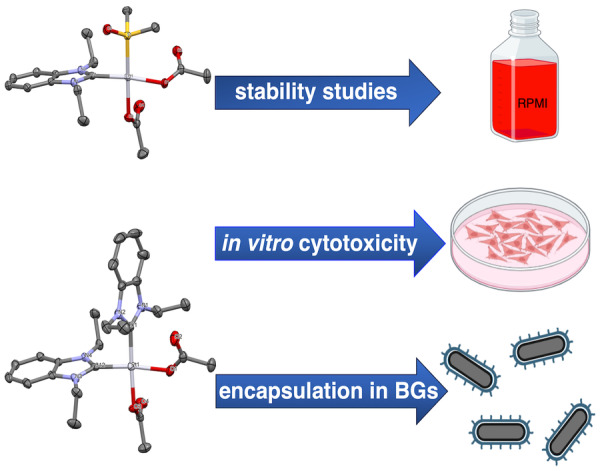

**Abbreviations**
AAS: atomic absorption spectrometry
ACN: acetonitrile
ATP: adenosine triphosphate
BG: bacterial ghost
CBDC: 1,1'‐cyclobutane dicarboxylate
DAMP: damage‐associated molecular pattern
DCA: dichloroacetate
DCM: dichloromethane
DMF: dimethylformamide
DMSO: dimethyl sulfoxide
DNA: deoxyribonucleic acid
*EcN*
: *Escherichia coli* Nissle 1917
*E. coli*
: *Escherichia coli*
*Ec* NM522: *Escherichia coli* NM522
ESI‐HRMS: electrospray ionization–high resolution mass spectrometry
FACS: fluorescence‐activated cell sorting
FCS: fetal calf serum
GF: graphite furnace
HR CS AAS: high resolution continuous source atomic absorption spectrometry
HSP: heat shock protein
Hz: Hertz
ICD: immunogenic cell death
ICP‐MS: inductively coupled plasma mass spectrometry
MeOH: methanol
MTT: 3‐(4,5‐dimethylthiazol‐2‐yl)‐2,5‐diphenyltetrazolium bromide
NHC: N‐heterocyclic carbene
NMR: nuclear magnetic resonance
OD: optical density
PBS: phosphate buffered saline
PI: propidium iodide
ppm: parts per million
RPMI: Rosewell Park Memorial Institute
RP‐HPLC‐MS: reverse‐phase high‐performance liquid chromatography mass spectrometry
rt: room temperature
SD: standard deviation
SEM: standard error of mean
TEM: transmission electron microscopy

## 1 Introduction

Despite being one of the most extensively studied groups of diseases, cancer remains a leading cause of mortality worldwide [1]. Current treatment options include chemotherapy, radiation therapy, surgical removal of the tumor, gene therapy, hormone therapy, or immunotherapy [2]. Concerning chemotherapy, platinum‐based drugs are among the most widely used therapeutics [3, 4]. Their most prominent representative is cisplatin ((*SP*‐4‐2)‐diamminedichloridoplatinum(II)), whose antitumor activity in vivo was first reported by Rosenberg et al. in 1969 [5]. Further developments in platinum‐based chemotherapeutics have led to the introduction of carboplatin ((*SP*‐4‐2)‐diammine(1,1‐cyclobutanedicarboxylato)platinum(II)) [6] and oxaliplatin ((*SP*‐4‐2)‐(1*R*,2*R*‐diaminocyclohexane)oxalatoplatinum(II)) [7]. Although platinum drugs are highly active against a variety of cancers, their clinical effectiveness is limited by a wide spectrum of side effects, as well as both primary and acquired resistances [8, 9].

Platinum complexes with N‐heterocyclic carbene (NHC) ligands represent a promising class of compounds, offering potential advantages over current platinum‐based chemotherapeutics, by possibly providing alternative mechanisms of action to circumvent drug resistance [10, 11, 12, 13]. Most reported compounds in this class feature imidazole‐ or benzimidazole‐based NHC ligands and halogenido leaving groups, while they may also include ancillary ligands such as dimethyl sulfoxide (DMSO), phosphines, amines, amino acids, N‐heterocycles, or other carbenes [14, 15, 16]. Despite their potential, the advancement of these complexes in preclinical development is often limited by poor water solubility and premature inactivation before reaching target cancer cells [17]. One strategy to address these challenges, previously applied to other platinum complexes to optimize solubility, lipophilicity, and reactivity, involves modifying the leaving groups (e.g., replacing halides with carboxylates) [18, 19, 20]. Alternatively, incorporating these complexes into advanced drug delivery systems, such as biological transport vehicles and nanoparticles, may enhance their stability and therapeutic efficacy [21].

Platinum‐based drugs are commonly known for their interactions with deoxyribonucleic acid (DNA), which play a key role in their mechanism of action. These interactions result in the formation of platinum–DNA adducts, causing DNA damage that ultimately triggers apoptosis—a well‐established mode of action for platinum(II) complexes [22, 23, 24]. In 2010, Tesniere et al. investigated the activation of the immune system as a potential mode of action for oxaliplatin [25]. In particular, immunogenic cell death (ICD) was induced in colon cancer cells via treatment with oxaliplatin. ICD is characterized by a change in the cell surface of the dying cell and the release of damage‐associated molecular patterns (DAMPs), for example, calreticulin, high‐mobility‐group‐protein B1, or certain heat‐shock proteins (HSP), namely HSP70 and HSP90. These DAMPs elicit an immune response by interacting with antigen presenting cells, such as dendritic cells, which attract other immune cells like T‐cells [25]. Additionally, other platinum complexes, including those featuring NHC ligands, have also been reported to induce ICD [26, 27].

Bacterial ghosts (BGs) are an innovative carrier system that uses biologically grown structures. BGs are Gram‐negative bacteria cell envelopes formed by the controlled expression of the bacteriophage PhiX174's lysis gene E by expelling the cytoplasm and all cell organelles [28]. These cell envelopes can be filled with a variety of biologically active substances such as peptides, foreign DNA, or drugs [29], and thus serve as advanced drug delivery systems. Furthermore, it has been demonstrated that systemic administration of attenuated bacteria induces a robust Th1‐dominated immune response in the tumor area through polarized T‐cells [30] ultimately leading to ICD [31]. Groza et al. proposed an experimental treatment of colorectal cancer that combines oxaliplatin administration with probiotic BGs to enhance the immune response and drug efficacy, potentially allowing lower dosages and improved therapeutic outcome [29]. Despite the obtained promising results for coadministration of oxaliplatin and BGs, as well as the potential of BGs as a biological carrier system, no reports have described the loading of platinum complexes in BGs so far.

In this study, we report the development of four new platinum(II)‐NHC complexes of the benzimidazol‐2‐ylidene type, featuring monodentate carboxylato (acetato or dichloroacetato, DCA) ligands. Acetate was chosen to enhance aqueous solubility [18]. DCA, on the other hand, is an inhibitor of pyruvate dehydrogenase kinase that can trigger mitochondrial apoptosis in cancer cells and it is a commonly used ligand in the development of dual action platinum(II)‐ and platinum(IV)‐based anticancer agents [32, 33, 34]. The synthesized complexes were thoroughly characterized by electrospray ionization‐high resolution mass spectrometry (ESI‐HRMS), nuclear magnetic resonance (NMR) spectroscopy, elemental analysis, and X‐ray crystallography. Their stability in common organic solvents, water, and cell culture medium was assessed by ^1^H NMR spectroscopy and high‐performance liquid chromatography mass spectrometry (HPLC‐MS). To enhance stability and potentially enable targeted delivery to cancer cells, the compounds were loaded into two strains of BGs *E. coli* Nissle 1917 (*EcN*) and *E. coli* NM522 (*Ec* NM522). Cytotoxicity of the compounds and their cellular accumulation, both in free and loaded forms, were evaluated in ovarian carcinoma cells A2780wt and A2780cis (a cisplatin‐resistant subvariant). Furthermore, their ability to induce intracellular DNA damage, apoptosis, and ICD was investigated to gain insights into their mechanism of action.

## 2 Results and Discussion

### 2.1 Synthesis and Characterization

**Figure 1:**
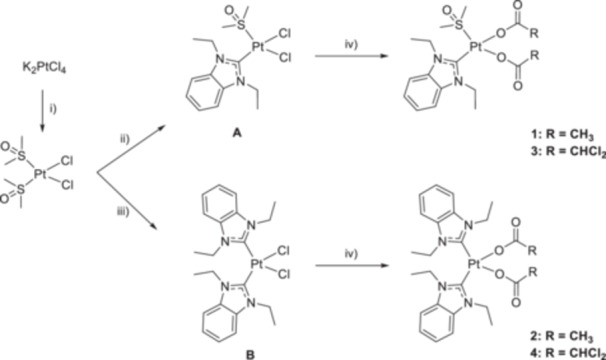
Synthesis of dicarboxylatoplatinum(II)‐NHC complexes **1**–**4**. (i) DMSO, H_2_O, room temperature (rt); (ii) 1,3‐diethylbenzimidazol‐2‐ium hexafluorophosphate, NaHCO_3_, DMSO, 80°C; (iii) 1,3‐diethylbenzimidazol‐2‐ium chloride, NaOMe, acetonitrile (ACN), 35°C; (iv) CH_3_COOAg or CHCl_2_COOAg, DCM.

*N*,*N′*‐Diethylbenzimidazol‐2‐ylidene platinum(II) complexes featuring monodentate carboxylato ligands (**1**–**4**) were synthesized from their dichlorido derivatives (**A**–**B**) via reaction with the respective silver carboxylate salt in dichloromethane (DCM) (Figure 1) with yields greater than 80%. The complexes were characterized in detail by multinuclear NMR spectroscopy (see Supporting Information S2: Figures S1–S5), ESI‐HRMS (see Supporting Information S2: Figures S6–S9), and X‐ray crystallography. Their purity (> 95%) was verified by elemental analysis.

The ^1^H NMR resonances of the coordinated NHC moiety in the new complexes were found within a similar range to those of their dichlorido counterparts, exhibiting comparable spectral patterns. In bis(NHC) complexes **2** and **4**, the N‐CH₂ protons were chemically and magnetically nonequivalent, appearing as two distinct multiplets, consistent with previous observations for *cis*‐configurated complexes of the benzimidazolylidene type (Supporting Information S2: Figures S2 and S4) [17, 35]. A comparable splitting was also observed for compounds **1** and **3**, which contain one NHC and one DMSO ligand. However, in these cases, the multiplets had closer chemical shifts and partially overlapped (Supporting Information S2: Figures S1 and S3). In contrast, the N‐CH₂ methylene protons in the dichlorido analog **A** appeared as a quartet. The ¹H NMR signals of the coordinated carboxylato ligands appeared as singlets at 1.94 parts per million (ppm) for the acetate protons in complex **2** and at 5.91 ppm for the dichloroacetate protons in complex **4**. In complexes **1** and **3**, the two carboxylato ligands are nonequivalent, resulting in a double set of signals in both ^1^H and ^13^C NMR spectra (Supporting Information S2: Figures S1 and S3). This splitting arises from their asymmetrical environments, with one ligand *trans* to DMSO and the other to the NHC ligand.

Replacing the chlorido ligands in **A** and **B** with acetato (**1**, **2**) or DCA (**3**, **4**) ligands caused a downfield shift in the ^195^Pt resonance by 182–192 ppm (**A** vs. **1**, **3**) or 267–294 ppm (**B** vs. **2**, **4**). In contrast, the nature of the carboxylato ligand (acetato vs. DCA) had a lesser impact on the ^195^Pt chemical shift, likely due to the distance of the substituents from the ^195^Pt nucleus (see Supporting Information S2: Figure S5).

ESI‐HRMS spectra also confirmed the identity of the new complexes. In positive ion mode, the peak assigned to [M‐RCOO^−^]^+^ displayed the highest intensity. Additional peaks corresponding to the loss of a second carboxylate and dimeric species, such as [2M‐RCOO^−^]^+^, were also observed in most cases. The *m*/*z* values and the isotopic distribution were in accordance with calculated data. Finally, the structures of the complexes were unambiguously confirmed by X‐ray crystallography (see below).

### 2.2 Crystal Structures

**Figure 2:**
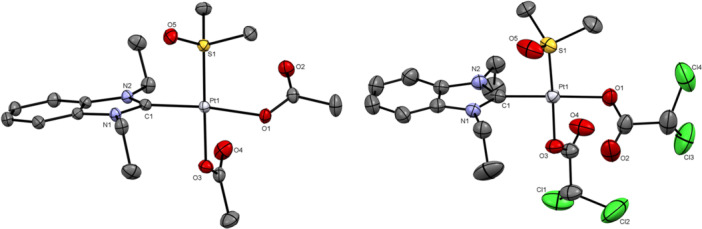
Molecular structures of complexes **1** (left) and **3** (right). The thermal ellipsoids have been drawn at the 50% probability level. Hydrogen atoms are omitted for clarity.

**Figure 3:**
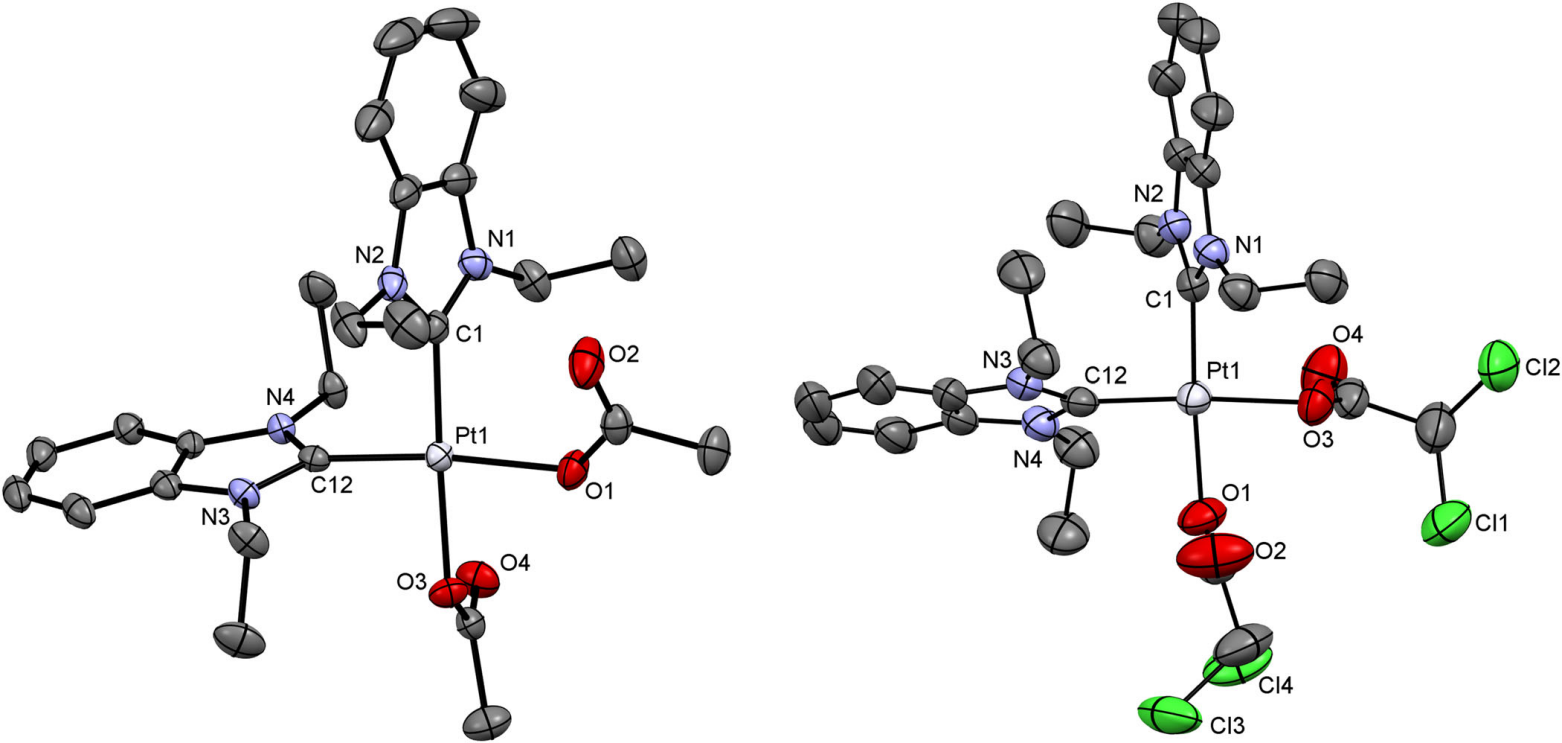
Molecular structures of complexes **2** (left) and **4** (right). The thermal ellipsoids have been drawn at the 50% probability level. Hydrogen atoms and solvent molecules are omitted for clarity.

The molecular structures of complexes **1**–**4** were determined by single crystal X‐ray diffraction analysis (see Figures 2 and 3). Suitable single crystals were obtained by slow evaporation of chloroform or methanol (MeOH)/DCM solutions of the respective compound. Selected bond lengths and angles are listed in Table 1. Crystal data and structure refinement details are provided in the Supporting Information (Supporting Information S2: Tables S1–S4).

**Table 1:** Selected bond lengths and angles of complexes **1–4**.

complex	1	2	3	4
Bond length (Å)				
Pt‐C1	1.979	1.966	1.973	1.964
Pt‐C12	—	1.968	—	1.959
Pt‐S1	2.199	—	2.192	—
Pt‐O1	2.053	2.068	2.056	2.074
Pt‐O3	2.041	2.070	2.038	2.089
Angle (°)				
C‐Pt‐C(S)	91.97	92.33	90.15	91.91
O‐Pt‐O	83.65	82.46	89.97	84.63

Complexes **1** and **3** crystallized in the monoclinic space groups *P*2_1/n_ and *P*2_1/c_, respectively. Complexes **2** and **4** crystallized in the triclinic space group *P*‐1, each co‐crystallizing with chloroform molecules—in a 1:2 ratio for complex **2** and a 1:1 ratio for complex **4**. All platinum(II) complexes exhibited square planar geometry, with C–Pt–C angles slightly exceeding 90° and O–Pt–O angles close to 85° (both angles are nearly 90° in complex **3**). The platinum‐carbene carbon (Pt‐C1, Pt‐C12) bond lengths ranged from 1.959 to 1.979 Å, consistent with those observed in dichlorido analogs **A** (1.983 Å) and **B** (1.964 Å) [16, 17]. The Pt–S and Pt–O bond lengths were comparable to those reported for related complexes [16, 18, 33].

### 2.3 Stability Studies

The dicarboxylatoplatinum(II)‐NHC complexes are well soluble in common organic solvents, such as MeOH, ACN, DMSO, dimethylformamide (DMF), DCM, and chloroform. As anticipated, substituting both chlorido ligands with acetato ligands significantly enhanced water solubility, increasing from < 0.5 mg/mL for complexes **A** and **B** to approximately 10 mg/mL for complex **1** and around 3 mg/mL for complex **2**. In contrast, the complexes bearing DCA ligands (**3** and **4**) exhibited only limited water solubility (< 0.5 mg/mL).

Complexes **1**–**4** were found to be stable in pure chloroform, ACN, and DMF over a time span of 24 h at rt, as evidenced by ^1^H NMR spectroscopy (exemplarily shown in Supporting Information S2: Figures S10–S11) and RP‐HPLC experiments (Supporting Information S2: Figures S18–S19). In contrast, dissolution in MeOH led to the formation of additional species, likely due to partial exchange of one or two carboxylato ligands with MeOH and/or trace amounts of water present in the solvent. The amount of these species, accounting for 15%–25% relative to the parent complex, remained largely unchanged over 24 h of incubation at rt (exemplarily shown in Supporting Information S2: Figures S12, S13, S20).

Classical platinum(II) complexes (such as cisplatin, carboplatin, and oxaliplatin) bind to their cellular target, the nuclear DNA, only after their leaving groups (chlorido or carboxylato ligands) are displaced by water, while the carrier am(m)ine ligands remain coordinated to the platinum center to enable effective DNA binding [3]. Aminediacetatoplatinum(II) complexes undergo substitution reactions in aqueous media similar to those of cisplatin [18, 36]. To better understand the behavior of dicarboxylatopaltinum(II)‐NHC complexes under biologically relevant conditions, the fate of water‐soluble complexes **1** and **2** in aqueous solution and cell culture medium was investigated by ^1^H NMR spectroscopy and HPLC‐MS.

¹H NMR spectroscopy measurements in D₂O revealed rapid aquation of both complexes. For complex **1**, a prominent signal corresponding to free (uncoordinated) acetate appeared within 5 min of dissolution, indicating that over 70% of the complex had already released its acetates. No significant spectral changes were observed during incubation for 24 h at rt, suggesting the formation of a stable aquated species (see Supporting Information S2: Figures S14–S15). Following dissolution in D_2_O, the ^1^H NMR spectrum of complex **2** showed three distinct signals for the acetato protons, likely corresponding to the native diacetato complex, a monoaquated intermediate, and free acetate (see Supporting Information S2: Figures S16–S17). The intensity of the free acetate signal gradually increased over time, accompanied by a slight downfield shift, possibly due to a slow pH change in the solution. After 6 h of incubation, minor additional signals emerged, suggesting the formation of new species in small amounts over prolonged incubation. These observations suggest a fast initial aquation of complex **2**, possibly followed by an equilibrium between mono‐ and di‐aquated species.

Monitoring the reactivity of complexes **1** and **2** by reversed phase (RP)‐HPLC‐MS proved challenging due to broad, tailing signals. Furthermore, the use of common mobile phase additives such as formic acid had to be avoided, as acetate‐to‐formate exchange during elution would further complicate the analysis. A freshly prepared aqueous solution of complex **2** showed a broad main peak at *t*_R_ = 11.2 min, corresponding to a species with *m*/*z* 602.2. This signal progressively decreased over time. This observed species was assigned to [M–CH₃COO^−^]⁺ and may represent either the parent complex or its monoaquated analog. HPLC‐MS analysis of an aqueous solution of complex **1** revealed a species with *m*/*z* 446.1, which can be assigned to [M–2CH₃COO^−^–H⁺]⁺. No significant decrease in the intensity of this signal was observed during 20 h of incubation. This ion could originate from a diaquated derivative of complex **2**, in which two water molecules are cleaved in the ESI source. However, the same ion may also be formed directly from the parent complex or its monoaquated analog during ionization. Finally, the behavior of complexes **1** and **2** in Rosewell Park Memorial Institute (RPMI) 1640 cell culture medium (without fetal calf serum, FCS) was examined using the same HPLC‐MS method. For both complexes, the amount of intact compound and/or their aquated derivatives decreased rapidly over time (see Supporting Information S2: Figure S21–S22). After 1 h of incubation, the concentrations of complexes **1** and **2** had decreased to approximately 37% and 7%, respectively, as determined by integration of the extracted ion chromatograms corresponding to *m*/*z* 446 ± 0.5 and 602 ± 0.5 (see Supporting Information S2: Figure S23). The main newly‐formed platinum‐containing species detected in MS (positive mode) correspond to adducts with the most abundant amino acids in the medium, namely leucine and/or isoleucine (see Supporting Information S2: Figures S24–S25). In the case of compound **2**, a peak with *m*/*z* 579.2, assigned to [M–2CH_3_COO^−^+Cl^−^] was also detected, suggesting that some exchange of carboxylato ligands with chlorides took place in the medium. Notably, no evidence of NHC ligand cleavage was observed, indicating the stability of the Pt‐carbene bonds under physiologically relevant conditions (pH = 7.2). Overall, these stability studies suggest that dicarboxylatoplatinum(II)‐NHC complexes undergo rapid transformation in cell culture medium via exchange of one or both carboxylato ligands with water molecules or amino acids. The hydrolytic stability of these compounds may, in principle, be further enhanced through appropriate formulation strategies.

### 2.4 In Vitro Cytotoxicity

To evaluate the cytotoxic activity of complexes **1**–**4** in the ovarian carcinoma cell line A2780wt and its cisplatin‐resistant variant A2780cis (which also exhibits cross‐resistance to carboplatin [37]) the MTT (3‐(4,5‐dimethylthiazol‐2‐yl)‐2,5‐diphenyltetrazolium bromide) assay, which measures cellular metabolic activity as an indicator of viability, was conducted. Carboplatin, a second‐generation platinum drug commonly used as part of standard first‐line chemotherapy for ovarian carcinoma [38, 39], was examined as a reference compound due to its clinical relevance. Additionally, the nonmalignant bone marrow stromal cell line HS‐5 was included as a control to assess specificity of the complexes toward cancer cells. The IC_50_ values of the tested compounds are presented in Table 2.

**Table 2:** IC_50_ values (µM) of compounds **1–4** as well as the reference carboplatin on A2780wt, A2780cis, and HS‐5 cells determined with the MTT assay (72 h exposure). Values represent the mean ± standard error of mean (SEM) of four independent experiments.

Compound	Formula	A2780wt	A2780cis	*R*_F_ a	HS‐5
**1**	Pt(NHC)(DMSO)(Ac)_2_	31.13 ± 2.39	36.51 ± 2.85	1.2	64.26 ± 8.20
**2**	Pt(NHC)_2_(Ac)_2_	40.78 ± 2.34	39.28 ± 2.80	1.0	> 50
**3**	Pt(NHC)(DMSO)(DCA)_2_	58.87 ± 2.05	83.12 ± 4.79	1.5	> 100
**4**	Pt(NHC)_2_(DCA)_2_	37.85 ± 1.58	34.86 ± 1.70	0.9	36.95 ± 1.36
Carboplatin	Pt(NH_3_)_2_(CBDC)^b^	33.56 ± 2.10	104.20 ± 11.49	3.1	24.38 ± 0.99

^a^ Resistance factor, *R*_F_ = IC_50_(A2780cis)/IC_50_(A2780wt).

^b^ CBDC is 1,1′‐cyclobutane dicarboxylate.

The new platinum(II)‐NHC complexes **1**–**4** exhibited similar potency in A2780wt cells, with IC_50_ values being within the range observed for the reference drug carboplatin. Interestingly, the number of NHC ligands coordinated to the platinum center and the nature of the carboxylato leaving groups had only minor effects on the cytotoxicity of the NHC complexes. Notably, they demonstrated less pronounced differences in activity between A2780wt cells and their chemoresistant A2780cis counterparts (0.9‐ to 1.5‐fold change) compared with carboplatin (3.1‐fold). This suggests that dicarboxylatoplatinum(II)‐NHC complexes have the potential to overcome ovarian cancer resistance to clinically used platinum‐based chemotherapeutics. Furthermore, they showed some selective toxicity toward cancer cells (particularly at 50 µM), unlike carboplatin (see Supporting Information S2: Figure S26). Interestingly, compound **4** exhibited similar IC_50_ values across the tested cell lines, indicating comparable potency. The concentration‐effect curves of compounds **1**–**4** and carboplatin on A2780wt and A2780cis are displayed in Supporting Information S2: Figures S27–S28.

Finally, it is worth noting that exchanging chlorido ligands for monodentate carboxylates improved the solubility of neutral *N*,*N’*‐diethylbenzimidazol‐2‐ylidene platinum complexes, enabling the assessment of their cytotoxic potential. In contrast, the dichlorido analogs **A** and **B** exhibited limited solubility, preventing their evaluation at concentrations high enough to achieve a 50% reduction in cell viability (IC_50_ values > 25 µM for **A** and IC_50_ values > 6.25 µM for **B** [17]).

Given that platinum‐based drugs are known to commonly induce DNA damage, a comet assay was conducted using A2780wt cells. This assay detects DNA damage such as DNA single/double strand breaks and DNA fragmentation, a common feature of apoptosis [40]. Platinum–NHC complexes have also been shown to interact with DNA, although often in a different way than cisplatin and its analogs, highlighting the relevance of evaluating their potential to induce DNA damage [14, 17, 35]. Compounds **1–4** as well as carboplatin were assessed after an incubation of 48 h at a concentration of 50 µM. This concentration (close to the respective IC_50_ values obtained from the MTT tests) was chosen to ensure comparable testing conditions while also being sufficient for the sensitivity of the assay. Etoposide, a known DNA‐damaging agent, served as a positive control at a concentration of 20 µM with incubation for 4 h, since it induces DNA damage quicker than platinum compounds. DMF (0.1%)‐treated cells were used as a negative control to establish baseline DNA integrity.

**Figure 4:**
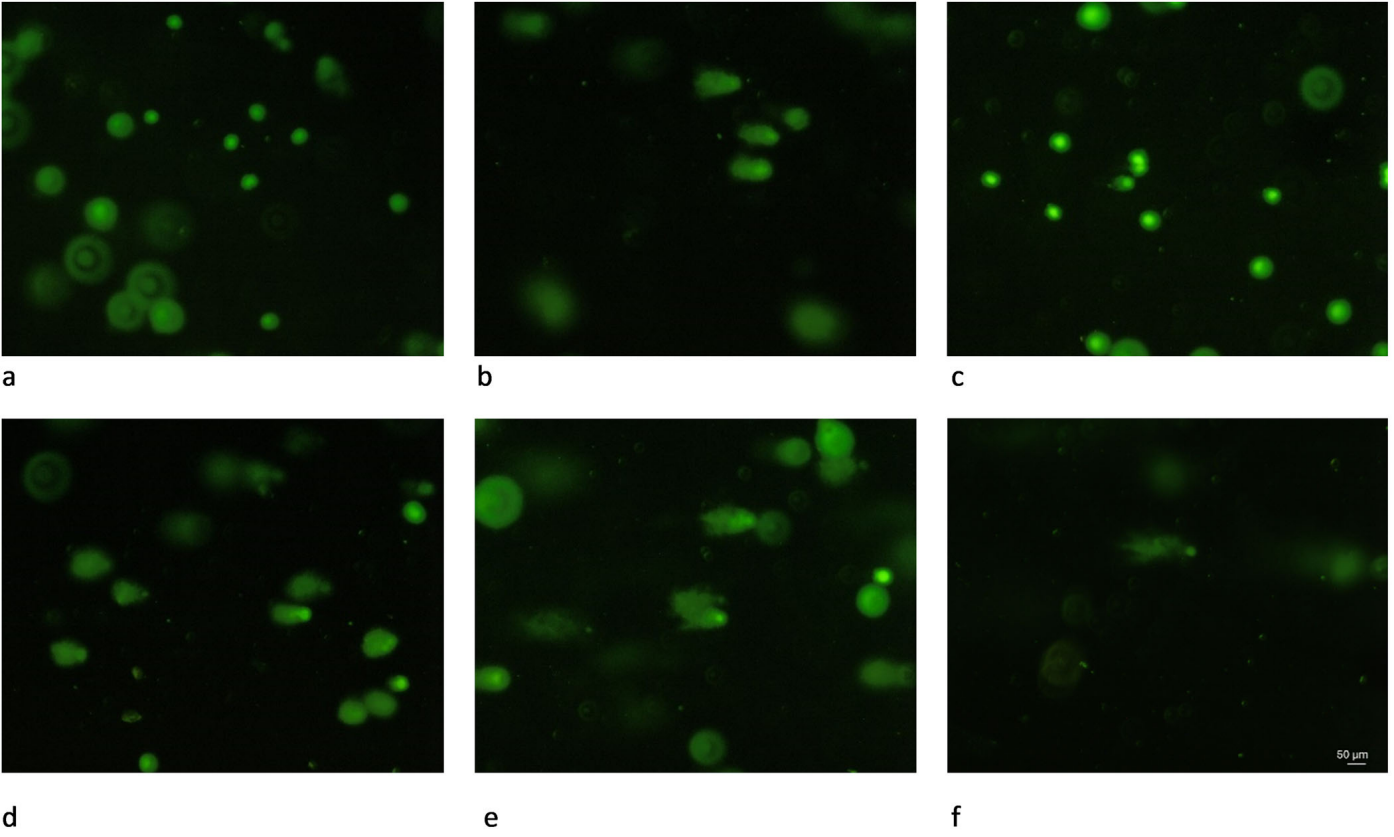
Fluorescence microscopic imaging as part of the comet assay using A2780wt cells after 48 h of incubation with DMF (a), **1** (b), **2** (c), etoposide (d, 4 h of incubation), **3** (e), **4** (f) at a concentration of 50 µM for **1**–**4** and 20 µM for etoposide. Scale bar 50 µm.

As shown in Figure 4, incubation with complexes **1**, **3**, and **4** induced intracellular DNA damage on A2780wt cells, as evidenced by the appearance of comets. This suggests that these compounds are effectively causing DNA strand breaks under the conditions evaluated, either directly or via apoptosis induction. Interestingly, complex **2** (Figure 4c) and carboplatin (Supporting Information S2: Figure S29) did not show any evidence of comet formation, indicating a lack of interaction with the DNA within these cells at the indicated concentration and incubation time.

### 2.5 Loading in BGs

BGs are promising biological carriers for anticancer agents, offering possibly enhanced aqueous solubility, compound stability, and targeted delivery [41, 42, 43, 44]. In this study, we leveraged for the first time the promising characteristics of BGs to encapsulate cytotoxic platinum complexes. Accordingly, compounds **1**–**4** were loaded within BGs derived from two nonpathogenic *E. coli* strains, *EcN* and *Ec* NM522, to potentially improve their hydrolytic stability and facilitate cancer cell delivery. This approach also enables testing at higher concentrations for poorly water‐soluble compounds (e.g., **3** and **4**). Complex and BGs quantities, along with platinum content measured in the BGs after loading, are summarized in Table 3 (see also Section 4).

**Table 3:** Overview of the parameters to create *EcN* and *Ec* NM522 BGs loaded with complexes **1–4**.

Bacterial strain	Compound	Drug^a^ conc (mg/mL)	Drug conc^a^ (mM)	Number of BGs/mL	Pt conc^b^ (mg/mL)	Pt conc^b^ (mM)
*EcN*	**1**	5.00	8.84	0.4 × 10^10^	0.25	1.28
*EcN*	**2**	5.00	7.56	0.4 × 10^10^	0.17	0.87
*EcN*	**3**	5.00	7.11	0.4 × 10^10^	0.22	1.13
*EcN*	**4**	5.00	6.25	0.4 × 10^10^	0.15	0.77
*Ec* NM522	**1**	5.00	8.84	0.2 × 10^10^	0.15	0.77
*Ec* NM522	**2**	5.00	7.56	0.2 × 10^10^	0.09	0.46
*Ec* NM522	**3**	5.00	7.11	0.2 × 10^10^	0.15	0.77
*Ec* NM522	**4**	5.00	6.25	0.2 × 10^10^	0.09	0.46

^a^ Used drug concentration for BGs loading.

^b^ Average platinum content from two independent measurements determined by inductively coupled plasma mass spectrometry (ICP‐MS).

### 2.6 Size and Structure of Loaded BGs by Transmission Electron Microscopy (TEM)

**Figure 5:**
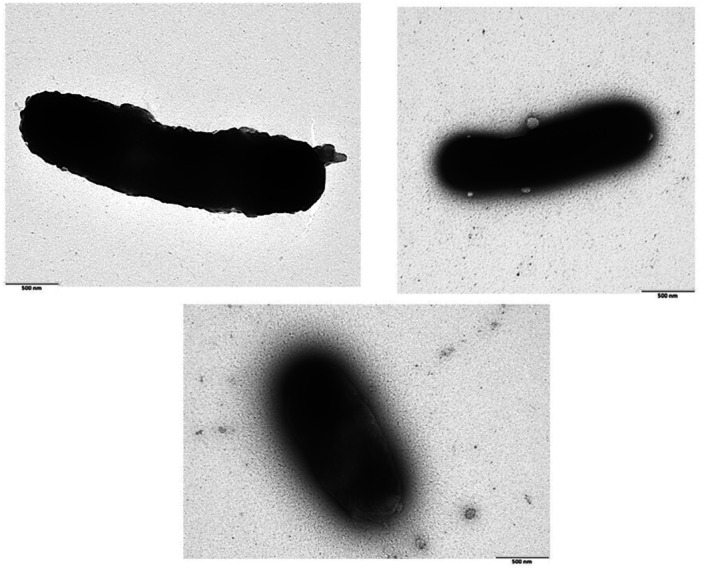
Representative TEM images of compound **1** (top left image) and **3** (top right image) loaded into *EcN*, and non‐loaded *EcN* – bottom image; Scale bar 500 nm.

For the BGs, derived from *EcN* and loaded with the dicarboxylatoplatinum(II)‐NHC complexes, TEM analyses were carried out examining dried‐down BGs suspensions. Most of the loaded BGs showed the typical form and size of the living Gram‐negative *E. coli* counterparts with a few remaining non‐loaded or stuck together. Selected representative images are shown in Figure 5 for loaded *EcN* with complex **1** (Figure 5 top left image), *EcN* with complex **3** (Figure 5 top right image), and non‐loaded *EcN* (bottom).

The presented TEM images of both BGs formulations showed a filled, electron‐dense core compared with the non‐loaded BGs cells, consistent with the high platinum concentrations measured in these formulations (0.25 and 0.22 mg platinum per mL suspension, respectively; see Table 3).

### 2.7 Effect of the Loading in BGs on the Cytotoxicity of Complexes 1–4

To evaluate the impact of filled BGs on the cytotoxic activity, the platinum complexes and their formulations in *Ec* NM522 and *EcN* BGs were tested at 25 µM (adjusted according to platinum concentrations determined by ICP‐MS) using an MTT assay on A2780wt and A2780cis cell lines. Unloaded BGs (4 × 10^8^ *EcN* or 2 × 10^8^ *Ec* NM522, which is equivalent to the highest tested BG concentration) were tested as controls. In these controls, the metabolic activity of the cell lines remained at 80%–90%, confirming that BGs alone are not cytotoxic at the tested concentrations.

**Figure 6:**
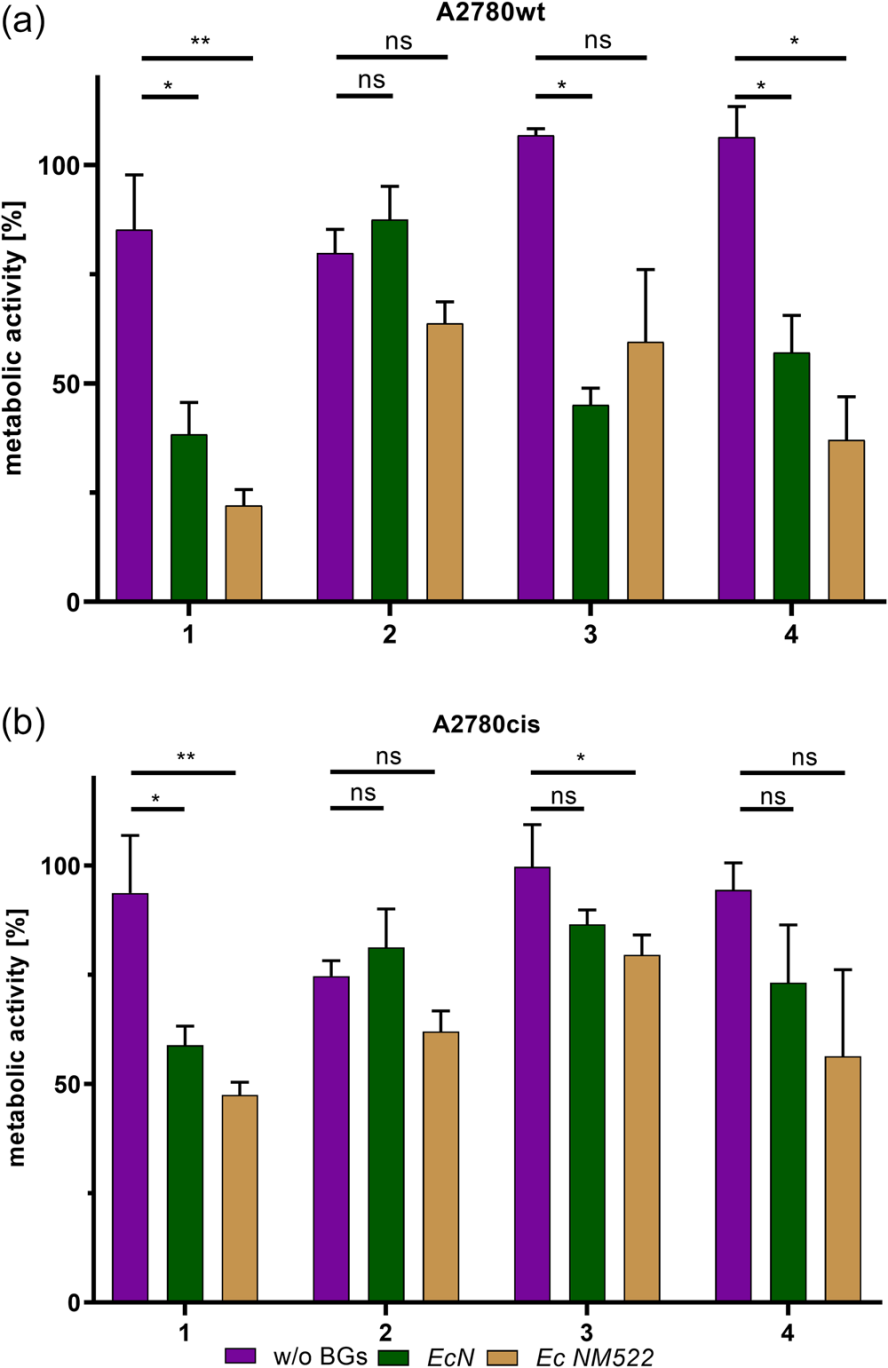
Metabolic activity of A2780wt (top) and A2780cis (bottom) cells after incubation with **1**–**4** in their free (purple) and loaded in *EcN* (green) and *Ec* NM522 (ocher) forms at 25 µM for 72 h. **: very significant (*p* < 0.01) difference between the free and the loaded compounds; *: significant (*p* < 0.05) difference between the free and the loaded compounds; ns: no significant (*p* > 0.05) difference between the free and the loaded compounds. Bars represent the mean + standard deviation (SD) of three independent experiments.

As illustrated in Figure 6, loading of the compounds in either *Ec* NM522 or *EcN* generally enhanced cytotoxic activity in both cell lines. The *Ec* NM522 formulations led to a particularly pronounced reduction in cell metabolic activity across all compounds, with **1** showing the largest difference between free and loaded drugs. Specifically, only 22% of A2780wt cells remained metabolically active after treatment for 72 h with **1** loaded in *Ec* NM522, compared with 85% metabolic activity after treatment with the non‐loaded compound. Interestingly, loading of complex **2** in BGs had a minimal effect on its cytotoxicity in both A2780wt and A2780cis cells. Across both cell lines tested, the cisplatin‐sensitive A2780wt cells exhibited higher sensitivity to the BG formulations of dicarboxylatoplatinum(II)‐(NHC) compounds. Nevertheless, both cell lines displayed consistent cytotoxicity trends: loading the compounds in BGs led to reduced metabolic activity compared with their free forms.

These data indicate that entrapping platinum‐NHC compounds within BGs is a promising formulation approach that improves their cytotoxicity against ovarian cancer cells, especially when loaded in *Ec* NM522. To further investigate the effects of BG formulation on the biological effects of the compounds, studies on cellular accumulation and cell death mechanisms were conducted.

### 2.8 Platinum Accumulation in A2780wt Cells

**Figure 7:**
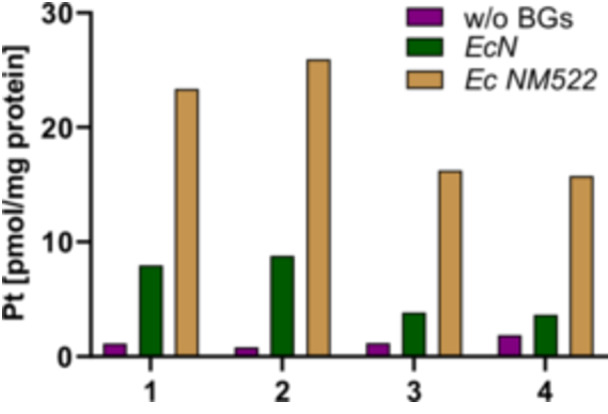
Cellular accumulation of the free complexes and their respective BG formulations, measured as total cellular platinum content by high resolution continuous source atomic absorption spectrometry (HR CS AAS). A2780wt cells were treated for 24 h with 25 µM of compound. The given values represent the mean of two independent experiments.

The extent to which an antitumor drug is taken up by cancer cells can have a considerable impact on its biological effect [45, 46]. The accumulation of complexes **1**–**4** into A2780wt cells was investigated to derive a potential explanation for the different cytotoxicity of the free compounds and those loaded in *EcN* or *Ec* NM522 BGs (Figure 7).

The two complexes **1** and **2**, each carrying two acetato ligands, showed an uptake into A2780wt cells of 1.1 and 0.82 pmol/µg, respectively. The compounds **3** and **4** bearing each two DCA ligands had an uptake of 1.17 and 1.87 pmol/µg, respectively. This increased accumulation (particularly for complex **4**) may be partly explained by the higher lipophilicity associated with the DCA ligands [47] (0.5–0.6 unit increase in calculated log P [19] upon replacing acetato with DCA ligands). The chemical structures of **2** and **4** have an additional 1,3‐dimethylbenzimidazol‐2‐ylidene ligand instead of the DMSO ligand of **1** and **3**. While no clear correlation can be established between the uptake of the free complexes and their structures, some trends emerge upon loading of the compounds within BGs.

As a result of the loading of the four compounds into *EcN*‐derived BGs, an enhancement in uptake can be recognized for all complexes. For **3** (3.84 pmol/µg) the intercellular platinum content increased about 3.5‐times and for **4** (3.64 pmol/µg) it was doubled. For **1** (7.95 pmol/µg) and **2** (8.78 pmol/µg) the improvement was 7‐fold and 10‐fold, respectively.

This augmentation was even stronger after loading with BGs of *Ec* NM522. The accumulation was increased about 14‐fold for **2** (16.23 pmol/µg) and about 8.5‐fold for **4** (15.75 pmol/µg) compared with the free drugs. The intracellular levels of **1** (23.36 pmol/µg) and **2** (25.93 pmol/µg) were elevated approximately 21‐fold and 32‐fold, respectively.

These results document the efficiency of BG formulations for an enhanced delivery of anticancer drugs into tumor cells and are thus in agreement with previous findings [41]. With regard to the structure–activity relationships for the BG‐loaded complexes, replacement of the DMSO ligand with another 1,3‐dimethylbenzimidazol‐2‐ylidene ligand has no considerable effect on the uptake. However, the loaded complexes featuring acetato ligands (**1**, **2**) showed slightly higher accumulation than the corresponding compounds with the DCA ligands (**3**, **4**).

The increased platinum accumulation upon loading in BGs generally correlates with enhanced cytotoxic effects. Loading of complexes **1** and **4** in *Ec* NM522 notably amplified this effect in a more pronounced way. Interestingly, complex **2** demonstrated minimal changes in cytotoxicity after loading, despite the marked increase in cellular uptake. Furthermore, complex **3** followed the same general trend as observed for the other compounds, achieving its highest cellular uptake after loading into *Ec* NM522. However, its cytotoxicity was more pronounced when loaded in *EcN*. Finally, while BG formulations of dicarboxylatoplatinum(II)‐NHC complexes enhanced cellular accumulation, this did not always directly translate into increased cytotoxicity. Several studies indicate that, depending on the bacterial strain used, the resulting BGs are taken up differently by different tumor cells [48, 49]. This phenomenon is likely related to the specific lipopolysaccharide pattern of the BGs' envelopes. It is assumed that some tumor cells, such as melanoma cells, respond to contact with the lipopolysaccharides by increased expression of Toll‐like receptor 4. This leads to increased production of interleukin‐8, which interacts with the adhesion capacity of cancer cells [50].

### 2.9 Induction of Apoptosis

**Figure 8:**
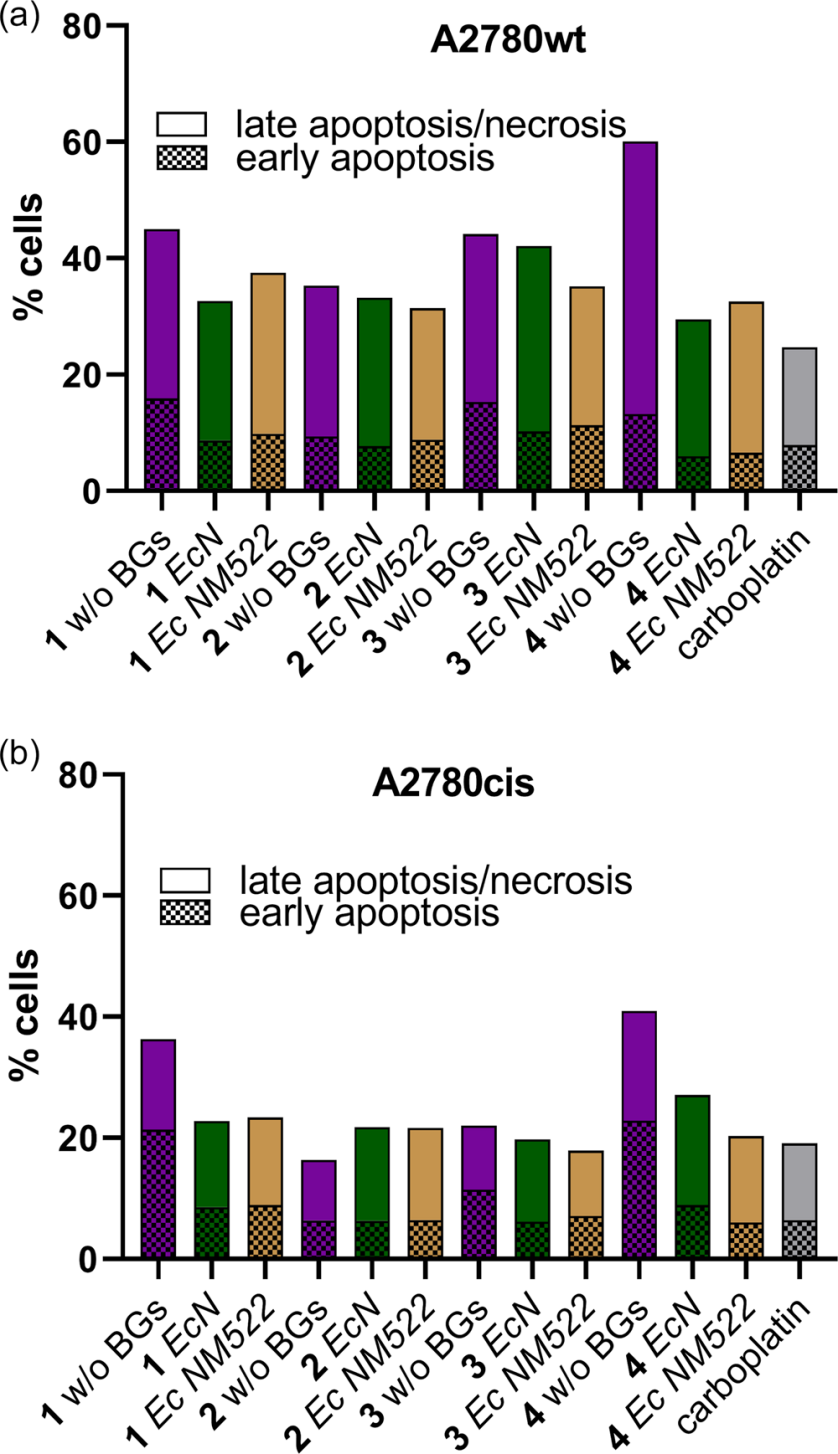
Induction of apoptosis and necrosis in A2780wt (top) and A2780cis (bottom) cells after incubation with **1**–**4** in their free and loaded forms for 24 h at 25 µM and carboplatin as a reference at 50 µM. Bars represent mean of three independent experiments. Error bars were omitted for better visualization, but are given in Supporting Information S2: Tables S5 and S6.

The results of the comet assay indicated interaction of the compounds **1**, **3**, and **4** with the DNA. Therefore, we aimed to evaluate the apoptosis and necrosis inducing potential of complexes **1**–**4** via a fluorescent‐activated cell sorting (FACS) analysis with Annexin V/propidium iodide (PI) staining in A2780wt and A2780cis cells. Cells were treated for 24 h with both the free and BG‐loaded forms of each compound at 25 µM. Carboplatin (50 µM) served as a reference. All compounds, regardless of formulation, induced both apoptosis and necrosis, with necrosis generally being the more prominent type of cell death (Figure 8). Moreover, the ratio of apoptotic to necrotic cells is in most cases comparable to the one observed with carboplatin. These results suggest that the comets induced by the complexes are formed rather due to DNA fragmentation during apoptosis than direct damage of the DNA.

Furthermore, caspase‐3 activation serves as a primary hallmark of apoptosis. Therefore, we evaluated this induction with a luminescence‐based caspase‐3 activity assay on A2780wt cells. The evaluation was conducted following a 24 h treatment with the complexes (alone and loaded in the respective BGs) at a concentration of 25 µM, as well as with the non‐loaded BGs. As given in Supporting Information S2: Table S7, most compounds showed a noteworthy increase in caspase‐3 induction, especially the complexes which were loaded in *Ec* NM522. This suggests that loading within *Ec* NM522 may enhance the proapoptotic effects of these compounds on A2780wt cells, potentially due to improved targeted delivery and higher content of the entrapped compound.

### 2.10 ICD

As a second potential mode of cell death, ICD has been investigated. Thus, two hallmarks of ICD, namely release of adenosine triphosphate (ATP) and calreticulin translocation to the plasma membrane have been analyzed in detail. Given that some platinum complexes, including the clinically used drug oxaliplatin [27], as well as BGs, are known inducers of ICD, we investigated the potential of BGs loaded with platinum(II)‐NHC complexes to trigger this process. As a first step, a luminescence‐based ATP assay was performed on A2780wt and A2780cis cells, treated with compounds **1**–**4**, alone and loaded in the respective BGs, as well as the non‐loaded BGs. Untreated cells (without compound and without BGs) were tested as negative controls. The ATP levels are presented in Table 4.

**Table 4:** ATP concentration (µM) in A2780wt and A2780cis cells after treatment with the free and loaded compounds at a concentration of 25 µM. Values represent the mean ± SEM of three independent experiments.

Cell line	Compound	w/o BGs	*EcN*	*Ec* NM522
A2780wt	w/o compound	0.29 ± 0.04	0.39 ± 0.00	0.28 ± 0.04
	**1**	0.20 ± 0.04	0.24 ± 0.06	0.18 ± 0.06
	**2**	0.32 ± 0.03	0.42 ± 0.03	0.40 ± 0.03
	**3**	0.15 ± 0.08	0.33 ± 0.07	0.33 ± 0.04
	**4**	0.32 ± 0.13	0.36 ± 0.05	0.27 ± 0.05
A2780cis	w/o compound	0.26 ± 0.03	0.32 ± 0.06	0.32 ± 0.03
	**1**	0.23 ± 0.05	0.36 ± 0.05	0.34 ± 0.03
	**2**	0.31 ± 0.04	0.35 ± 0.06	0.36 ± 0.05
	**3**	0.28 ± 0.04	0.33 ± 0.03	0.34 ± 0.03
	**4**	0.10 ± 0.03	0.30 ± 0.04	0.28 ± 0.03

In general, ATP levels were very low. However, in A2780cis cells, treatment with both free and loaded compounds led to a modest increase in ATP levels, except for free compounds **1** and **4**. BG formulations induced a higher ATP content than their free counterparts, with empty BGs also causing slight ATP elevation, suggesting a potential role of BGs in ICD induction. In A2780wt cells, the effect of the compounds on ATP levels varied; however, none of the treatments resulted in significant overall changes.

Hence, to further evaluate the potential of the compounds to induce ICD, surface calreticulin levels were measured after incubation with complexes **1**–**4**, either alone or loaded in BGs, as well as with the non‐loaded BG strains themselves on A2780wt and A2780cis cells. Oxaliplatin, a known metal‐based ICD inducer, was included for comparison. The mean intensities of > 20 cells per condition were evaluated via immunofluorescent staining and the x‐fold change in intensity compared with untreated cells (without compound and without BGs) are displayed in Table 5 and representative images of the stained cells are depicted in Supporting Information S2: Figure S30.

**Table 5:** x‐fold intensity of surface calreticulin compared to untreated cells after treatment of A2780wt and A2780cis cells with compounds **1–4** free and loaded at a concentration of 25 µM, respectively. At least 40 cells from 2 independent experiments were quantified and data are shown as mean ± SD.

Cell line	Compound	w/o BGs	*EcN*	*Ec* NM522
A2780wt	w/o compound	1.00 ± 0.00	1.14 ± 0.26	1.41 ± 0.32
	**1**	1.09 ± 0.00	1.35 ± 0.09	0.95 ± 0.24
	**2**	1.07 ± 0.12	1.25 ± 0.04	1.18 ± 0.05
	**3**	1.50 ± 0.17	1.08 ± 0.21	1.26 ± 0.16
	**4**	1.37 ± 0.24	1.34 ± 0.13	1.69 ± 0.19
	oxaliplatin	0.86 ± 0.24	—	—
A2780cis	w/o compound	1.00 ± 0.00	0.89 ± 0.24	0.95 ± 0.05
	**1**	1.18 ± 0.37	0.82 ± 0.06	1.14 ± 0.19
	**2**	1.02 ± 0.05	0.93 ± 0.13	1.07 ± 0.05
	**3**	0.75 ± 0.28	0.77 ± 0.06	0.79 ± 0.10
	**4**	1.13 ± 0.36	0.95 ± 0.55	1.03 ± 0.29
	oxaliplatin	1.36 ± 0.24	—	—

In A2780wt cells, most compounds induced a slight increase in surface calreticulin exposure, with no significant difference between free and loaded forms. Similarly, in A2780cis cells treatment with free complexes **1**, **2**, and **4**, as well as with their formulation in *Ec* NM522, resulted in a slight rise in surface calreticulin. Interestingly, loading in *EcN* resulted in marginally diminished calreticulin exposure. In the case of compound **3**, surface calreticulin was slightly reduced for all formulations. Oxaliplatin also only faintly altered the surface calreticulin exposure in both cell lines at these experimental conditions.

These results highlight that the loading strategy and the specific molecular characteristics of the compounds tested only moderately influence ATP concentration and calreticulin exposure in both wild‐type and cisplatin‐resistant cells. Hence, there is little evidence that the compounds tested cause ICD. However, ICD is a complex process better studied in vivo or in in vitro systems where cancer cells and immune cells are coincubated.

It has been previously shown that systemic administration of attenuated bacteria triggers a strong Th1‐dominated immune response in the tumor area via polarized T‐cells [51]. More recently Groza et al. demonstrated the distinct immuno‐stimulatory effect of the same BGs used as an adjuvant to oxaliplatin treatment of colorectal cancer (CRC) in CT26 allografts. The application of empty *EcN* BGs exerted a strong synergistic effect on an enhanced induction of ICD and activation of an efficient T‐cell response leading to long‐term antitumor memory effects [29]. Thus, further in vitro and in vivo studies are needed within the framework of future projects to elucidate the potential involvement of ICD in the mechanism of action of BG‐loaded platinum(II)‐NHC complexes.

The two *E. coli*‐derived BGs used in this study are of non‐pathogenic origin and can be considered unlikely to trigger harmful immune responses. Numerous in vitro and in vivo studies have already proven that these BGs are nontoxic and well tolerated [52, 53]. As mentioned above, Groza et al. applied BGs derived from the same strains and demonstrated their immunostimulating effect in mice with intraperitoneal carcinomatosis of CRC [29]. Moreover, both *EcN* and *Ec* NM522 possess intact surface structures, allowing the attachment to mammalian cells [43]. Thus, they are capable of inducing a beneficial immunostimulatory response in the context of cancer therapy, while there are no studies indicating that these BGs could provoke harmful immunogenic responses in humans. In addition, *EcN* (O6:K5:H1) has been used as a probiotic agent in medicine since the early 1920s [54, 55], and Westendorf et al. further demonstrated its potential as a safe carrier for therapeutic molecules [56].

Altogether, BGs derived from nonpathogenic *E. coli* strains represent a safe and powerful tool to stimulate antitumor immunity and hold promise as an effective drug delivery system for the treatment of various types of cancers.

## 3 Conclusion

Four new *N,N′*‐diethylbenzimidazol‐2‐ylidene platinum(II) complexes featuring monodentate carboxylato ligands were synthesized and characterized in detail using ESI‐HRMS, NMR spectroscopy, X‐ray crystallography, and elemental analysis. HPLC‐MS analysis indicated that carboxylato leaving groups undergo time‐dependent ligand exchange with amino acids in cell culture medium, while the NHC ligand(s) remained coordinated to the platinum center. The compounds showed cytotoxicity comparable to carboplatin against the cisplatin‐sensitive A2780wt ovarian carcinoma cell line and, notably, retained similar potency in the chemoresistant A2780cis variant. They also demonstrated selective toxicity toward cancer cells over nonmalignant bone marrow stromal cells (HS‐5).

Loading of the dicarboxylatoplatinum(II)‐NHC complexes in BGs derived from the two *E*. *coli* strains *Ec* NM522 and *EcN* significantly enhanced their accumulation in ovarian cancer cells and increased overall cytotoxicity. All compounds, regardless of the formulation, induced apoptosis, with apoptotic‐to‐necrotic cell ratios similar to those observed for carboplatin. To the best of our knowledge, this is the first study that provides BG formulations of anticancer platinum complexes. The high payload of the anticancer drug within the BGs may enhance biocompatibility and reduce toxicity, rendering these novel formulations suitable for clinical applications.

In summary, the use of appropriate carboxylato ligands improved the solubility of platinum(II)‐NHC complexes without compromising cytotoxicity against cancer cells. However, further optimization of the leaving groups and/or formulation strategies may be needed to enhance aqueous stability. Loading in BGs emerges as a promising approach for delivering platinum(II)‐NHC complexes to cancer cells.

## 4 Experimental

### 4.1 Chemistry

#### 4.1.1 General

Chemical reagents and solvents were obtained from commercial suppliers and were used without additional purification. Non‐loaded BGs of the probiotic *EcN* and the nonpathogenic *Ec* NM522 strains were provided by BIRD‐C GmbH, Kritzendorf, Austria. NMR spectra were recorded on a Bruker Avance 4 Neo 400 MHz spectrometer (Bruker, Billerica, MA, USA) at 400.13 (^1^H), 100.62 (^13^C) and 85.88 (^195^Pt) MHz in CDCl_3_ at an ambient temperature. Chemical shifts (δ) are reported in ppm and referenced to the residual solvent peaks for ^1^H (CDCl_3_, 7.26 ppm) and ^13^C (CDCl_3_, 77.2 ppm) NMR spectroscopy. ^195^Pt NMR spectra were referenced to external Na_2_[PtCl_6_] (set to 0 ppm). Signal multiplicities are denoted as singlet (s), doublet (d), triplet (t), or multiplet (m). Coupling constants (*J*) are given in Hertz (Hz). High‐resolution mass spectra (HRMS) were acquired using an Orbitrap Elite mass spectrometer (Thermo Fisher Scientific, Waltham, MA, USA) with direct infusion and electrospray ionization in positive ion mode, using ACN as solvent. NMR and HRMS data were processed using MestreNova 12.0 software. Elemental analyses (C, H, N) were conducted at the Department of General, Inorganic and Theoretical Chemistry, University of Innsbruck, using a vario micro cube elemental analyzer (Elementar Analysensysteme GmbH, Langenselbold, Germany). The results were within ±0.4% of the calculated values, confirming a purity of ≥ 95% for the compounds analyzed.

The InChI codes of the investigated compounds, together with some biological activity data, are provided as Supporting Information.

#### 4.1.2 Synthesis of Dichloridoplatinum(II)‐NHC Complexes A and B

A general reaction scheme is given in Figure 1. Dichloridoplatinum(II)‐NHC complexes **A** and **B** were synthesized from *cis*‐[Pt(DMSO)_2_Cl_2_] as described previously [17, 57]. Silver dichloroacetate was prepared by reacting aqueous solutions of dichloroacetic acid, neutralized with NaOH, and AgNO_3_ [58].

#### 4.1.3 General Procedure for the Synthesis of Dicarboxylatoplatinum(II)‐NHC Complexes (1–4)

Dichloridoplatinum complexes (**A** or **B**) were dissolved in DCM, followed by addition of 2.8–3.0 equiv. of the respective silver carboxylate. The reaction mixture was stirred at rt for 48 h under light protection. Afterward, the insoluble silver salts were removed by filtration over celite yielding a clear solution. The filtrate volume was reduced by rotary evaporation to approximately 1–2 mL, and diethyl ether (Et_2_O) was added to precipitate the final product as a white solid. The latter was collected via filtration, washed with Et_2_O and dried in vacuum.

(*SP‐4‐2*)‐Diacetato(1,3‐diethylbenzimidazol‐2‐ylidene)(dimethylsulfoxide)platinum(II) (**1**): **A** (64 mg, 0.123 mmol) in DCM (20 mL), silver acetate (61 mg, 0.366 mmol, 3.0 equiv.). Yield: 57 mg (82%). ^1^H NMR (400 MHz, CDCl_3_): *δ* 7.47–7.43 (m, 2H, Ar‐H), 7.32–7.28 (m, 2H, Ar‐H), 4.97 (m, 2H, CH_2_), 4.88 (m, 2H, CH_2_), 3.41 (s, 6H, (CH_3_)_2_SO), 2.05 (s, 3H, CH_3_‐Ac), 1.84 (s, 3H, CH_3_‐Ac), 1.62 (t, *J* = 7.2 Hz, 6H, CH_3_). ^13^C NMR (101 MHz, CDCl_3_): *δ* 178.2 (COO), 176.2 (COO), 149.7 (NCN), 133.5 (Ar‐Cq), 123.6 (Ar‐CH), 111.4 (Ar‐CH), 46.0 ((CH_3_)_2_SO), 44.0 (CH_2_), 24.4 (CH_3_‐Ac), 22.9 (CH_3_‐Ac), 14.5 (CH_3_). ^195^Pt NMR (86 MHz, CDCl_3_): *δ* −3266. ESI‐HRMS found (calcd.): [M‐CH_3_COO^−^]^+^, 506.1079 (506.1077); [2M‐CH_3_COO^−^]^+^, 1071.2291 (1071.2190). Elemental Analysis calcd. for C_17_H_26_N_2_O_5_PtS: C 36.10, H 4.63, N 4.95. Found: C 36.12, H 4.84, N 4.86.

(*SP‐4‐2*)‐Diacetatobis(1,3‐diethylbenzimidazol‐2‐ylidene)platinum(II) (**2**): **B** (60 mg, 0.097 mmol) in DCM (30 mL), silver acetate (50 mg, 0.300 mmol, 3.0 equiv.). Yield: 60 mg (92%). ^1^H NMR (400 MHz, CDCl_3_): *δ* 7.35–7.31 (m, 4H, Ar‐H), 7.24–7.20 (m, 4H, Ar‐H), 4.97 (m, 4H, CH_2_), 4.73 (m, 4H, CH_2_), 1.94 (s, 6H, CH_3_‐Ac), 1.26 (t, *J* = 7.2 Hz, 12H, CH_3_). ^13^C NMR (101 MHz, CDCl_3_): *δ* 176.8 (COO), 155.6 (NCN), 133.5 (Ar‐Cq), 123.3 (Ar‐CH), 110.9 (Ar‐CH), 43.3 (CH_2_), 24.1 (CH_3_‐Ac), 14.0 (CH_3_). ^195^Pt NMR (86 MHz, CDCl_3_): δ –3448. ESI‐HRMS found (calcd.): [M‐CH_3_COO^−^]^+^, 602.2108 (602.2091); [M‐2CH_3_COO^−^‐H^+^]^+^, 542.1895 (542.1880); [2M‐CH_3_COO^−^]^+^, 1263.4357 (1263.4225). Elemental Analysis calcd. for C_26_H_34_N_4_O_4_Pt·0.5H_2_O: C 46.56, H 5.26, N 8.35. Found: C 46.47, H 5.23, N 8.07.

(*SP‐4‐2*)‐Bis(dichloroacetato)(1,3‐diethylbenzimidazol‐2‐ylidene)(dimethylsulfoxide)platinum(II) (**3**): **A** (70 mg, 0.135 mmol) in DCM (18 mL), silver dichloroacetate (89 mg, 0.377 mmol, 2.8 equiv.). Yield: 76 mg (80%). ^1^H NMR (400 MHz, CDCl_3_): *δ* 7.51–7.46 (m, 2H, Ar‐H), 7.37–7.33 (m, 2H, Ar‐H), 5.98 (s, 1H, CHCl_2_), 5.79 (s, 1H, CHCl_2_), 4.93 (m, 4H, CH_2_), 3.45 (s, 6H, (CH_3_)_2_SO), 1.65 (t, *J* = 7.2 Hz, 6H, CH_3_). ^13^C NMR (101 MHz, CDCl_3_): *δ* 169.5 (COO), 168.5 (COO), 145.3 (NCN), 133.4 (Ar‐Cq), 124.1 (Ar‐CH), 111.6 (Ar‐CH), 68.5 (CHCl_2_), 66.8 (CHCl_2_), 46.0 ((CH_3_)_2_SO), 44.3 (CH_2_), 14.7 (CH_3_). ^195^Pt NMR (86 MHz, CDCl_3_): δ −3293. ESI‐HRMS found (calcd.): [M‐CHCl_2_COO^−^]^+^, 575.0291 (575.0275). Elemental Analysis calcd. for C_17_H_22_Cl_4_N_2_O_5_PtS·0.2H_2_O: C 28.88, H 3.19, N 3.96. Found: C 28.80, H 3.25, N 3.81.

(*SP‐4‐2*)‐Bis(dichloroacetato)bis(1,3‐diethylbenzimidazol‐2‐ylidene)platinum(II) (**4**): **B** (81 mg, 0.132 mmol) in DCM (30 mL), silver dichloroacetate (88 mg, 0.373 mmol, 2.8 equiv.). Yield: 95 mg (90%). ^1^H NMR (400 MHz, CDCl_3_): *δ* 7.40–7.36 (m, 4H, Ar‐H), 7.30–7.25 (m, 4H, Ar‐H), 5.91 (s, 2H, CHCl_2_), 4.92 (m, 4H, CH_2_), 4.78 (m, 4H, CH_2_), 1.34 (t, *J* = 7.2 Hz, 12H, CH_3_). ^13^C NMR (101 MHz, CDCl_3_): *δ* 166.8 (COO), 151.3 (NCN), 133.5 (Ar‐Cq), 123.8 (Ar‐CH), 111.1 (Ar‐CH), 68.4 (CHCl_2_), 43.5 (CH_2_), 14.4 (CH_3_). ^195^Pt NMR (86 MHz, CDCl_3_): *δ* –3438. ESI‐HRMS found (calcd.): [M‐CHCl_2_COO^−^]^+^, 671.1308 (671.1298); [M‐2CHCl_2_COO^−^‐H^+^]^+^, 542.1889 (542.1880). Elemental Analysis calcd. for C_26_H_30_Cl_4_N_4_O_4_Pt: C 39.06, H 3.78, N 7.01. Found: C 38.96, H 3.87, N 6.92.

### 4.2 X‐Ray Crystallography

Single‐crystal X‐ray diffraction measurements were carried out under a stream of cold N_2_ on a Bruker D8 Quest diffractometer (Bruker, Billerica, MA, USA) equipped with an Incoatec Microfocus source generator (multi layered optics monochromatized Mo‐K_α_ radiation, *λ* = 71.073 pm) and a Photon III C14 detector. Multi‐scan absorption corrections were applied with the program SADABS‐2014/5. Structure solution and refinement were performed with SHELXT and SHELXL programs [59], respectively. Detailed information on the crystal data, data collection parameters, and structure refinement are given in the Supporting Information (see Supporting Information S2: Tables S1–S4). Additional crystallographic data can be obtained free of charge from the Cambridge Crystallographic Data Center (CCDC 2449410‐2449413).

### 4.3 Stability Studies

Stability of the complexes **1**–**4** in CDCl_3_, CD_3_OD, CD_3_CN, DMF‐d_7_, and D_2_O was monitored by ^1^H NMR spectroscopy at ambient temperature. Solutions of the complexes (approximately 3 mg/mL) were prepared in each solvent, and ¹H NMR spectra were recorded periodically over 24 h. Chemical shifts (δ) were referenced to the residual solvent peaks: CDCl_3_ (7.26 ppm), DMF‐d_7_ (2.92 ppm, lowfield methyl signal), D_2_O (4.79 ppm), CD_3_OD (4.87 ppm) and CD_3_CN (1.94 ppm).

Additional stability studies in aqueous media and RPMI 1640 (without FCS) were carried out using RP‐HPLC‐MS. For this purpose, 0.5 mM solutions of complexes **1** and **2** were prepared in Milli‐Q water and RPMI 1640. Samples were analyzed by HPLC‐MS immediately after preparation and at selected time points during 24 h of incubation at rt. HPLC‐MS measurements were performed on Agilent 1260 system hyphenated to an MS Single Quad detector from Agilent with ESI (LC‐MSD from Agilent Technologies, Santa Clara, CA, USA). As stationary phase, a Gemini C18 110 Å; 150 × 4.6 mm; 3.0 µm particles (Phenomenex Ltd.; Aschaffenburg, Germany) column was employed and equipped with a 4 × 3 mm guard column of the identical material. The mobile phase flow was set to 0.8 mL/min; and consisted of a mixture of water (solvent A) – MeOH (solvent B) using gradient elution (0 min 5% B; 3 min 5% B; 17 min 98% B; 20 min stop; post time 10 min). The oven temperature was maintained at 30°C and the MS analysis was performed with split flow 1:5 of the eluate in positive ionization mode (capillary voltage 4.5 kV; source temperature 320°C; dry gas 10 L/min; nebulizer gas pressure 30 psi, scan range *m*/*z* 50–1500). The injection volume of the sample solution (*V*_sample_) was set to 10 µL. Sample degradation was evaluated by integration of the extracted ion chromatograms at the indicated *m*/*z* ± 0.5 (based on the monoisotopic peaks).

### 4.4 Loading of BGs

*EcN* and *Ec* NM522 BGs (10 mg each; BIRD‐C GmbH, Kritzendorf, Austria) were resuspended in 1 mL complex solution (5.0 mg platinum(II)‐complex dissolved in 1.0 mL MeOH), respectively. The suspensions were stirred for 1.5 h at rt. After stirring, loaded BGs were collected via centrifugation at 11,300*g* for 10 min at 15°C. The supernatants were discarded and the pellets, containing the loaded BGs, were washed three times with MilliQ water and collected. Subsequently, the washed BGs were resuspended in 1 mL Milli‐Q water and stored at –20°C for further analyses.

### 4.5 Inductively Coupled Plasma Mass Spectrometry

The amount of loaded complex was evaluated by determination of platinum inside the BGs using ICP‐MS and after heat assisted digestion according to a modified protocol from Ratzinger et al. [60]: 100 μL of aliquots of freshly prepared BGs were digested with 3 mL of subboiled nitric acid (20%, *v*/*v*) applying an established automated microwave system. The digested samples as well as the supernatants were then diluted with Milli‐Q water and the concentration of platinum was determined using an Agilent 7500ce ICP‐MS instrument (Agilent Technologies, Waldron, Germany) equipped with a Cetac ASX‐520 auto sampler, a Micro Mist nebulizer and nickel cones. Samples were prepared by dilution in the ppm concentration range with nitric acid (3%) and the addition of indium as an internal standard. The average concentrations and SD were calculated from the different measured isotopes of platinum.

### 4.6 TEM

For *EcN*‐derived BGs, both loaded with the platinum complexes or non‐loaded, TEM analyses were carried out examining BGs suspensions. Formvar‐ and carbon‐coated grids (200 mesh, Cu) were glow discharged (Quorum GloQube) and incubated for 1 min at rt with 4 µL of non‐diluted BGs suspensions. Excess liquid was removed with a filter paper (Whatman) and immediately replaced by a drop of 2.5% gadolinium acetate (single drop method). The contrasted grids were viewed and imaged with a Zeiss EM900N (ZEISS, Germany) transmission electron microscope at 80 kV.

### 4.7 Cell Lines

The ovarian carcinoma cell lines A2780wt and A2780cis were kindly provided by the Department of Gynaecology, Medical University of Innsbruck, Austria. To maintain cisplatin resistance, A2780cis cells were treated every second week with 1 µM of cisplatin. The stromal bone marrow cell line HS‐5 was purchased from the American Type Culture Collection (ATCC, Manassas, Virginia, USA). The cell lines were cultivated in RPMI 1640 medium without phenol red (PAN Biotech, Aidenbach, Germany), supplemented with l‐glutamine (2 mM), 100 μg/mL of penicillin, 100 μg/mL of streptomycin, and FCS (10%) (all from Invitrogen Corporation, Gibco, Paisley, Scotland) at 37°C in a 5% CO_2_/95% air atmosphere and passaged twice weekly.

### 4.8 Analysis of Cell Growth Inhibition

The exponentially growing cells were seeded at a density of 8 × 10^3^ cells/well (A2780wt/cis) or 1 × 10^4^ cells/well (HS‐5), respectively, into clear flat‐bottom 96‐well plates in triplicates. Following 24 h of incubation for the adherence of the cells at 37°C in a humidified atmosphere (5% CO_2_/95% air), the test compounds were added. Stocks solutions were freshly prepared in RPMI 1640 or in DMF (for the less water‐soluble substances), and diluted with RPMI 1640 (final DMF concentrations did not exceed 0.1% *v*/*v*) to reach the desired concentrations between 10 and 100 µM. For the evaluation of the cell growth inhibition by the compounds loaded in BGs, the BG suspensions were directly diluted in RPMI 1640 to the desired testing concentration (25 µM). After another 72 h of incubation, the cellular metabolic activity was measured as an indicator of cell viability employing an MTT assay. Hereby, 20 µL of a 5 mg/mL MTT solution was added to each well, respectively. After 3 h of incubation, the medium was discarded, and the formazan crystals formed were dissolved in DMSO for 15 min with gentle shaking. Afterward, the optical density (OD) was measured at 595 and 620 nm, respectively on an EnSpire Multimode Plate Reader (revvity, Waltham, MA, USA). The OD at 620 nm was subtracted from the OD at 595 nm. The OD of the medium was subtracted to exclude the unspecific staining caused by FCS‐containing medium. Cells treated with vehicle control (0.1% DMF) only were set at 100% metabolic activity. The IC_50_ values were calculated with Prism 8.0 (GraphPad Software Inc., Boston, MA, USA) using nonlinear regression. Significance was evaluated via a one sample *t*‐test performed with Prism 8.0.

### 4.9 Comet Assay

Cellular DNA damage can be visualized by employing a comet assay, which is a single cell gel electrophoresis assay. Hereby, A2780wt cells were seeded at a density of 2 × 10^6^ into 25 cm^2^ flasks. After letting the cells adhere for 24 h in a humidified atmosphere, the cell culture medium was exchanged for medium containing the respective compound. The cells were then incubated for 48 h with the platinum complexes or 4 h with etoposide, respectively. After incubation, the cell growth was stopped by removing the compound‐containing medium, washing the cells with phosphate‐buffered saline (PBS) and detaching them with a rubber policeman. The detached cells were then centrifuged at 700*g* for 2 min and the resulting pellet was then washed with 5 mL of PBS. After that, the cells were adjusted to a concentration of 1 × 10^6^ cells/mL and were resuspended in ice cold PBS. A total of 75 µL of liquified agarose per condition was transferred onto the slide to create a base layer. This base layer was solidified by transferring the slide to 4°C for 15 min. Afterwards, 10 µL of the above‐mentioned cell suspension in PBS were mixed with 90 µL of agarose. A total of 75 µL of this was added to each base layer, respectively. This layer was also allowed to solidify at 4°C for 15 min. The slide was then transferred to a small basin which contained pre‐chilled lysis buffer (14.6 g NaCl, 20 mL EDTA solution, 10 mL lysis solution, 10 mL DMSO, 60 mL deionized H_2_O), where it remained for 45 min in the dark at 4°C. The lysis buffer was then aspirated and exchanged with a pre‐chilled alkaline solution (1.2 g NaOH, 0.2 mL EDTA solution, 100 mL deionized H_2_O), where it remained for further 30 min in the dark at 4°C. The slide was then transferred to a horizontal electrophoresis chamber which was filled with cold alkaline solution. The electrophoresis conditions were set to 1.5 V/cm and 300 mA for 20 min. After the electrophoresis, the slide was transferred into a small basin containing pre‐chilled, deionized H_2_O. After 2 min, the water was aspirated and this washing step was repeated twice more. After the final rinse, the slide was submerged in 70% ethanol for 5 min. Once the slide has dried, 100 µL of diluted Vista Green DNA Dye was added to each well. After 15 min of incubation, the slides were analyzed on an IX‐70 epifluorescence microscope (Olympus, Shinjuku, Japan) using a FITC filter.

### 4.10 Cellular Accumulation Studies

For cellular uptake studies, 2.5 × 10^6^ of A2780wt cells in their exponential growing phase were seeded in 25 cm^2^ cell culture flasks and were allowed to adhere for 24 h at 37°C in a humidified atmosphere (5% CO_2_/95% air). Stock solutions of the platinum complexes in DMF were freshly prepared and the stocks for the loaded compounds were freshly thawed. The stocks were then diluted with RPMI 1640 containing 10% FCS to the desired concentration (final DMF concentration of 0.1% (*v*/*v*), final platinum complex concentration of 25 μM). The medium in the cell culture flasks was replaced with 4 mL of fresh cell culture medium containing the compounds and the flasks were incubated at 37°C and 5% CO_2_/95% air for 24 h. Cell pellets were prepared by removing the medium, washing twice with PBS, and detaching the cells with accutase (GE Healthcare, Chicago, IL, USA). Subsequently, cells were pelleted by centrifugation (4°C, 380*g*, 3 min). Each pellet was twice washed with PBS and then stored at −20°C until further analyses.

Cellular accumulation was quantified by measuring the total platinum content using HR CS AAS. Accordingly, cell pellets were thawed, resuspended in ultrapure water (300 µL; Siemens LaboStar, Günzburg, Germany), and sonicated (20 s, nine cycles, 80%–85% power; Bandelin Sonoplus, Berlin, Germany). The resulting lysates were analyzed using AAS at the wavelength *λ* = 265.9450 nm with a high resolution continuous source (HR CS) spectrometer (contrAA 700; Analytik Jena, Jena, Germany). The ASpect CS software (version 2.3.1.0; Analytik Jena, Jena, Germany) was used to handle the spectrometer. The graphite furnace (GF) sub‐technique was used for this. In the latter, atomization takes place electrothermally in pyrolytically coated graphite tubes bearing an integrated platform (Analytik Jena, Jena, Germany). The GF program is outlined in Supporting Information S2: Table S8. The samples stored in polystyrene vials (0.5 mL; Gesellschaft für Analysentechnik, Salzwedel, Germany) were injected onto this platform employing an MPE 60 autosampler (Analytik Jena, Jena, Germany). As the purge and the protective gas served argon (Alphagaz 1, 99.999%, Air Liquide, Düsseldorf, Germany). The mean integrated absorbance of three injections was adduced during all measurements. The calibration was performed with cisplatin (Sigma‐Aldrich, Taufkirchen, Germany) covering one blank and five standards (2−10 μg/L, *R* ^2^ ≥ 0.9929). As the degree of cellular accumulation is indicated as amount of compound (pmol) in the cell lysate relative to its protein mass (mg), the protein content of each sample was determined [61]. Therefore, the method described by Bradford was used. One blank (ultrapure water only) and six aqueous calibration standards made of albumin fraction V (Carl Roth, Karlsruhe, Germany) were used for the calibration (*R* ^2^ ≥ 0.9926). Each 20 µL of either albumin standards or the cell lysates were pipetted into a 96‐well plate (Sarstedt, Nürmbrecht, Germany), followed by adding 200 µL of Bradford's reagent (Roti‐Nanoquant, Carl Roth, Karlsruhe, Germany; diluted 1:5 with ultrapure water). After incubating (5 min, rt), the absorbance (*λ* = 595 nm) was read with a microplate reader (Tecan Infinite M 1000 Pro, Männedorf, Switzerland). All calibration standards and lysates were measured in duplicates.

### 4.11 Annexin V/PI Staining and FACS Analysis

To evaluate the mode of cell death, a FACS measurement after staining with Annexin V and PI was conducted. First, 0.3 × 10^6^ A2780wt or A2780cis cells were seeded into 12‐well plates and were allowed to adhere for 24 h. The compounds were added at 25 µM, either diluted from a freshly prepared DMF stock solution (0.1% DMF) or freshly thawed when loaded in BGs. After 24 h of incubation, the cells were harvested with accutase and washed with PBS. The cells were then resuspended in 200 µL of PBS each and 1 µL of PI solution (Sigma‐Aldrich, Taufkirchen, Germany) and 1 µL of Annexin V‐FITC (MabTag GmbH, Friesoythe, Germany) were added to each sample. These were then incubated for 20 min at rt under light‐protected conditions and afterward transferred onto ice. The percentage of apoptotic cells was determined by analyzing Annexin V positive /PI negative cells using a BD LSRFortessa flow cytometer and BD FACSDiva software (both BD Biosciences, San Jose, CA, USA). The apoptosis analysis included the evaluation of early apoptosis (Annexin V positive/PI negative) and late apoptosis/necrosis (Annexin V positive /PI positive).

### 4.12 Caspase‐3 Assay

The caspase‐3 assay was performed to investigate the induction of apoptosis by the compounds. It was performed by employing the commercially available ApoTox‐Glo Triplex Assay (Promega Corporation, Madison, USA), which specifically correlates cell viability and toxicity to apoptosis. In brief, A2780wt and A2780cis cells, respectively, were seeded at a density of 8 × 10^3^ cells per well into an opaque‐walled 96‐well plate with solid bottoms. After the cells have adhered for 24 h, complexes **1**–**4** (alone and loaded into the respective BGs strains) were added at a concentration of 25 µM and incubated for further 24 h. After incubation, 20 µL of the provided viability/toxicity reagent was added and incubated for further 30 min on an orbital shaker at 37°C. Afterwards, the emission was measured at 400_Ex_/505_Em_ for the determination of cell viability and at 485_Ex_/520_Em_ for the evaluation of the cytotoxicity. Then, 100 µL of Caspase‐Glo 3/7 reagent was added, gently shaken for 30 s and further incubated for 30 min. The caspase activation was determined via the measurement of the luminescence. Analysis was performed on the BioTek Synergy H1 (Agilent Technologies, Santa Clara, USA).

### 4.13 ATP Assay

The ATP assay was performed by employing the commercially available ATPlite Luminescence Assay System 96‐well (revvity, Massachusetts, USA). In brief, A2780wt and A2780cis cells, respectively, were seeded at a density of 8 × 10^3^ cells per well into an opaque‐walled 96‐well plate with solid bottoms. After the cells have adhered for 24 h, complexes **1**–**4** (alone and loaded into the respective BGs strains) were added at a concentration of 25 µM and incubated again for further 24 h. After incubation, 50 µL of the provided mammalian cell lysis solution was added and incubated for further 5 min on an orbital shaker at 700 rpm. Then, 50 µL of a provided substrate solution was added and again incubated for 5 min on an orbital shaker at 700 rpm. The plate was allowed to dark adapt for 10 min and then the luminescence was measured. Analysis was performed on the BioTek Synergy H1.

### 4.14 Immunofluorescent Calreticulin Staining

A2780wt or A2780cis cells were seeded at a density of 1.2 × 10^5^ on cover slips and allowed to adhere for 24 h. The cells were then treated with 25 µM of complexes **1**–**4** either in their free form or BG formulations, and oxaliplatin at 1 µM for 24 h. After fixing the cells with 4% formaldehyde for 5 min at rt and subsequently washing them three times with PBS, they were then incubated with the primary calreticulin antibody (1:200, Cell Signaling Technology; #12238) for 1 h at 37°C. Subsequently, the cells were washed again three times with PBS and they were incubated after adding the secondary antibody (Alexa‐Fluor‐conjugated anti‐rabbit, 1:200), for 45 min at rt. Subsequently, the cells were washed again three times with PBS and they were mounted on slides with mounting medium. After letting them dry overnight, visualization was performed by spinning disk confocal microscopy. Images were acquired using a 60× TIRF objective (Plan‐APOCHROMAT 60×/1.49 Oil, Nikon) mounted on an inverted microscope (Eclipse Ti2‐E; Nikon) with a spinning disc confocal unit (X‐Light V2—60 µm pinhole), a sCMOD camera (Photometrics Prime 95B), and Lumencor Spectra III LED light source (380–750 nm), controlled via the NIS‐Elements software (Nikon). Acquired images were background subtracted, individual cells were manually segmented, and fluorescence intensity for the calreticulin staining was quantified with a FIJI software [62]. Two repetitions of the experiment were performed, where at least 20 cells per condition were analyzed and the mean intensity of each sample was calculated, respectively.

## Conflicts of Interest

The authors declare no conflicts of interest.

## Supporting information

ArchPharm SupplMat InChI BGs‐0525.

Pt‐BGs‐SI 190825.

## Data Availability

The data that support the findings of this study are available in the Supporting Information of this article.
