# Supplementary material for: Loading of Dicarboxylatoplatinum(II)‐NHC Complexes in Bacterial Ghosts as an Advanced Development in Cancer Therapy
Source: Arch Pharm (Weinheim). 2025 Sep 27;358(9):e70108. doi: 10.1002/ardp.70108 (PMC12476087; doi:10.1002/ardp.70108)
Supplement: Supplementary file 1 — ArchPharm SupplMat InChI BGs‐0525. [file ARDP-358-e70108-s001.doc]

**Supplemental Material: Novel Compounds and Biological Screening Results**

**Encapsulation of dicarboxylatoplatinum(II)-NHC complexes in bacterial ghosts as an advanced development in cancer therapy**

Amelie Scherfler^1^, Klaus Wurst^2^, Stefan Schwaiger^3^, Francesco Baschieri^4^, Martin Hermann^5^, Daniel Baecker^6^, Irena Pashkunova-Martic^7,*^, Brigitte Kircher^8,9,*^, Hristo P. Varbanov^1,*^

1 Department of Pharmaceutical Chemistry, Institute of Pharmacy, Center for Molecular Biosciences Innsbruck, University of Innsbruck, Innrain 80/82, 6020 Innsbruck, Austria.

2 Department of General, Inorganic, and Theoretical Chemistry, University of Innsbruck, Innrain 80/82, 6020 Innsbruck, Austria.
3 Department of Pharmacognosy, Institute of Pharmacy, Center for Molecular Biosciences Innsbruck, University of Innsbruck, Innrain 80/82, 6020 Innsbruck, Austria.
4 Institute of Pathophysiology, Medical University of Innsbruck, Innrain 80/82, 6020 Innsbruck, Austria

5 Department of Anesthesiology & Critical Care Medicine, Medical University of Innsbruck, Anichstraße 35, 6020 Innsbruck, Austria
6 Department of Pharmaceutical and Medicinal Chemistry, Institute of Pharmacy, Freie Universität Berlin, Königin-Luise-Straße 2+4, 14195 Berlin, Germany.

7 Department of Biomedical Imaging and Image-Guided Therapy, Division of Structural and Molecular Preclinical Imaging, Medical University of Vienna and General Hospital of Vienna, Währinger Gürtel 18-20, 1090 Vienna, Austria
8 Department of Internal Medicine V, Haematology & Oncology, Immunobiology and Stem Cell Laboratory, Medical University of Innsbruck, Anichstrasse 35, 6020 Innsbruck, Austria.
9 Tyrolean Cancer Research Institute, Innrain 66, 6020 Innsbruck, Austria.

*Correspondence:

Irena Pashkunova-Martic - Department of Biomedical Imaging and Image-Guided Therapy, Division of Structural and Molecular Preclinical Imaging, Medical University of Vienna and General Hospital of Vienna, Währinger Gürtel 18-20, 1090 Vienna, Austria; orcid.org/0000-0003-0312-6461; Email: [irena.pashkunova-martic@meduniwien.ac.at](mailto:irena.pashkunova-martic@meduniwien.ac.at)

Brigitte Kircher - Department of Internal Medicine V, Haematology & Oncology, Immunobiology and Stem Cell Laboratory, Medical University Innsbruck, Anichstrasse 35, 6020 Innsbruck, Austria; Tyrolean Cancer Research Institute, Innrain 66, 6020 Innsbruck, Austria; orcid.org/0000-0003-1624-2664; Email: [brigitte.kircher@i-med.ac.at](mailto:brigitte.kircher@i-med.ac.at)

Hristo P. Varbanov - Department of Pharmaceutical Chemistry, Institute of Pharmacy, Center for Molecular Biosciences Innsbruck, University of Innsbruck, Innrain 80/82, 6020 Innsbruck, Austria; orcid.org/0000-0003-4450-7332; Email: [hristo.varbanov@uibk.ac.at](mailto:hristo.varbanov@uibk.ac.at)

| **Compound No.** | **InChI** | **Biological Activity (IC_50_)^*^** |
| --- | --- | --- |
| 1 | InChI=1S/C11H14N2.2C2H4O2.C2H6OS.Pt/c1-3-12-9-13(4-2)11-8-6-5-7-10(11)12;2*1-2(3)4;1-4(2)3;/h5-8H,3-4H2,1-2H3;2*1H3,(H,3,4);1-2H3;/q;;;;+2/p-2 | 31.13 µM (A2780wt), 36.51 µM (A2780cis) |
| 2 | InChI=1S/2C11H14N2.2C2H4O2.Pt/c2*1-3-12-9-13(4-2)11-8-6-5-7-10(11)12;2*1-2(3)4;/h2*5-8H,3-4H2,1-2H3;2*1H3,(H,3,4);/q;;;;+2/p-2 | 40.78 µM (A2780wt), 39.28 µM (A2780cis) |
| 3 | InChI=1S/C11H14N2.2C2H2Cl2O2.C2H7OS.Pt/c1-3-12-9-13(4-2)11-8-6-5-7-10(11)12;2*3-1(4)2(5)6;1-4(2)3;/h5-8H,3-4H2,1-2H3;2*1H,(H,5,6);4H,1-2H3;/q;;;;+2/p-2 | 58.87 µM (A2780wt), 83.12 µM (A2780cis) |
| 4 | InChI=1S/2C11H14N2.2C2H2Cl2O2.Pt/c2*1-3-12-9-13(4-2)11-8-6-5-7-10(11)12;2*3-1(4)2(5)6;/h2*5-8H,3-4H2,1-2H3;2*1H,(H,5,6);/q;;;;+2/p-2 | 37.85 µM (A2780wt), 34.86 µM (A2780cis) |

^*^ determined with the MTT assay (72 h exposure)
