# Supplementary material for: Loading of Dicarboxylatoplatinum(II)‐NHC Complexes in Bacterial Ghosts as an Advanced Development in Cancer Therapy
Source: Arch Pharm (Weinheim). 2025 Sep 27;358(9):e70108. doi: 10.1002/ardp.70108 (PMC12476087; doi:10.1002/ardp.70108)
Supplement: Supplementary file 2 — Pt‐BGs‐SI 190825. [file ARDP-358-e70108-s002.docx]

Supporting Information:

**Loading of dicarboxylatoplatinum(II)-NHC complexes in bacterial ghosts as an advanced development in cancer therapy**

Amelie Scherfler^1^, Klaus Wurst^2^, Stefan Schwaiger^3^, Francesco Baschieri^4^, Martin Hermann^5^, Daniel Baecker^6^, Irena Pashkunova-Martic^7,*^, Brigitte Kircher^8,9,*^, Hristo P. Varbanov^1,*^

^1^ Department of Pharmaceutical Chemistry, Institute of Pharmacy, Center for Molecular Biosciences Innsbruck, University of Innsbruck, Innrain 80/82, 6020 Innsbruck, Austria.

^2^ Department of General, Inorganic, and Theoretical Chemistry, University of Innsbruck, Innrain 80/82, 6020 Innsbruck, Austria.
^3^ Department of Pharmacognosy, Institute of Pharmacy, Center for Molecular Biosciences Innsbruck, University of Innsbruck, Innrain 80/82, 6020 Innsbruck, Austria.
^4^ Institute of Pathophysiology, Medical University Innsbruck, Innrain 80/82, 6020 Innsbruck, Austria

^5^ Department of Anesthesiology & Critical Care Medicine, Medical University Innsbruck, Anichstraße 35, 6020 Innsbruck, Austria
^6^ Department of Pharmaceutical and Medicinal Chemistry, Institute of Pharmacy, Freie Universität Berlin, Königin-Luise-Straße 2+4, 14195 Berlin, Germany.

^7^ Department of Biomedical Imaging and Image-Guided Therapy, Division of Structural and Molecular Preclinical Imaging, Medical University of Vienna and General Hospital of Vienna, Währinger Gürtel 18-20, 1090 Vienna, Austria
^8^ Department of Internal Medicine V, Haematology & Oncology, Immunobiology and Stem Cell Laboratory, Medical University Innsbruck, Anichstrasse 35, 6020 Innsbruck, Austria.
^9^ Tyrolean Cancer Research Institute, Innrain 66, 6020 Innsbruck, Austria.

**Table of contents:**

[1. NMR spectra of complexes **1**-**4** S2](#_Toc206013052)

[2. ESI-HRMS spectra of complexes **1**-**4** S7](#_Toc206013053)

[3. Crystallographic data of complexes **1**-**4** S9](#_Toc206013054)

[4. Time dependent ^1^H NMR spectra of complexes **1**-**4** S13](#_Toc206013055)

[5. RP-HPLC experiments S17](#_Toc206013056)

[6. HPLC-MS stability of **1** and **2** in RPMI 1640 S19](#_Toc206013057)

[7. Additional biological data S21](#_Toc206013058)

[7.1. Comparison of cytotoxicity of complexes **1**-**4** and carboplatin across the tested cell lines S21](#_Toc206013059)

[7.2. Concentration-effect curves of complexes **1**-**4** and carboplatin on A2780wt and A2780cis cells S22](#_Toc206013060)

[7.3. Additional image of comet assay S22](#_Toc206013061)

[7.4. Induction of apoptosis and necrosis S23](#_Toc206013062)

[7.5. Caspase-3 induction S23](#_Toc206013063)

[7.6. Representative images of surface calreticulin exposure S24](#_Toc206013064)

[8. Time-temperature program for the AAS measurement S25](#_Toc206013065)

# NMR spectra of complexes **1**-**4**

- 1. ^1^H and ^13^C NMR spectra of complexes **1**-**4**


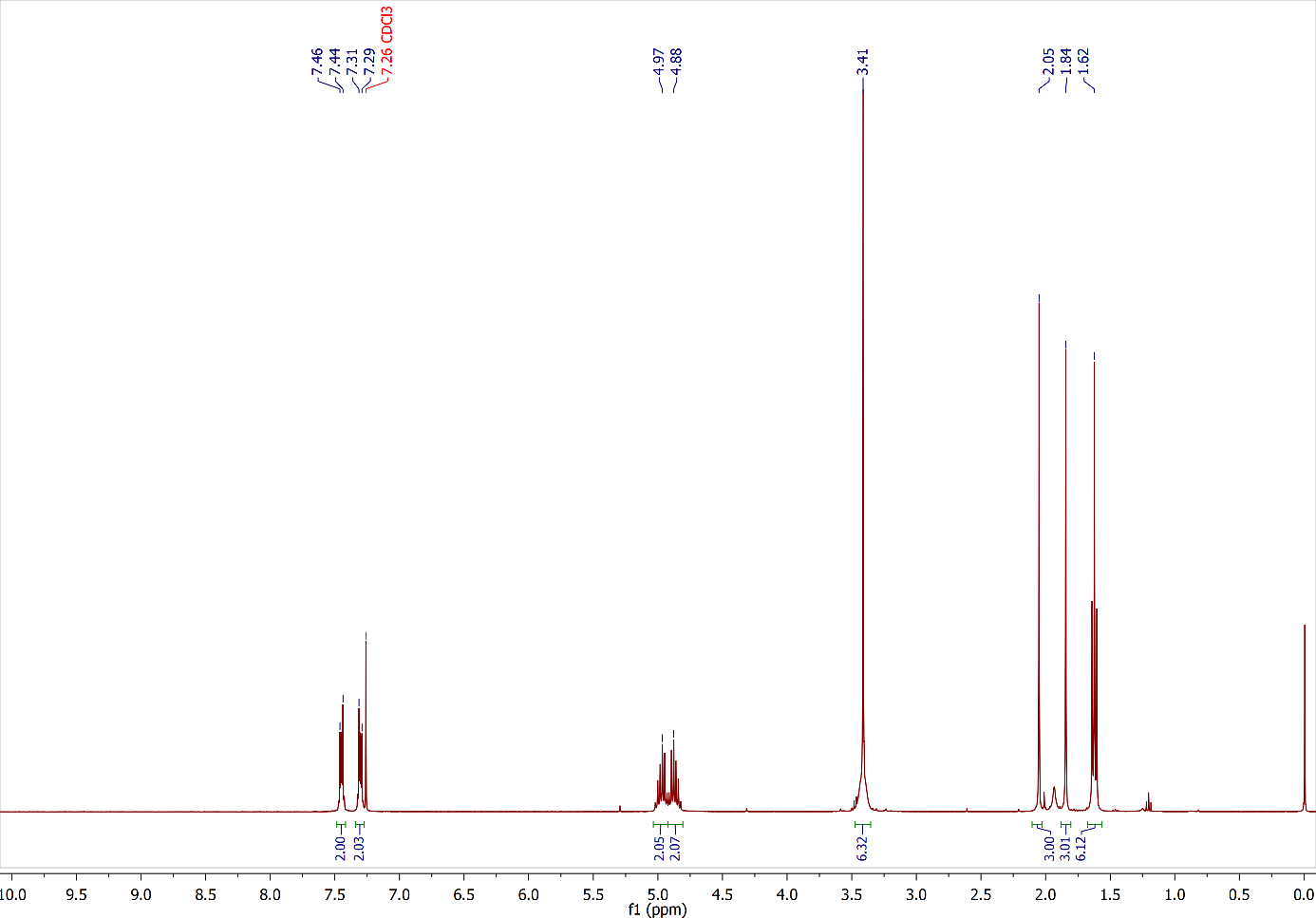


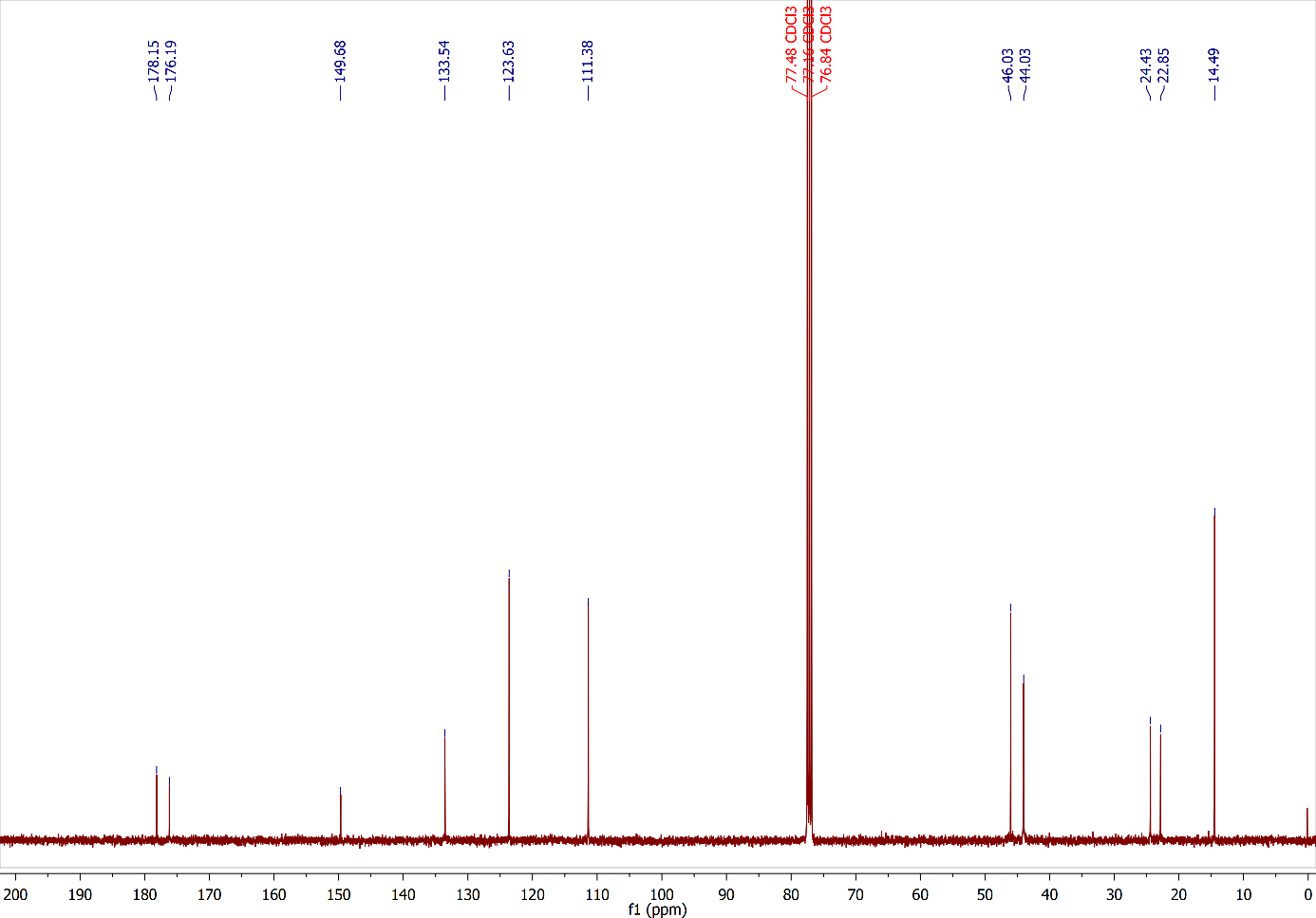


**Figure S1.** ^1^H (top) and ^13^C (bottom) NMR spectra of complex **1** recorded in CDCl_3_ (+0.03% TMS) at ambient temperature.


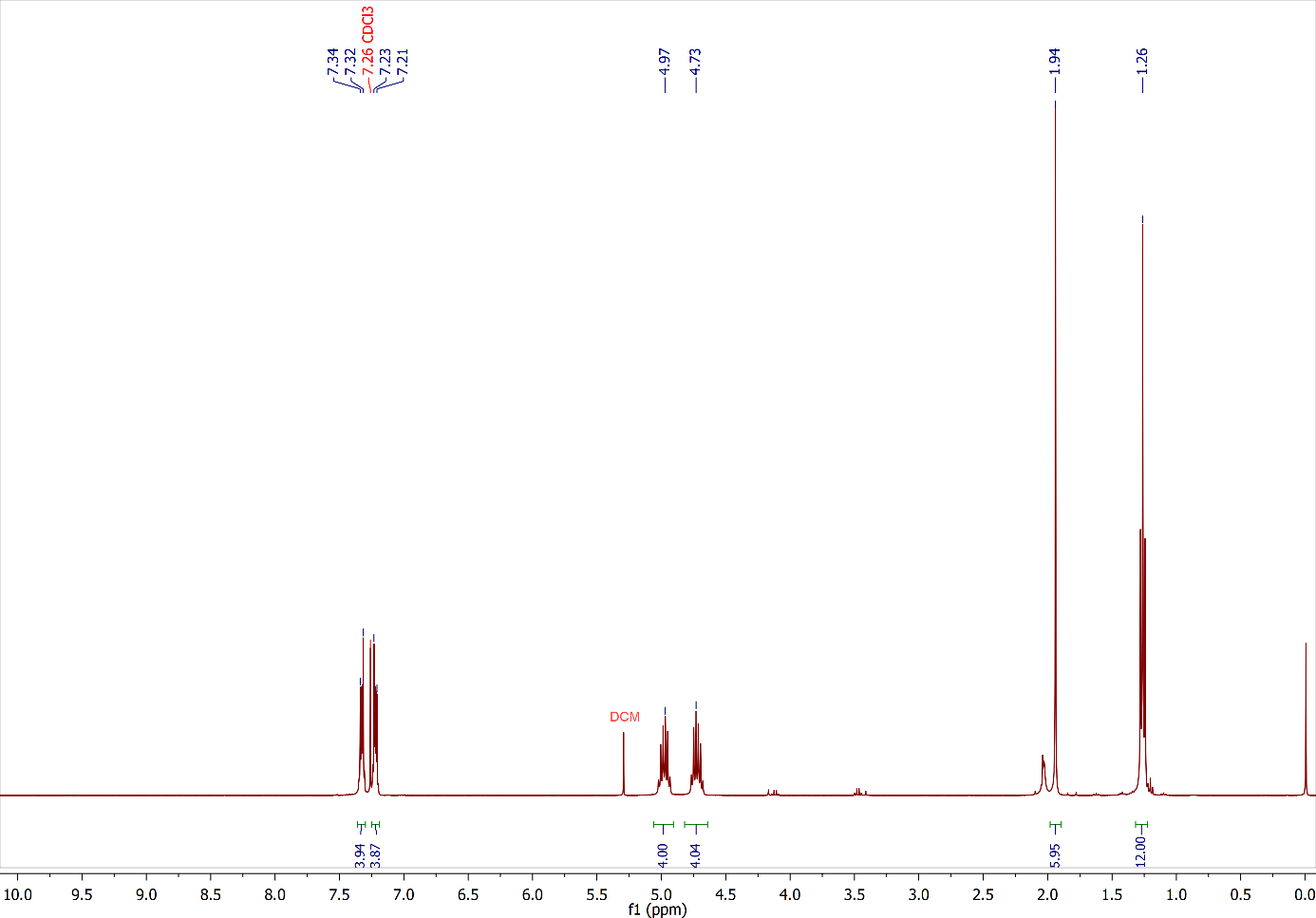


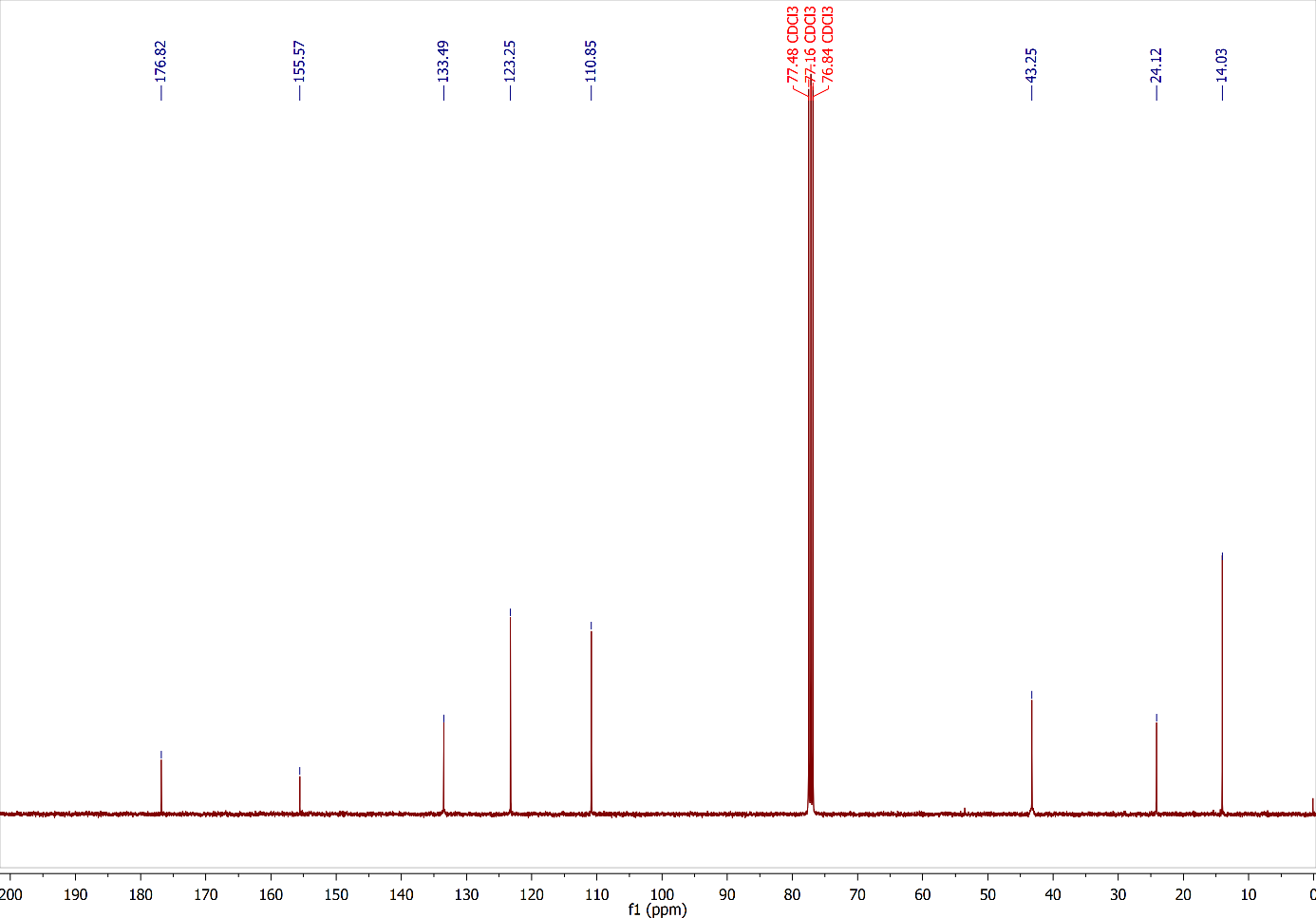


**Figure S2.** ^1^H (top) and ^13^C (bottom) NMR spectra of complex **2** recorded in CDCl_3_ (+0.03% TMS) at ambient temperature.


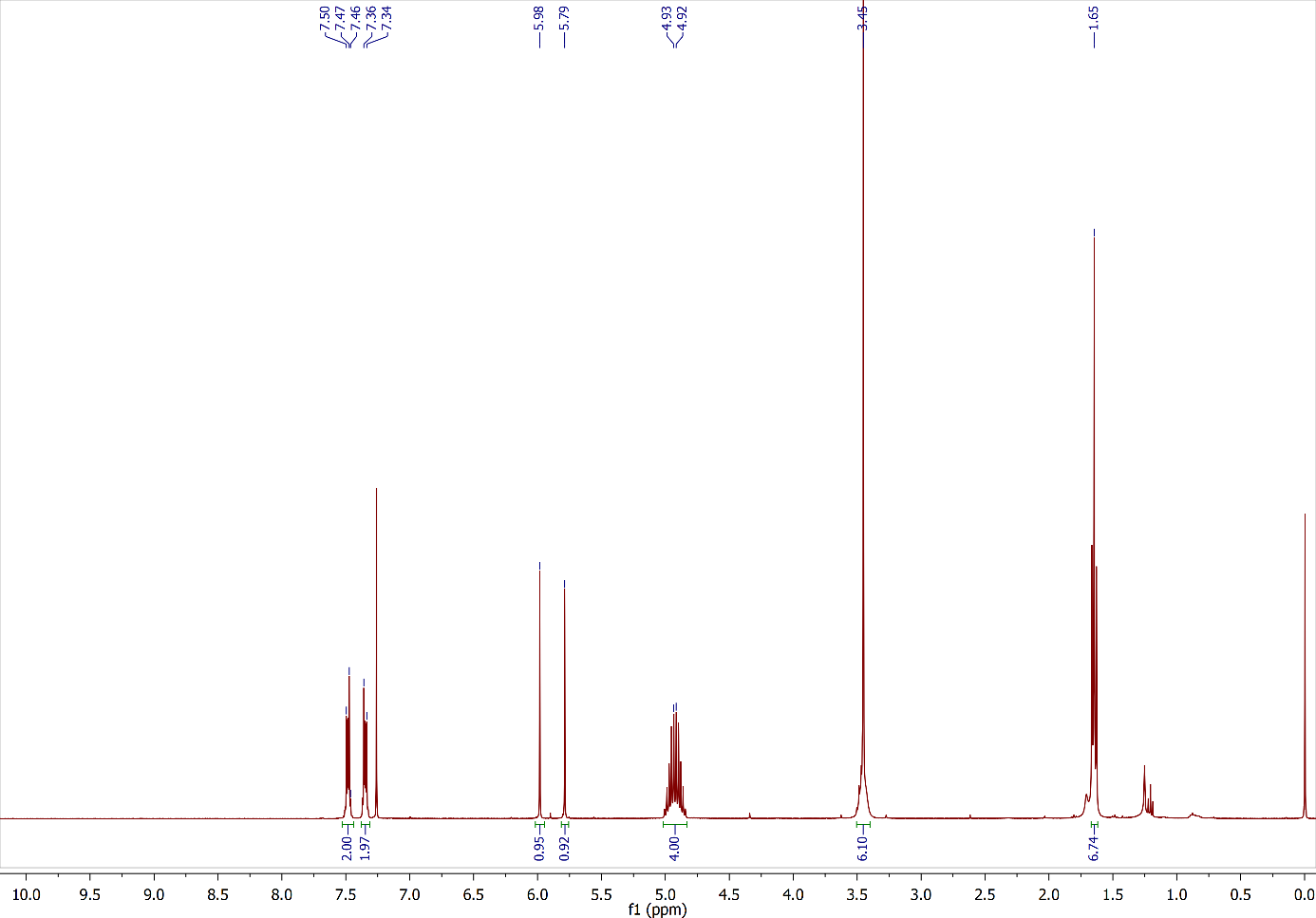


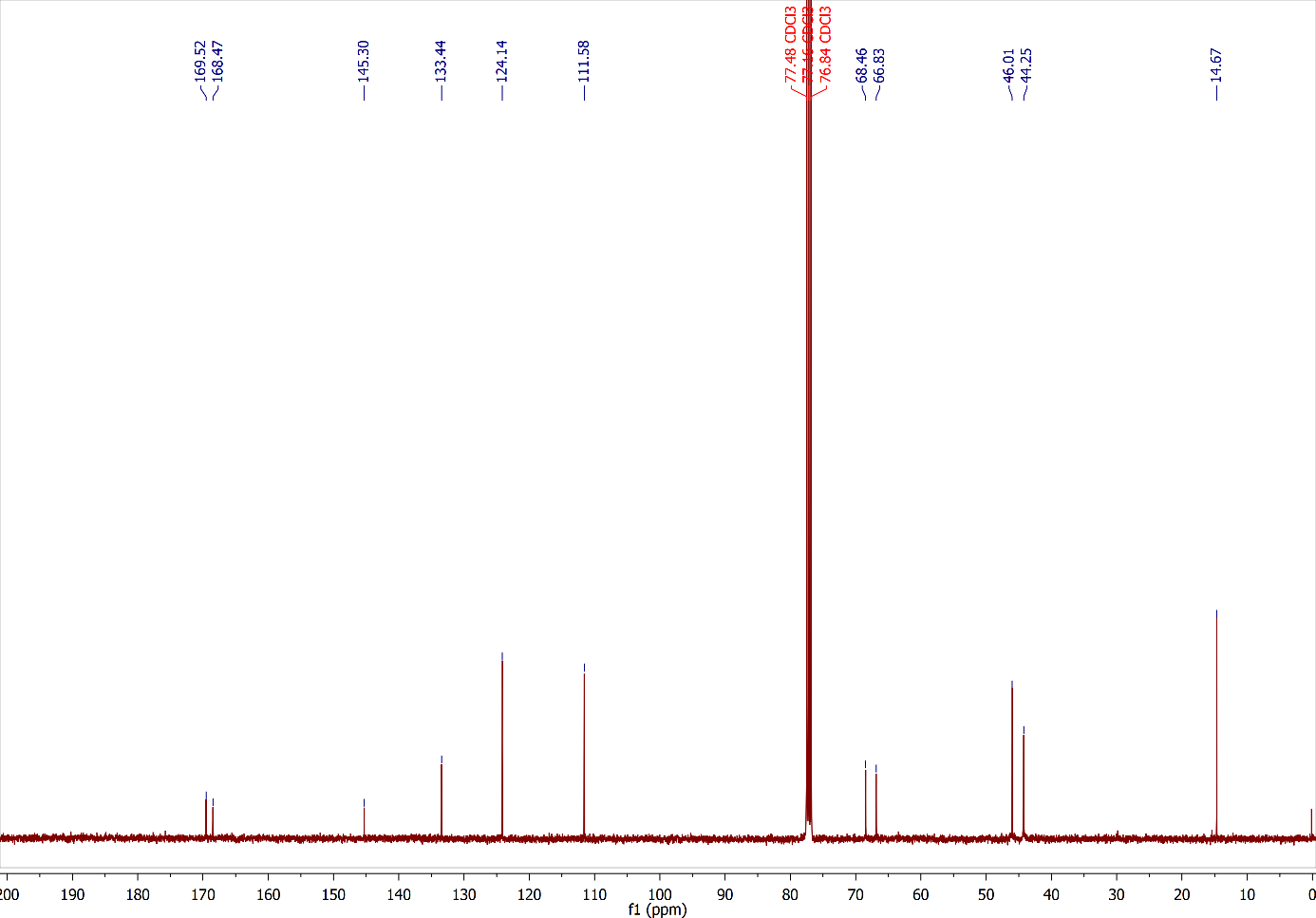


**Figure S3.** ^1^H (top) and ^13^C (bottom) NMR spectra of complex **3** recorded in CDCl_3_ (+0.03% TMS) at ambient temperature.


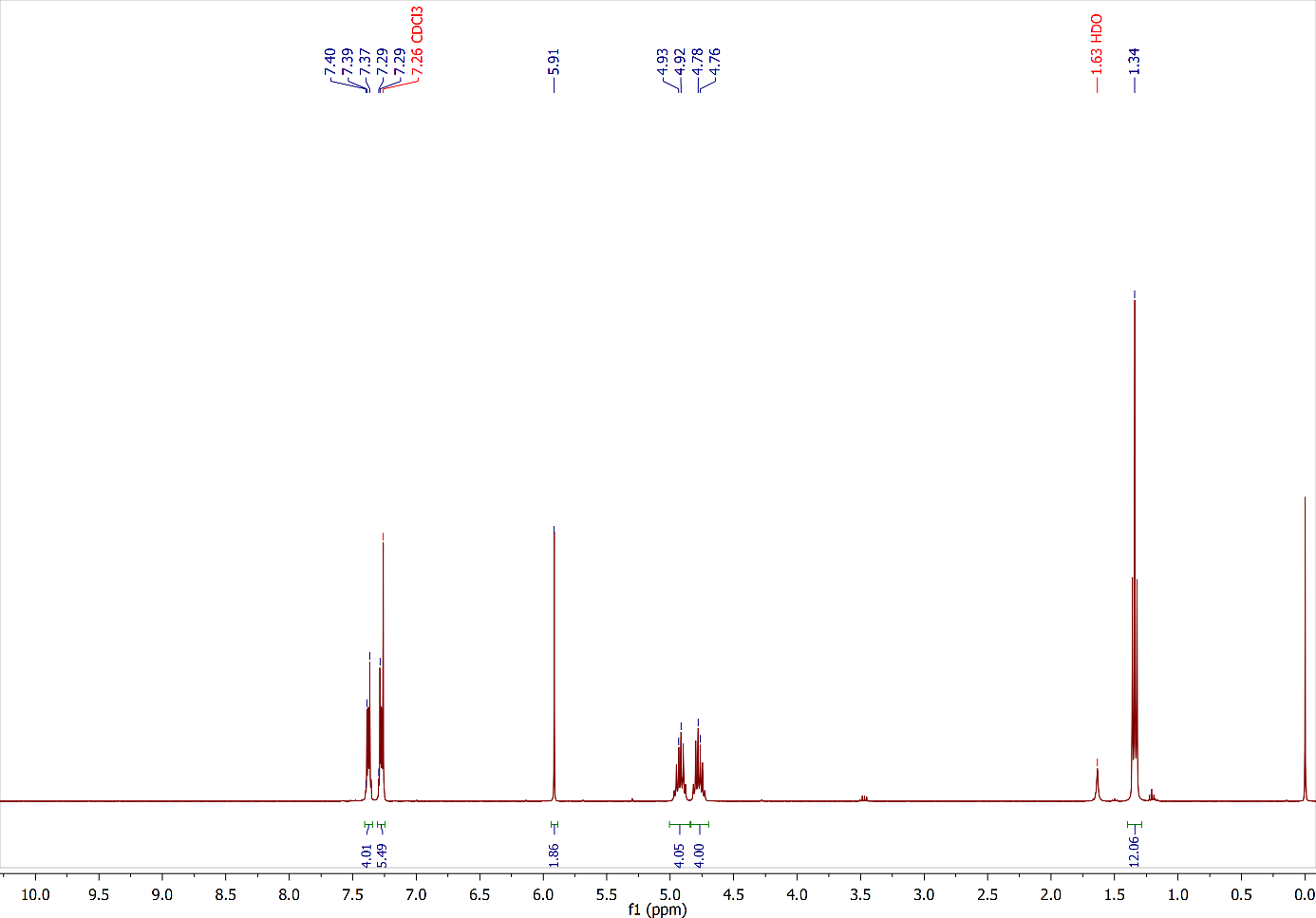


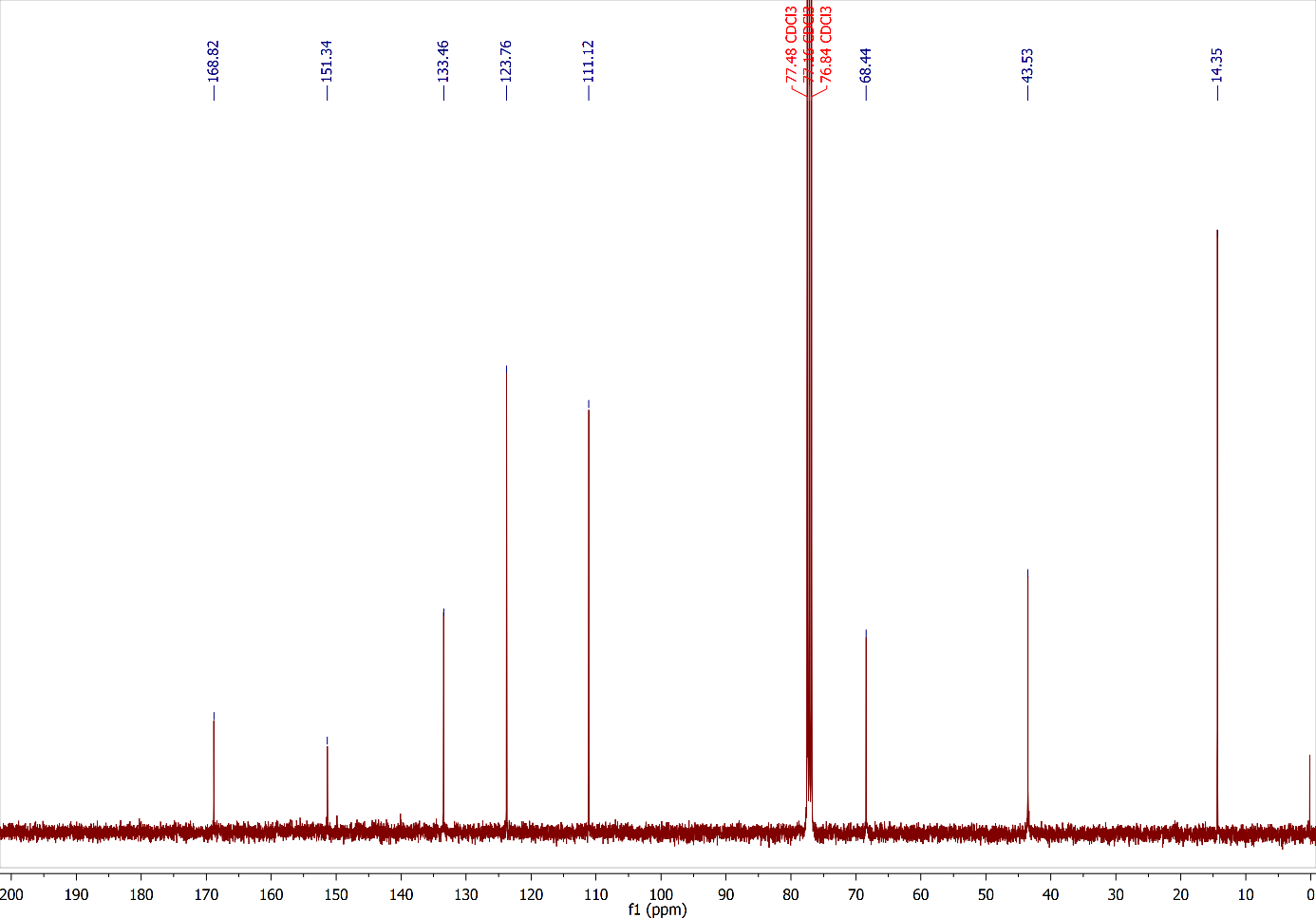


**Figure S4.** ^1^H (top) and ^13^C (bottom) NMR spectra of complex **4** recorded in CDCl_3_ (+0.03% TMS) at ambient temperature.

- 1. ^195^Pt NMR spectra of complexes **1**-**4**


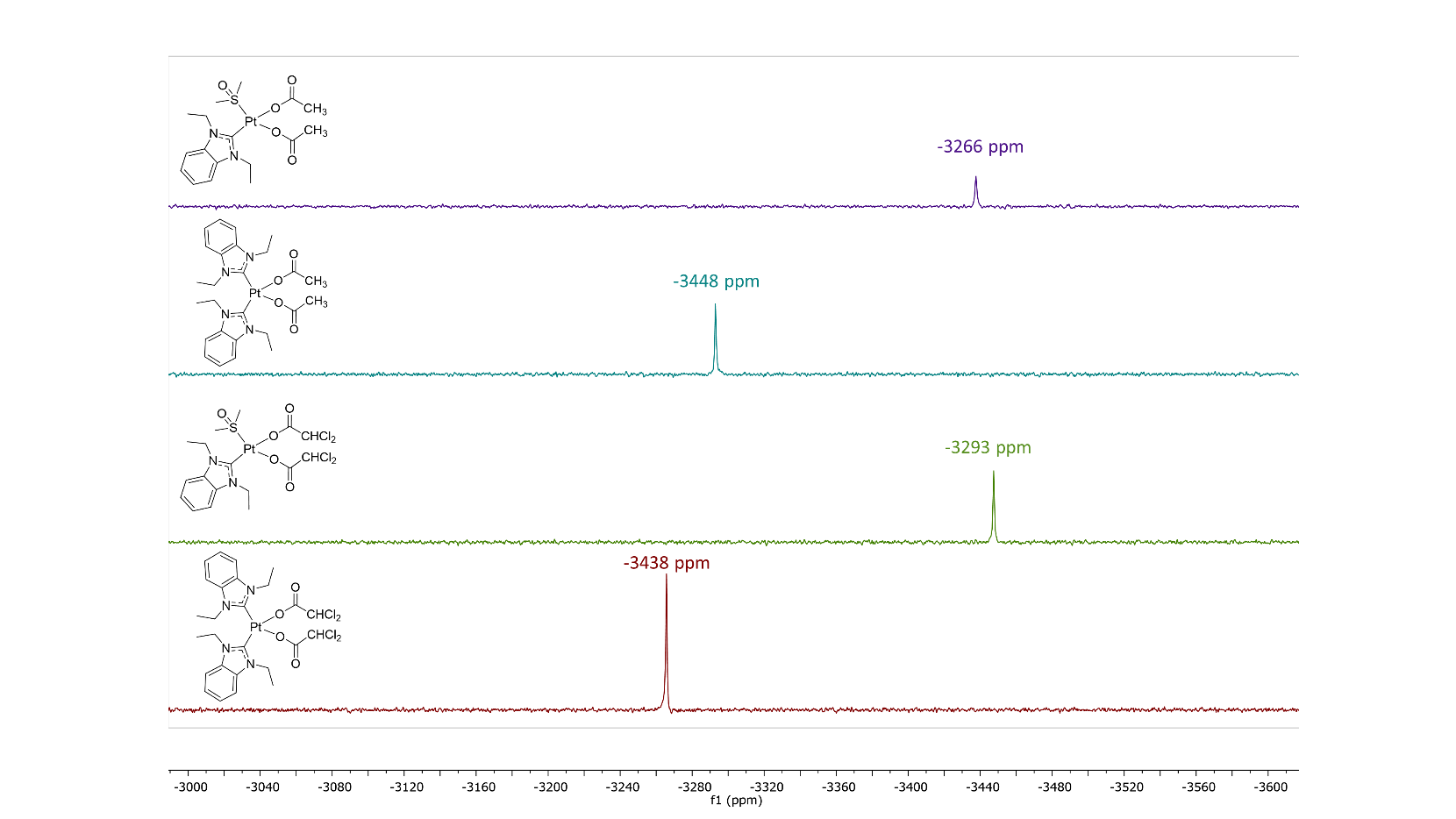


**Figure S5**. ^195^Pt NMR spectra of complexes **1**-**4** (top to bottom) recorded in CDCl_3_. The spectra were processed with a line broadening factor of 30 Hz.

# ESI-HRMS spectra of complexes **1**-**4**


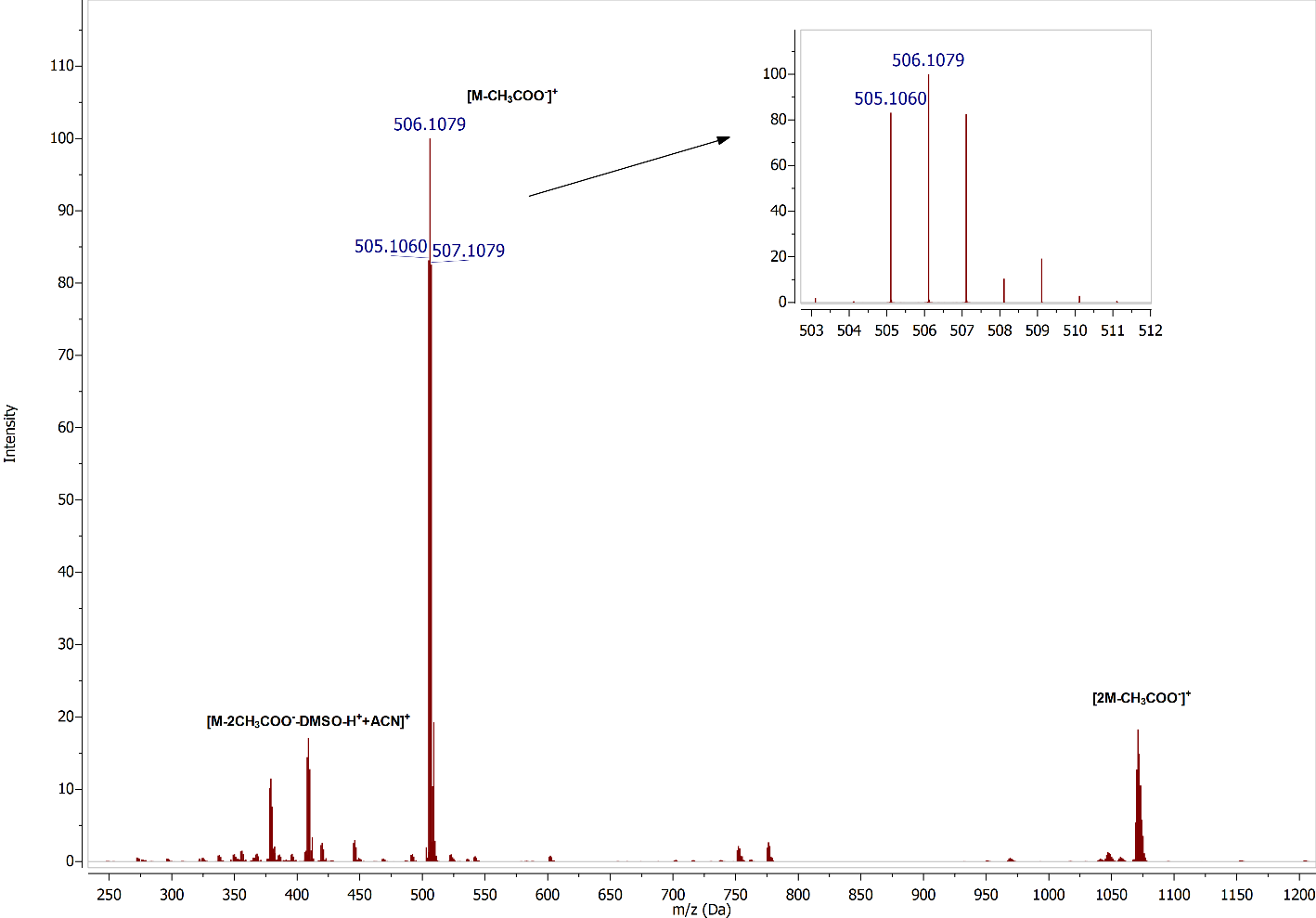


**Figure S6.** ESI-HRMS (+) spectrum of complex **1**


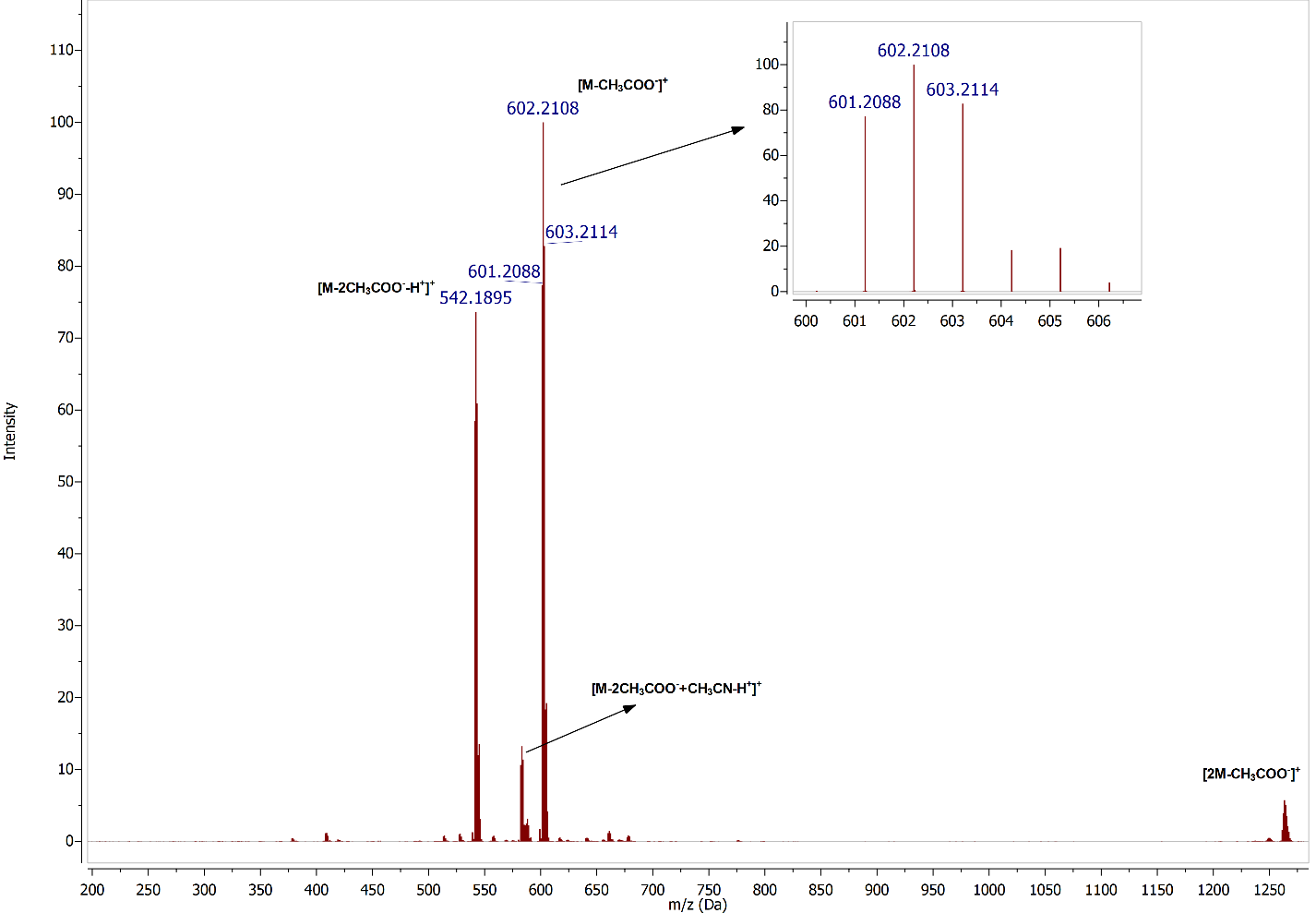


**Figure S7.** ESI-HRMS (+) spectrum of complex **2**


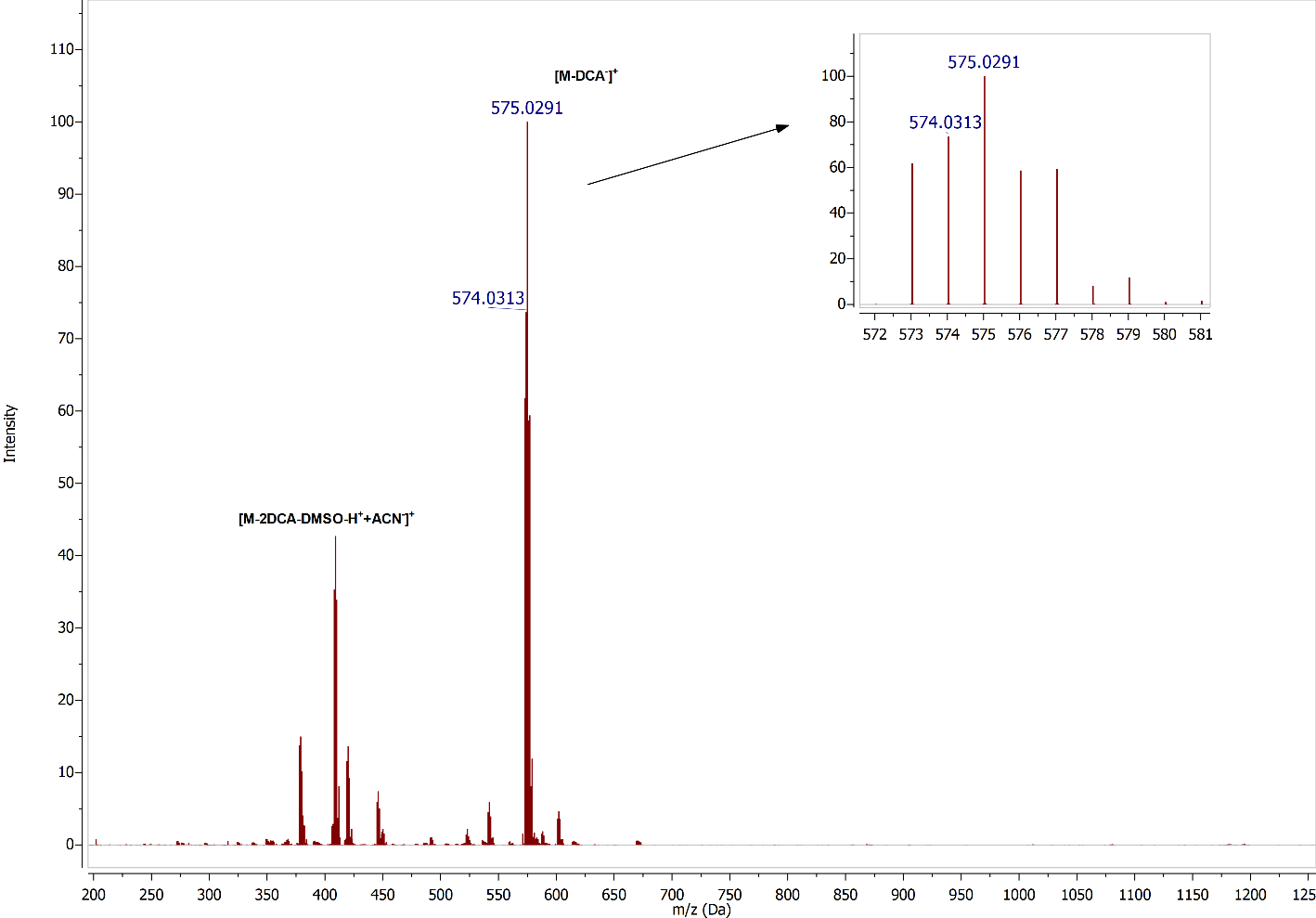


**Figure S8.** ESI-HRMS (+) spectrum of complex **3**


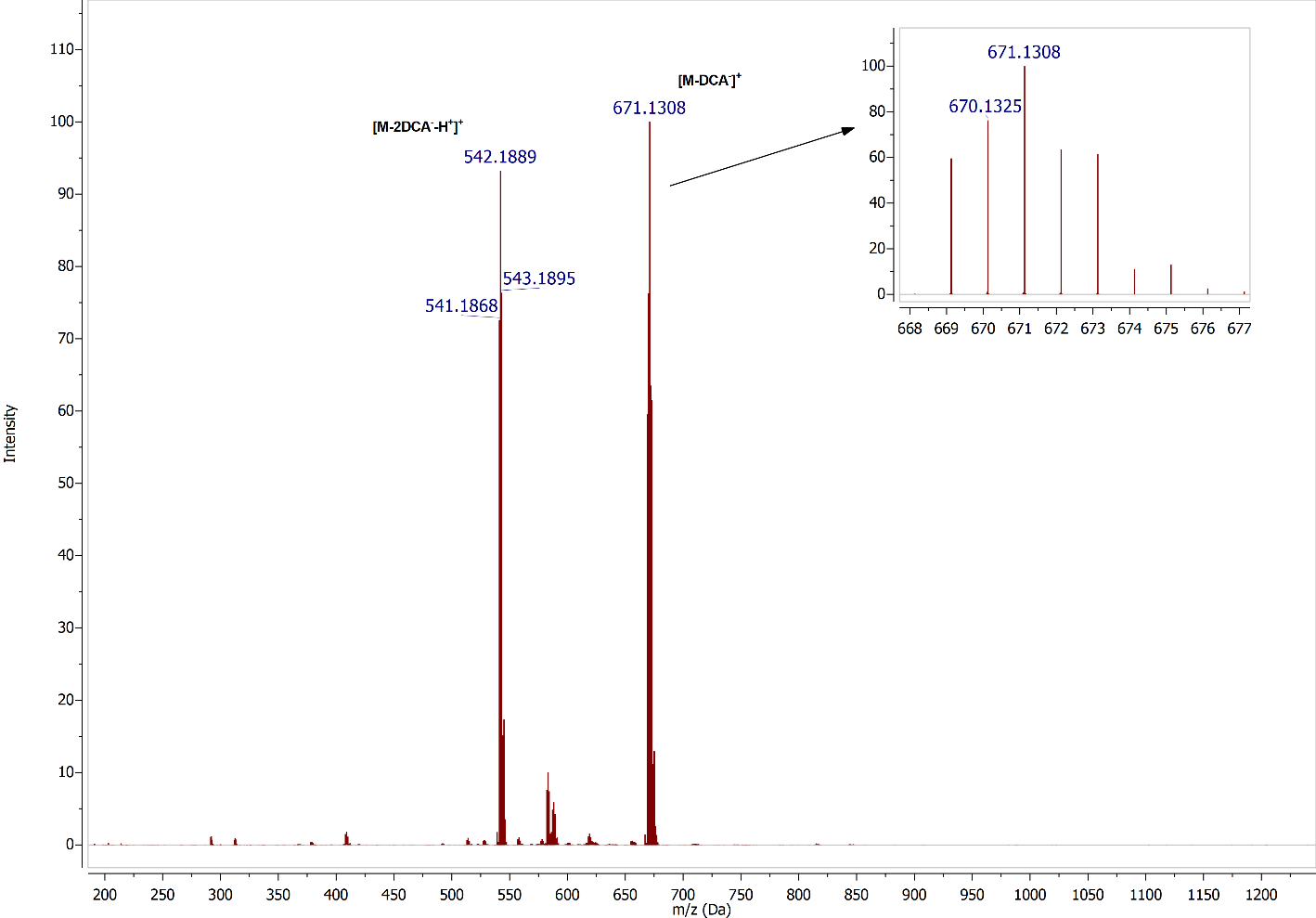


**Figure S9.** ESI-HRMS (+) spectrum of complex **4**

# Crystallographic data of complexes **1**-**4**

**Table S1.** Crystal data and structure refinement for complex **1**.

CCDC number 2449410

Empirical formula C_17_H_26_N_2_O_5_PtS

Formula weight 565.55

Temperature 173.00 K

Wavelength 0.71073 Å

Crystal system Monoclinic

Space group P2_1_/n (no. 14)

Unit cell dimensions a = 11.0987(3) Å α = 90°

b = 11.5582(3) Å β = 102.6110(10)°

c = 15.9135(4) Å γ = 90°

Volume 1992.15(9) Å3

Z 4

Density (calculated) 1.886 mg/m3

Absorption coefficient 7.177 mm-1

F(000) 1104

Crystal size 0.18 x 0.08 x 0.06 mm3

Theta range for data collection 2.517 to 28.745°.

Index ranges -15<=h<=15, -15<=k<=15, -21<=l<=21

Reflections collected 51702

Independent reflections 5147 [R(int) = 0.0334]

Completeness to theta = 25.242° 99.6 %

Absorption correction Semi-empirical from equivalents

Max. and min. transmission 0.6037 and 0.3215

Refinement method Full-matrix least-squares on F2

Data / restraints / parameters 5147 / 0 / 241

Goodness-of-fit on F2 1.049

Final R indices [I>2sigma(I)] R1 = 0.0137, wR_2_ = 0.0310

R indices (all data) R1 = 0.0146, wR_2_ = 0.0314

Extinction coefficient n/a

Largest diff. peak and hole 0.956 and -0.574 e.Å-3

**Table S2**. Crystal data and structure refinement for complex **2**.

CCDC number 2449411

Empirical formula C_26_H_34_N_4_O_4_Pt x 2 CHCl_3_

Formula weight 900.40

Temperature 153.00 K

Wavelength 0.71073 Å

Crystal system Triclinic

Space group P-1 (no. 2)

Unit cell dimensions a = 8.5951(4) Å α = 94.142(2)°

b = 10.4640(5) Å β = 101.299(2)°

c = 21.1072(10) Å γ = 108.454(2)°

Volume 1747.36(14) Å3

Z 2

Density (calculated) 1.711 Mg/m3

Absorption coefficient 4.512 mm-1

F(000) 888

Crystal size 0.12 x 0.06 x 0.03 mm3

Theta range for data collection 2.166 to 27.500°.

Index ranges -11<=h<=11, -13<=k<=13, -27<=l<=27

Reflections collected 64758

Independent reflections 8035 [R(int) = 0.0316]

Completeness to theta = 25.242° 99.9 %

Absorption correction Semi-empirical from equivalents

Max. and min. transmission 0.7246 and 0.6102

Refinement method Full-matrix least-squares on F2

Data / restraints / parameters 8035 / 0 / 412

Goodness-of-fit on F2 1.073

Final R indices [I>2sigma(I)] R1 = 0.0176, wR_2_ = 0.0420

R indices (all data) R1 = 0.0186, wR_2_ = 0.0424

Extinction coefficient n/a

Largest diff. peak and hole 1.400 and -0.976 e.Å-3

**Table S3**. Crystal data and structure refinement for complex **3**.

CCDC number 2449412

Empirical formula C_17_H_22_Cl_4_N_2_O_5_PtS

Formula weight 703.31

Temperature 293.15 K

Wavelength 0.71073 Å

Crystal system Monoclinic

Space group P2_1_/c (no. 14)

Unit cell dimensions a = 9.6605(10) Å α = 90°

b = 9.5086(8) Å β = 94.276(4)°.

c = 26.410(3) Å γ = 90°

Volume 2419.2(4) Å3

Z 4

Density (calculated) 1.931 mg/m3

Absorption coefficient 6.358 mm-1

F(000) 1360

Crystal size 0.16 x 0.08 x 0.015 mm3

Theta range for data collection 2.114 to 28.301°.

Index ranges -12<=h<=12, -12<=k<=12, -35<=l<=35

Reflections collected 77288

Independent reflections 6004 [R(int) = 0.0551]

Completeness to theta = 25.242° 100.0 %

Absorption correction Semi-empirical from equivalents

Max. and min. transmission 0.8251 and 0.6649

Refinement method Full-matrix least-squares on F2

Data / restraints / parameters 6004 / 0 / 275

Goodness-of-fit on F2 1.048

Final R indices [I>2sigma(I)] R1 = 0.0274, wR_2_ = 0.0684

R indices (all data) R1 = 0.0333, wR_2_ = 0.0718

Extinction coefficient n/a

Largest diff. peak and hole 1.370 and -0.981 e.Å-3

**Table S4**. Crystal data and structure refinement for complex **4**.

CCDC number 2449413

Empirical formula C_26_H_30_Cl_4_N_4_O_4_Pt x CHCl_3_

Formula weight 918.80

Temperature 298.15 K

Wavelength 0.71073 Å

Crystal system Triclinic

Space group P-1 (no. 2)

Unit cell dimensions a = 9.7038(4) Å α = 79.265(2)°

b = 11.6161(5) Å β = 86.403(2)°

c = 16.3807(8) Å γ = 73.292(2)°

Volume 1737.45(14) Å3

Z 2

Density (calculated) 1.756 Mg/m3

Absorption coefficient 4.614 mm-1

F(000) 900

Crystal size 0.22 x 0.21 x 0.18 mm3

Theta range for data collection 2.057 to 26.999°.

Index ranges -12<=h<=12, -14<=k<=14, -20<=l<=20

Reflections collected 34626

Independent reflections 7561 [R(int) = 0.0450]

Completeness to theta = 25.242° 99.7 %

Absorption correction Semi-empirical from equivalents

Max. and min. transmission 0.5947 and 0.3467

Refinement method Full-matrix least-squares on F2

Data / restraints / parameters 7561 / 0 / 446

Goodness-of-fit on F2 1.050

Final R indices [I>2sigma(I)] R1 = 0.0281, wR_2_ = 0.0737

R indices (all data) R1 = 0.0319, wR_2_ = 0.0761

Extinction coefficient n/a

Largest diff. peak and hole 0.977 and -0.808 e.Å-3

# Time dependent ^1^H NMR spectra of complexes **1**-**4**


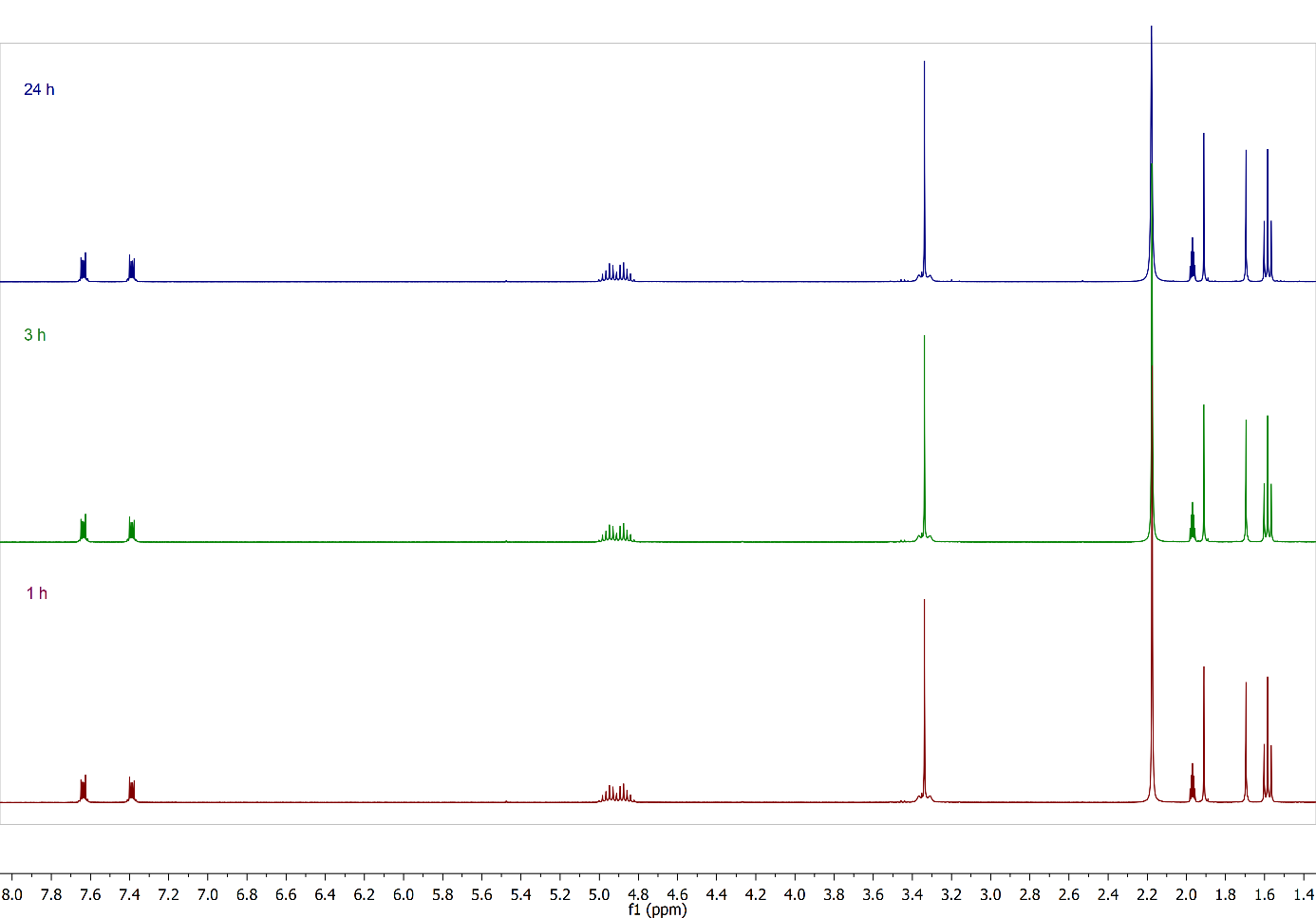


**Figure S10.** ^1^H NMR spectra of complex **1** in CD_3_CN measured after 1 h, 3 h, and 24 h of incubation at ambient temperature (from bottom to top).


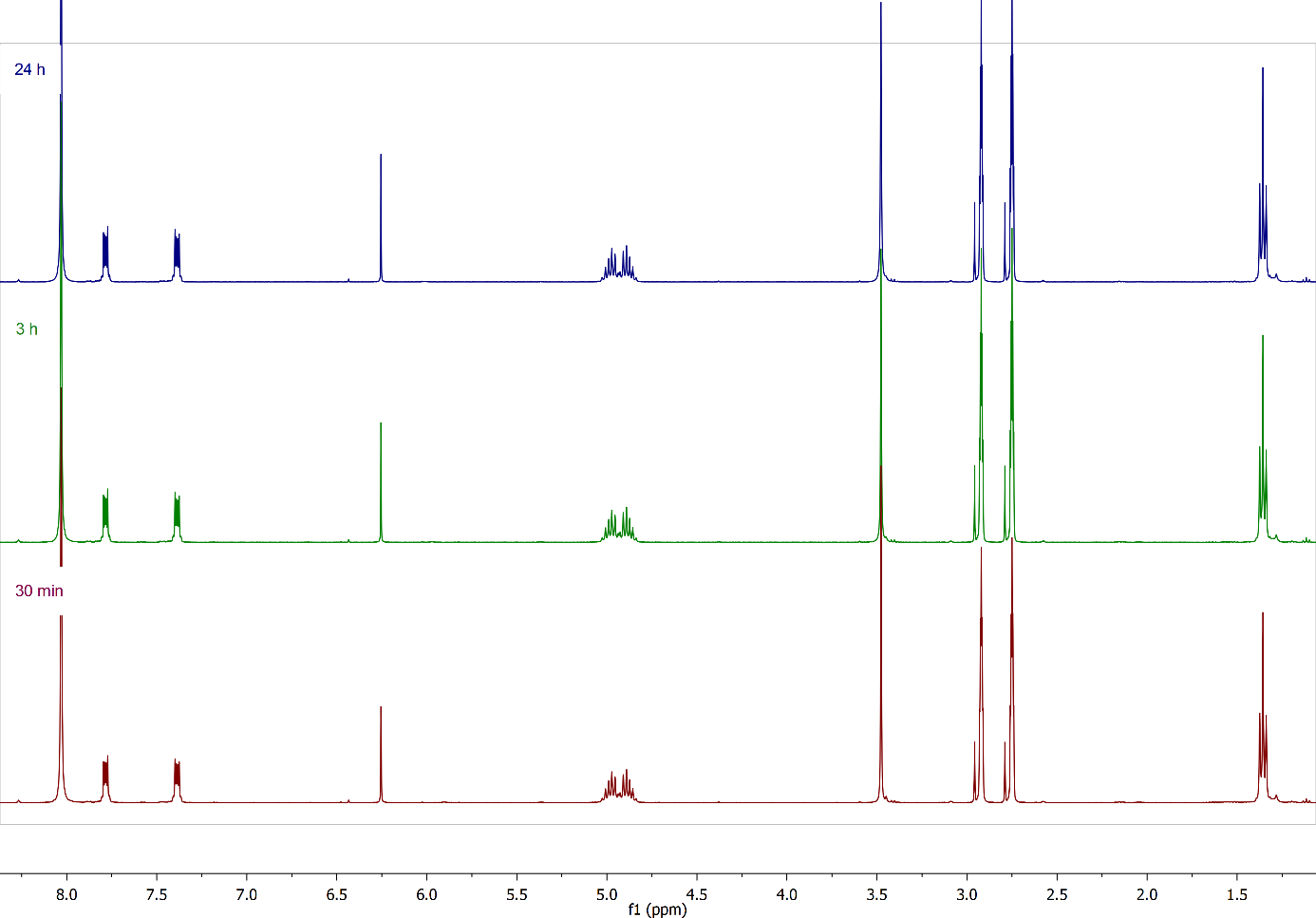


**Figure S11.** ^1^H NMR spectra of complex **4** in DMF-d_7_ measured after 0.5 h, 3 h, and 24 h of incubation at ambient temperature (from bottom to top).


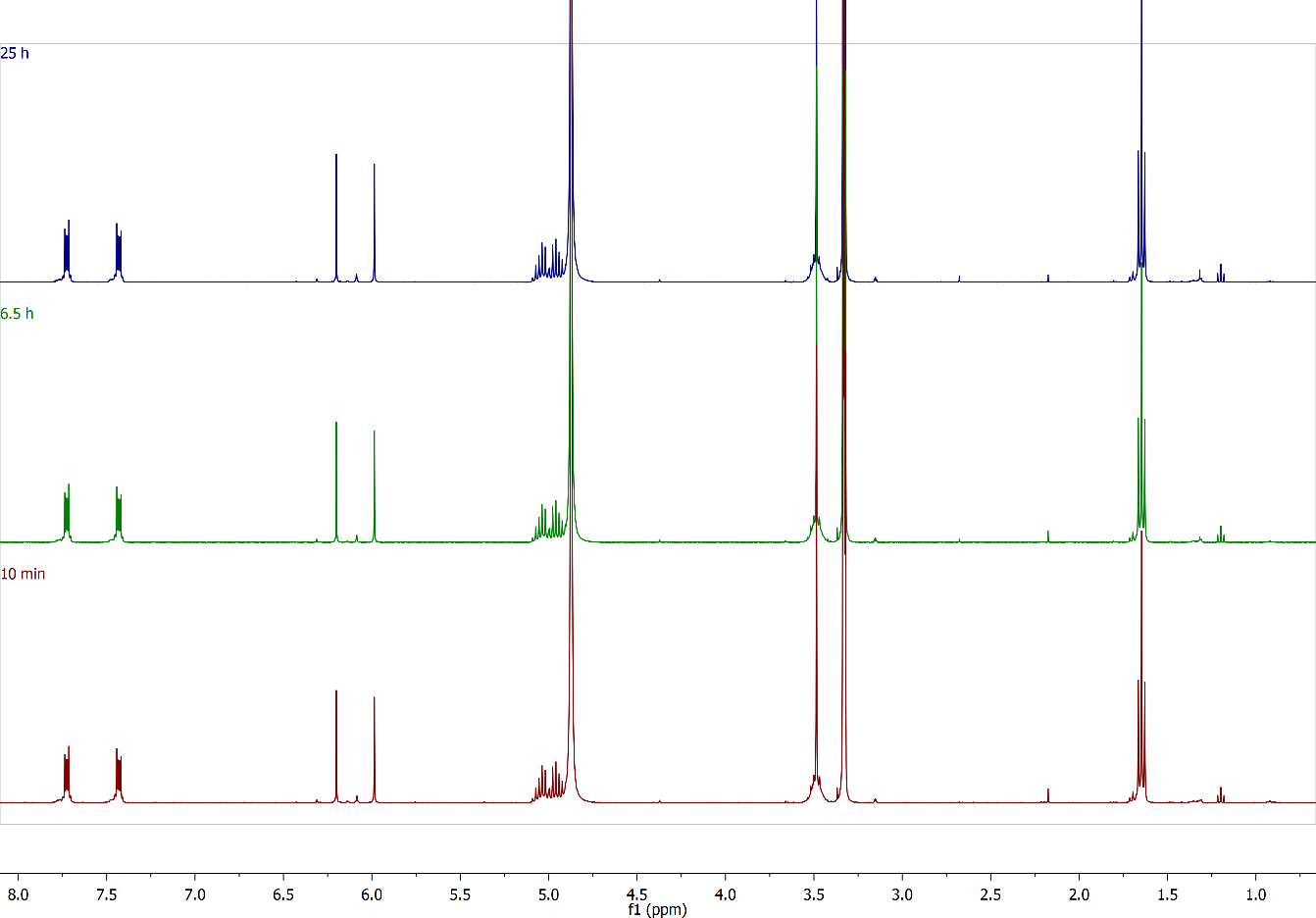


**Figure S12.** ^1^H NMR spectra of complex **3** in CD_3_OD measured after 0.5 h, 6.5 h and 25 h of incubation at ambient temperature (from bottom to top).


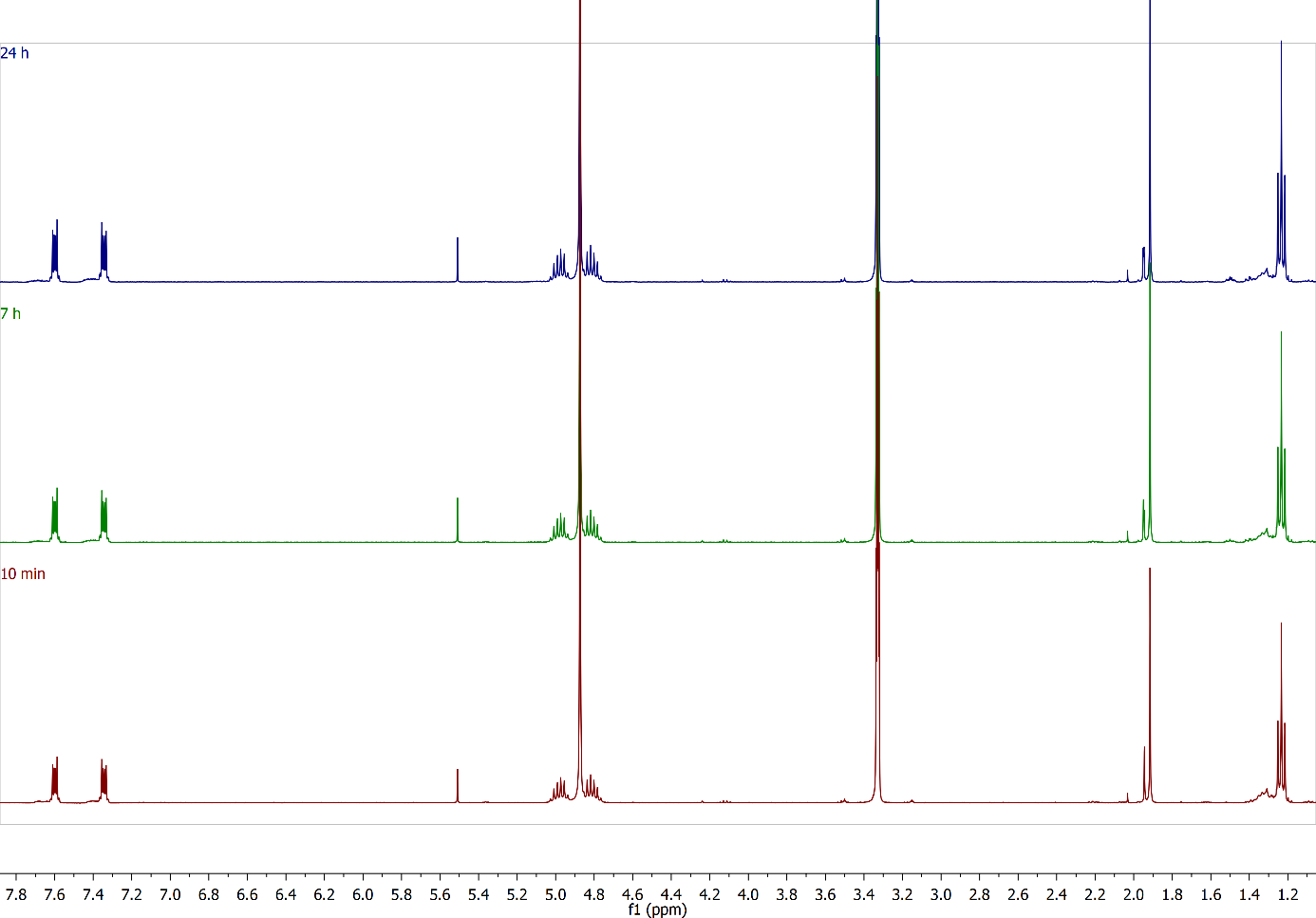


**Figure S13.** ^1^H NMR spectra of complex **2** in CD_3_OD measured after 0.5 h, 6.5 h, and 25 h of incubation at ambient temperature (from bottom to top).


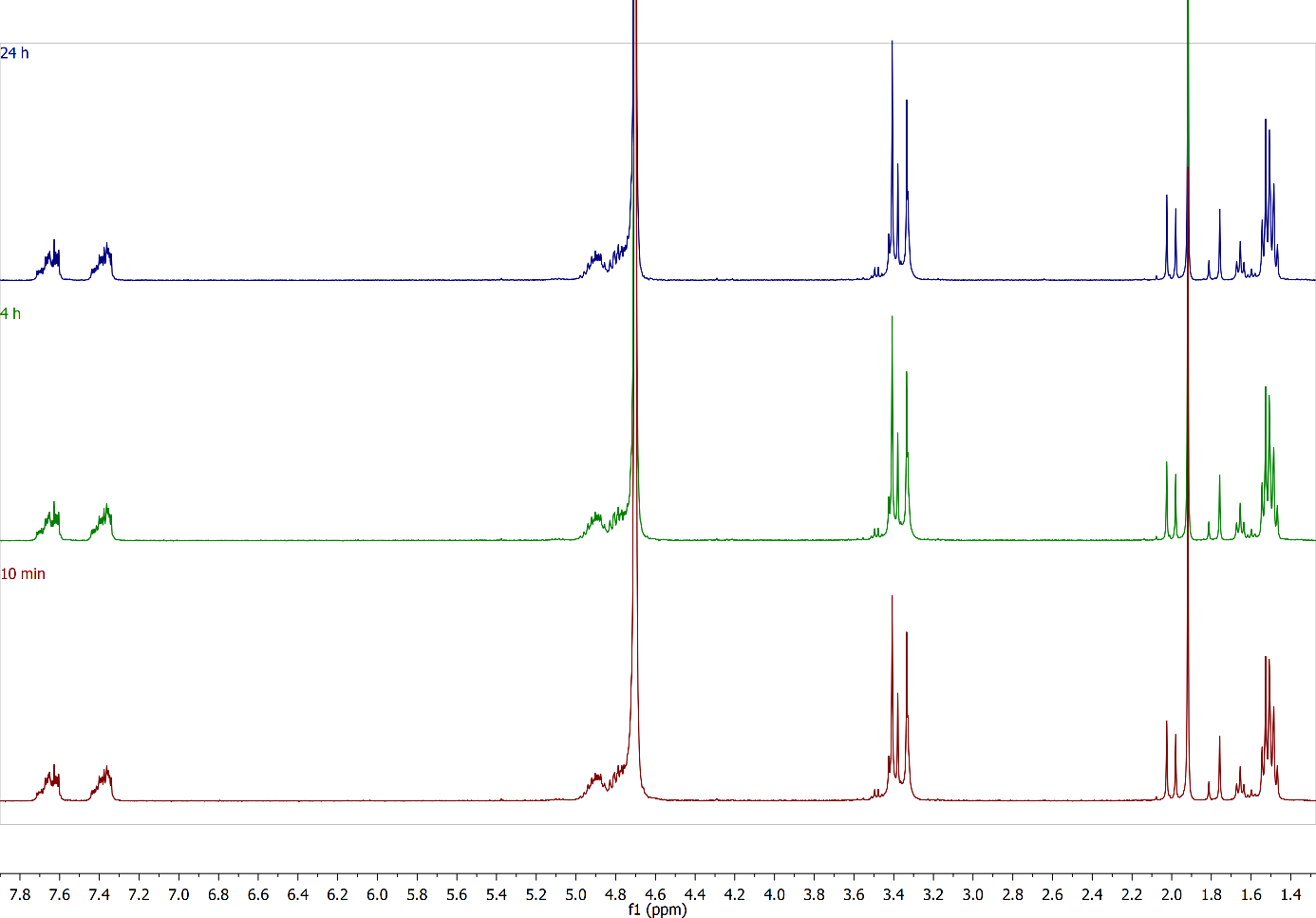


**Figure S14.** ^1^H NMR spectra of complex **1** in D_2_O measured after 10 min, 4 h, and 24 h of incubation at ambient temperature (from bottom to top).

**
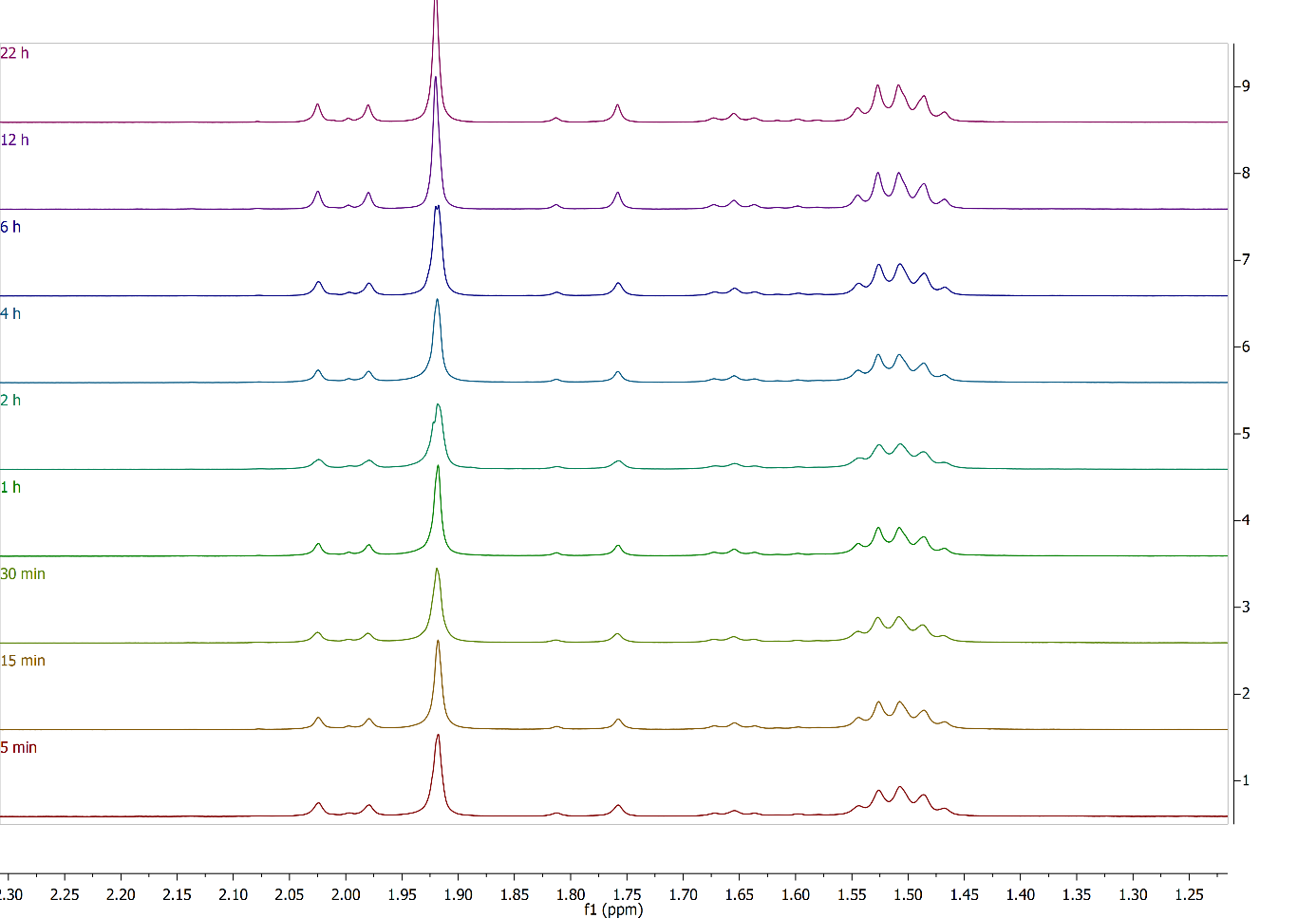
**

**Figure S15.** Time-dependent change of the appearance of the acetate signals in the ^1^H NMR spectra of complex **1** in D_2_O.


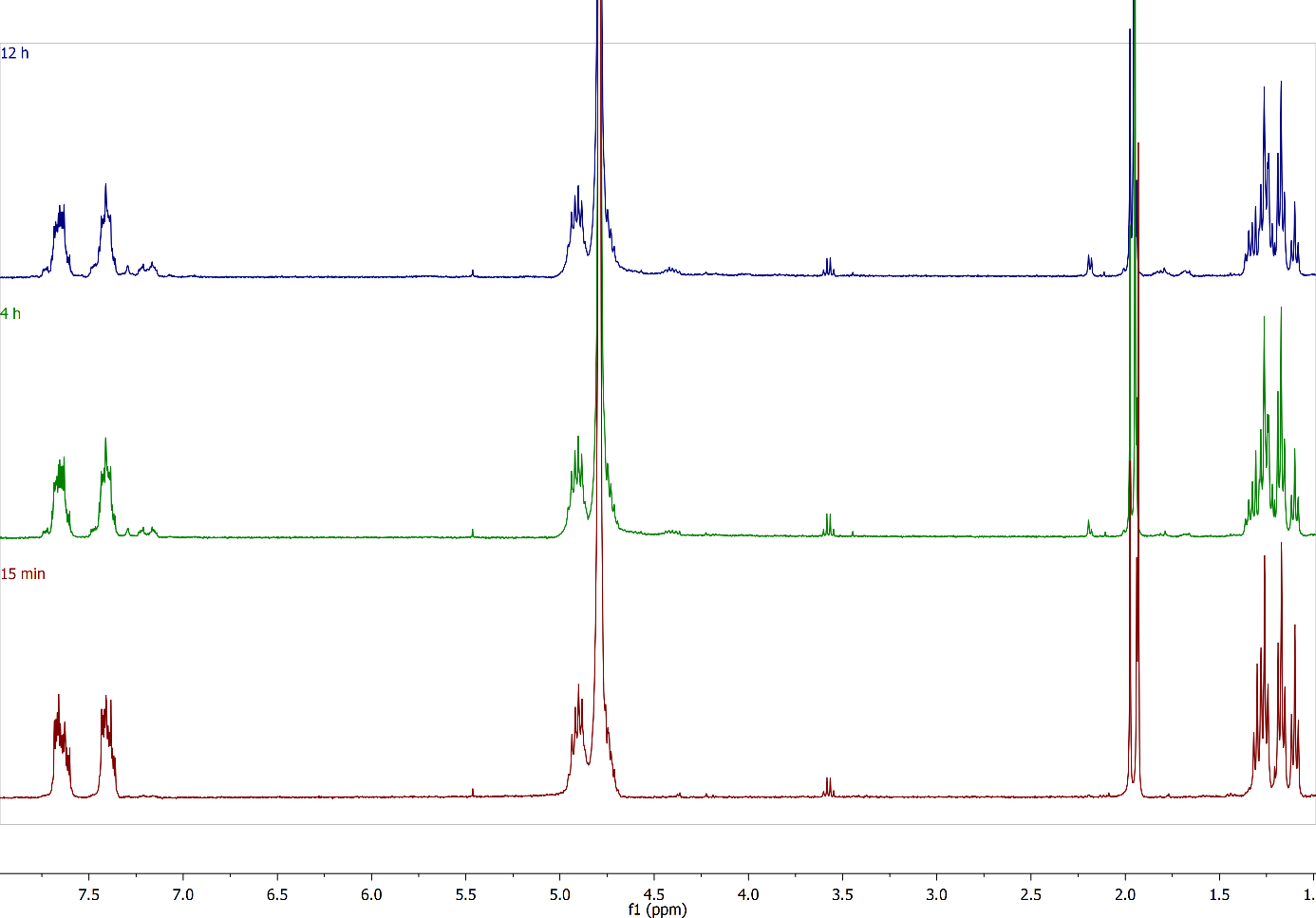


**Figure S16.** ^1^H NMR spectra of complex **2** in D_2_O measured after 15 min, 4 h, and 12 h of incubation at ambient temperature (from bottom to top).


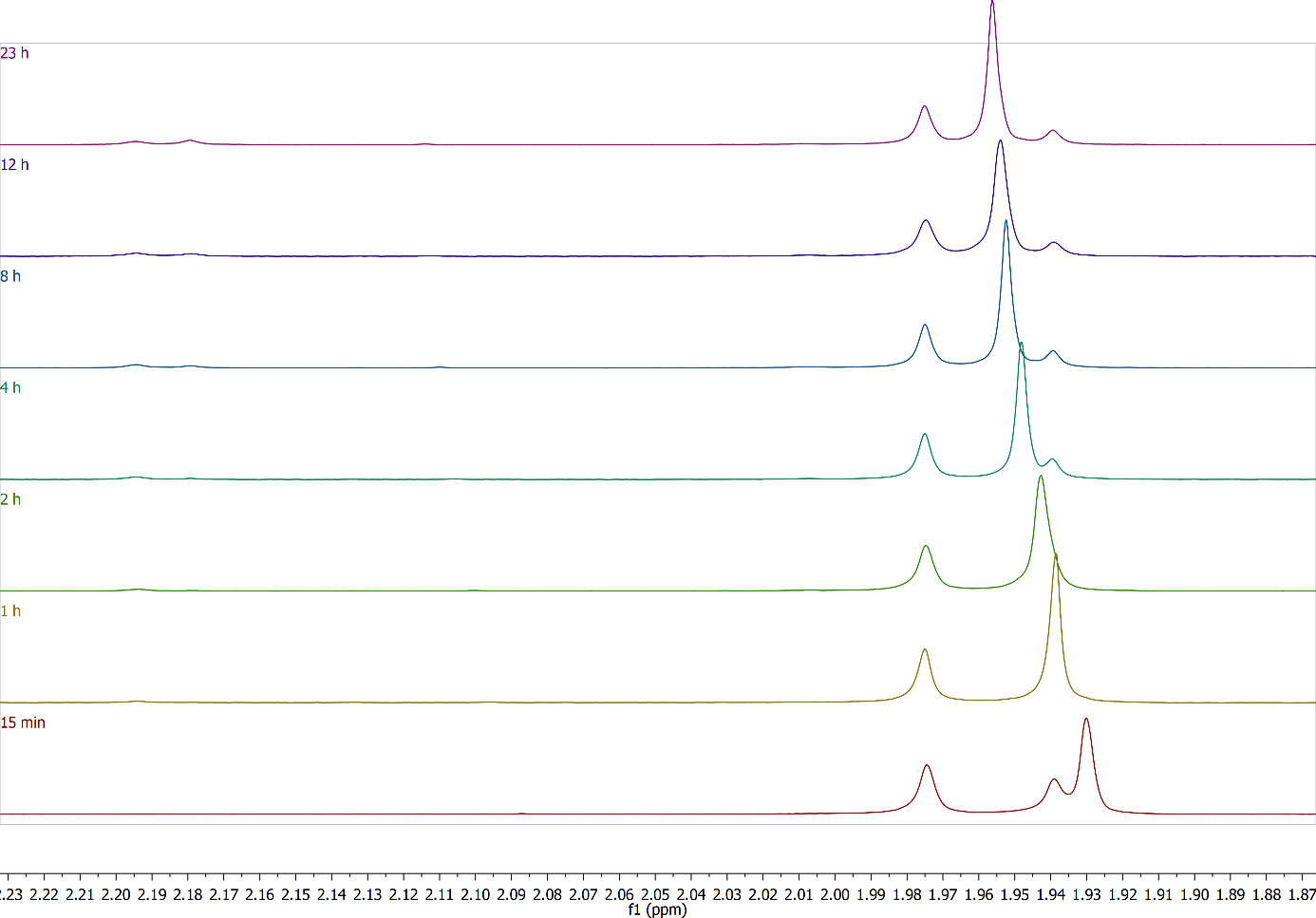


**Figure S17.** Time-dependent change of the appearance of the acetate signals in the ^1^H NMR spectra of complex **2** in D_2_O.

# RP-HPLC experiments

HPLC measurements were conducted on a Shimadzu Prominence system with a KNAUER Eurospher 100-5 C18 column (250 × 4 mm) at 25 °C, UV–Vis detection, and a MeOH/Milli-Q water mobile phase (0.1% FA) using a 35–98% MeOH gradient. The addition of 0.1% FA suppressed aquation, affording reasonably sharp chromatographic peaks; however, acetate (or dichloroacetate)–to–formate exchange likely occurred during elution, and data should therefore be interpreted with caution.


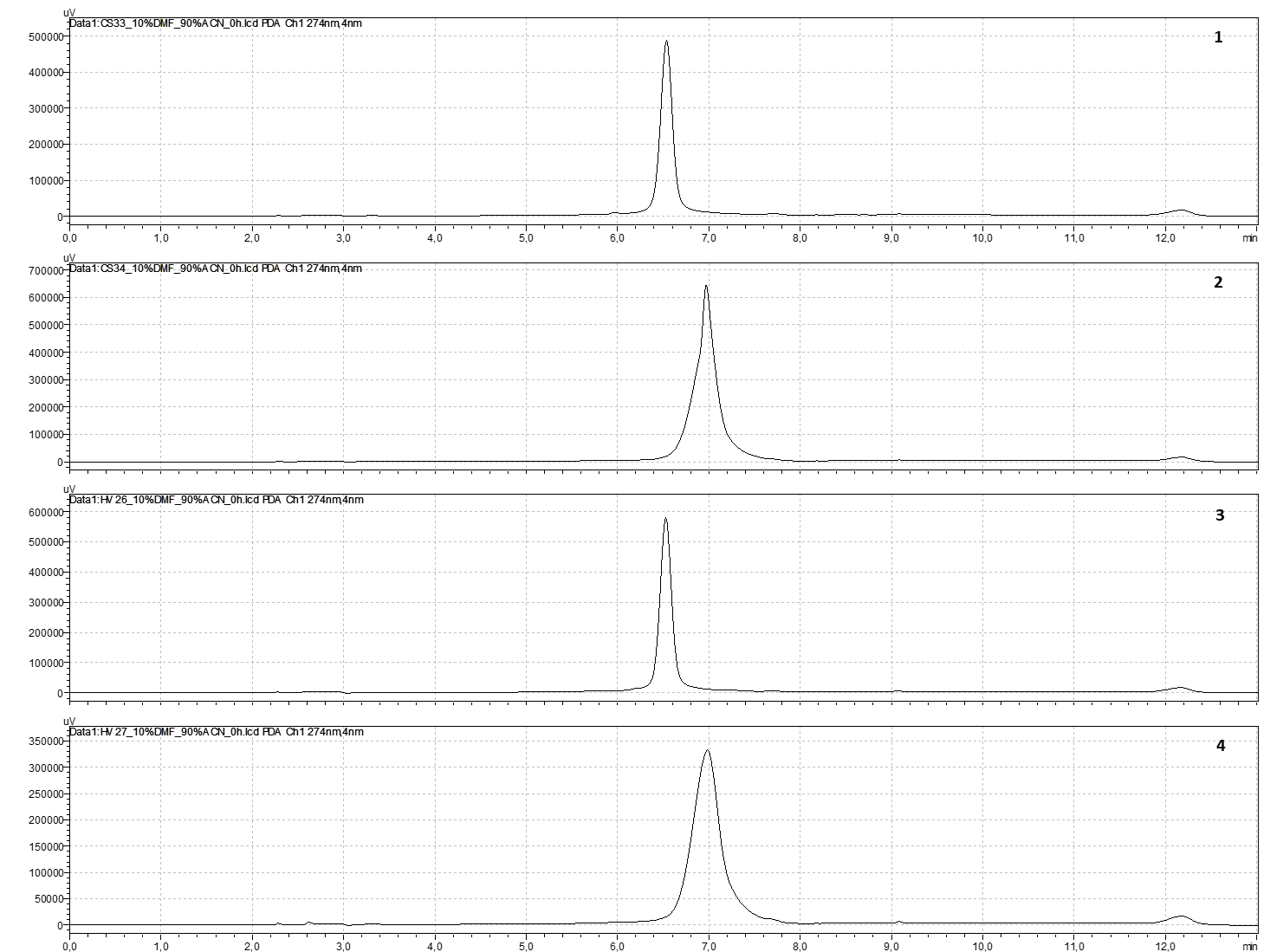


**Figure S18.** RP-HPLC chromatograms of complex **1-4** (top to bottom) dissolved in 1:9 (v/v) DMF/ACN mixture. Overlay of chromatograms recorded at 274 nm is shown.


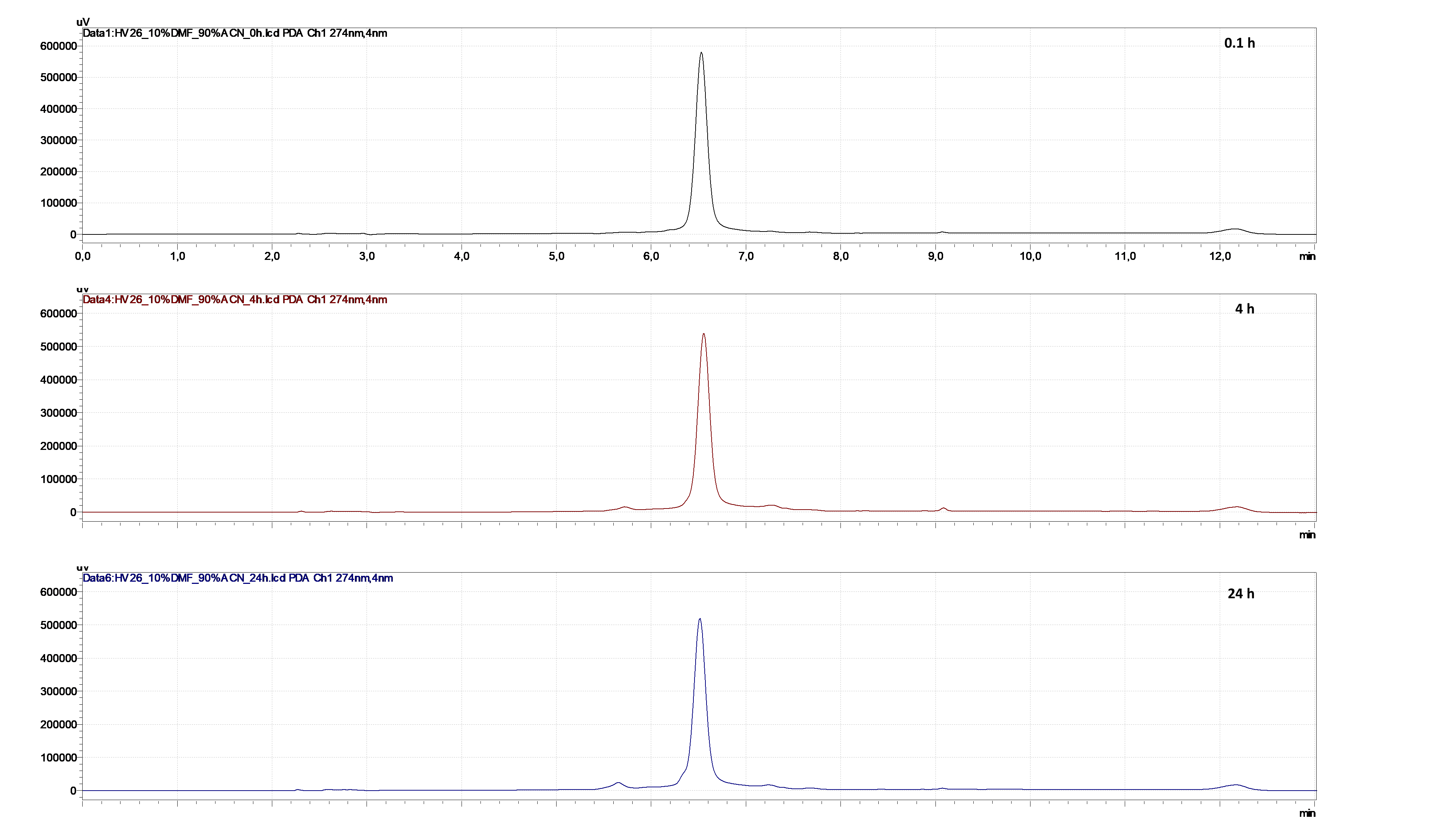


**Figure S19.** RP-HPLC chromatograms of complex **3** dissolved in 1:9 (v/v) DMF/ACN mixture after 0.1, 4 and 24 h (from bottom to top) of incubation at rt. Overlay of chromatograms recorded at 274 nm is shown.


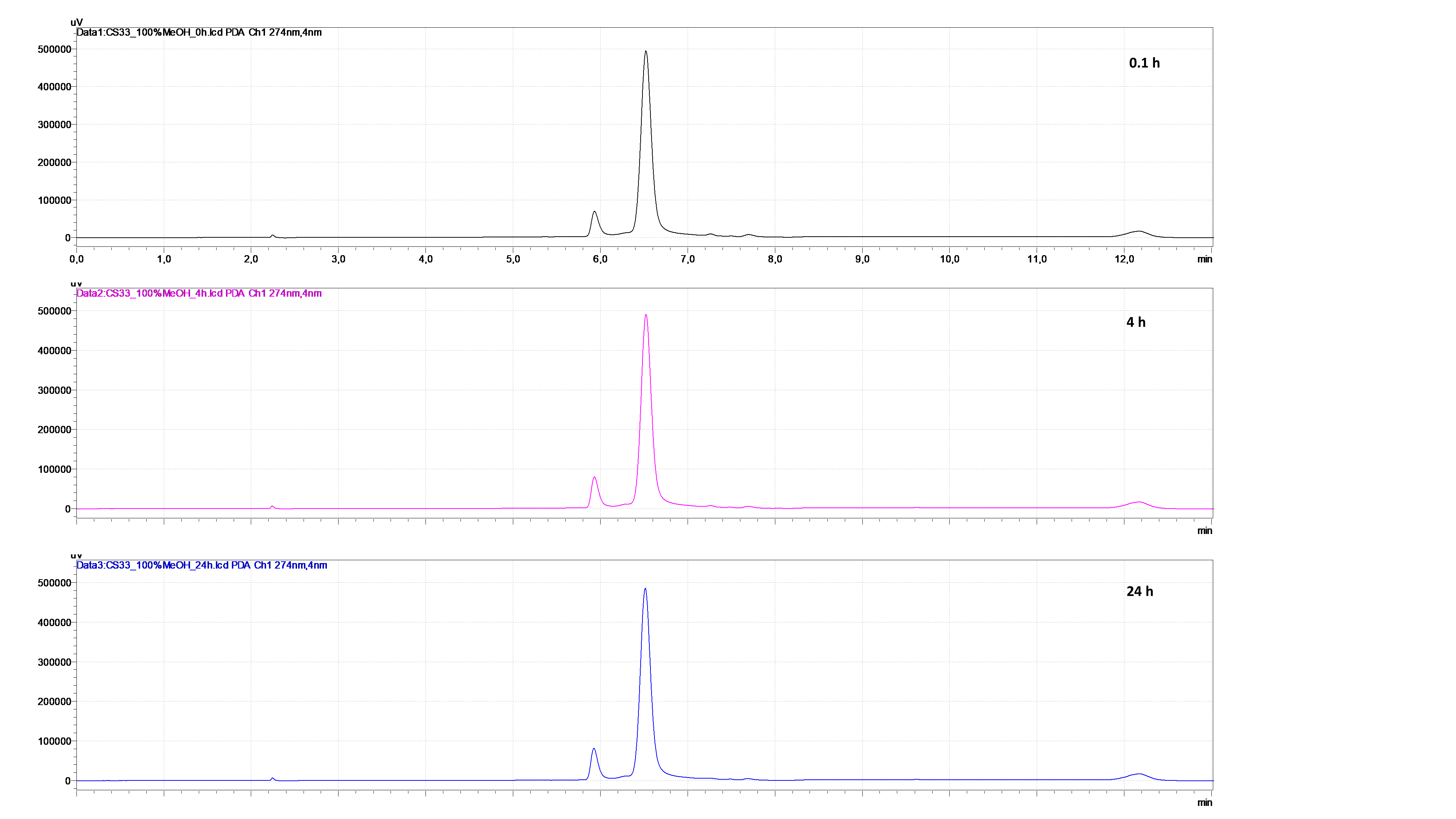


**Figure S20.** RP-HPLC chromatograms of complex **1** dissolved in MeOH after 0.1, 4 and 24 h (from bottom to top) of incubation at rt. Overlay of chromatograms recorded at 274 nm is shown.

# HPLC-MS stability of **1** and **2** in RPMI 1640


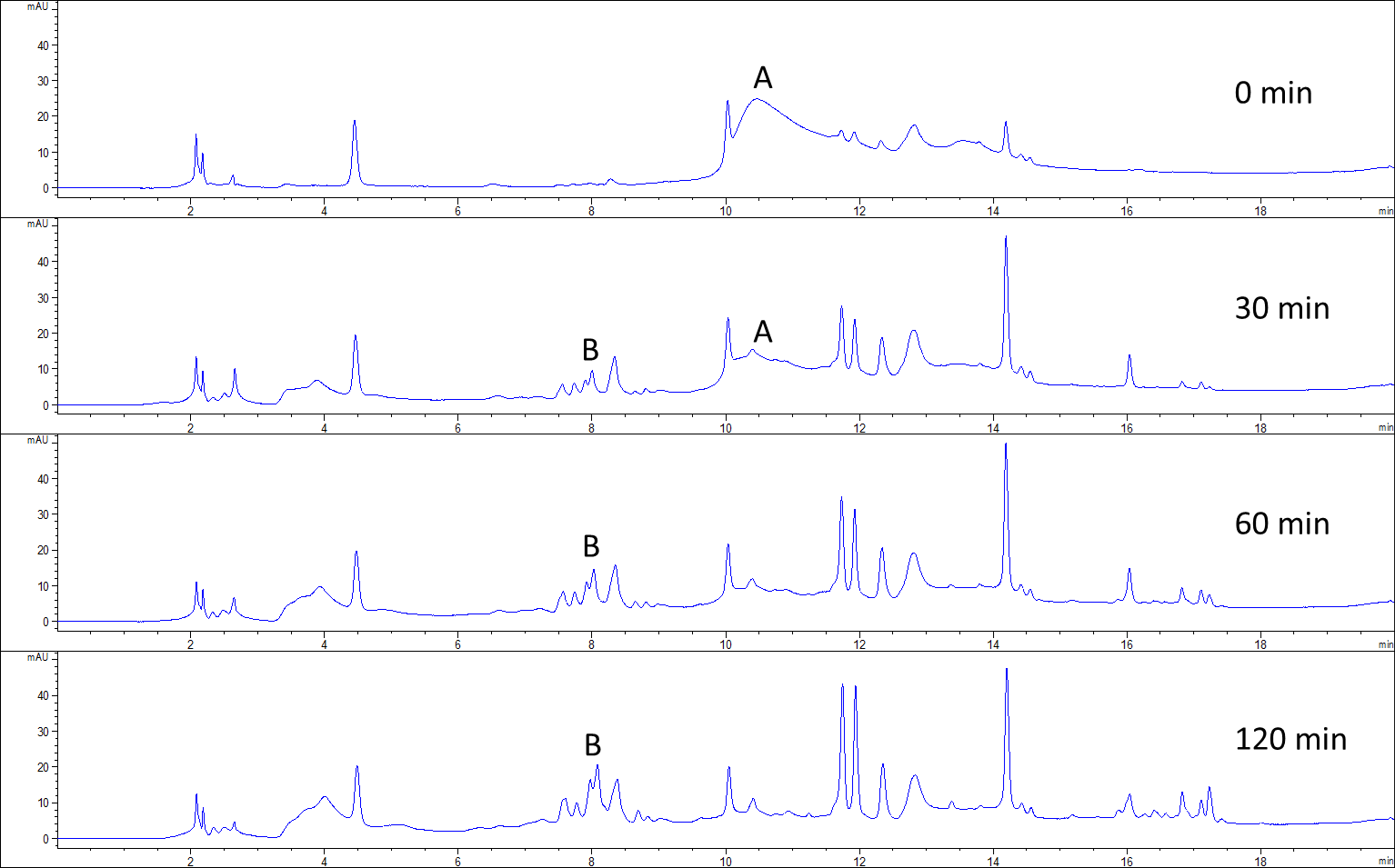


**Figure S21.** Interactions of complex **1** with components of RPMI 1640 (w/o FCS), monitored by RP-HPLC-MS. Chromatograms recorded at 280 nm are shown for the indicated time points. **A**: *m*/*z* 446 ± 0.5 (assigned to [**1**–2CH₃COO^-^–H⁺]⁺) and **B**: both *m*/*z* 577.2 ± 0.5 assigned to the L-leucine/isoleucine adduct.


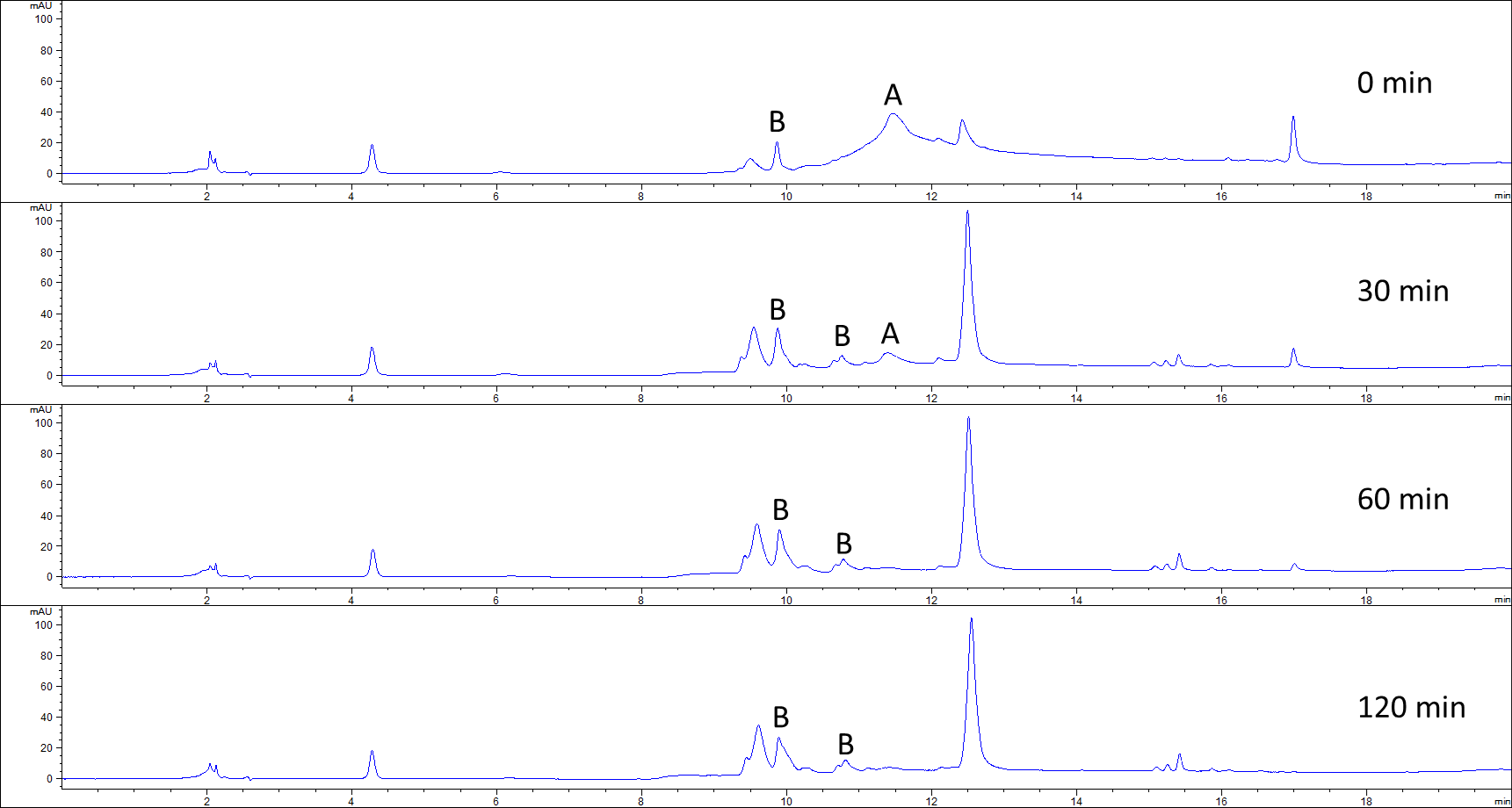


**Figure S22.** Interactions of complex **2** with components of RPMI 1640 (w/o FCS), monitored by RP-HPLC-MS. Chromatograms recorded at 280 nm are shown for the indicated time points. **A**: *m/z* 602 ± 0.5 (assigned to [**2**–CH₃COO^-^]⁺) and **B**: both *m/z* 673.3 ± 0.5 assigned to the L-leucine/isoleucine adduct.

**Figure S23.** Time-dependent change in the relative peak area of the LC-MS peaks corresponding to complexes **1** and **2** (and/or their aquated forms) after dissolving in RPMI 1640 (w/o FCS) as determined by RP-HPLC-MS. Data was obtained by integration of the extracted ion chromatograms at *m*/*z* 446 ± 0.5 (assigned to [**1** – 2CH₃COO^-^ – H⁺]⁺) and 602 ± 0.5 (assigned to [**2**–CH₃COO^-^]⁺), respectively.

**
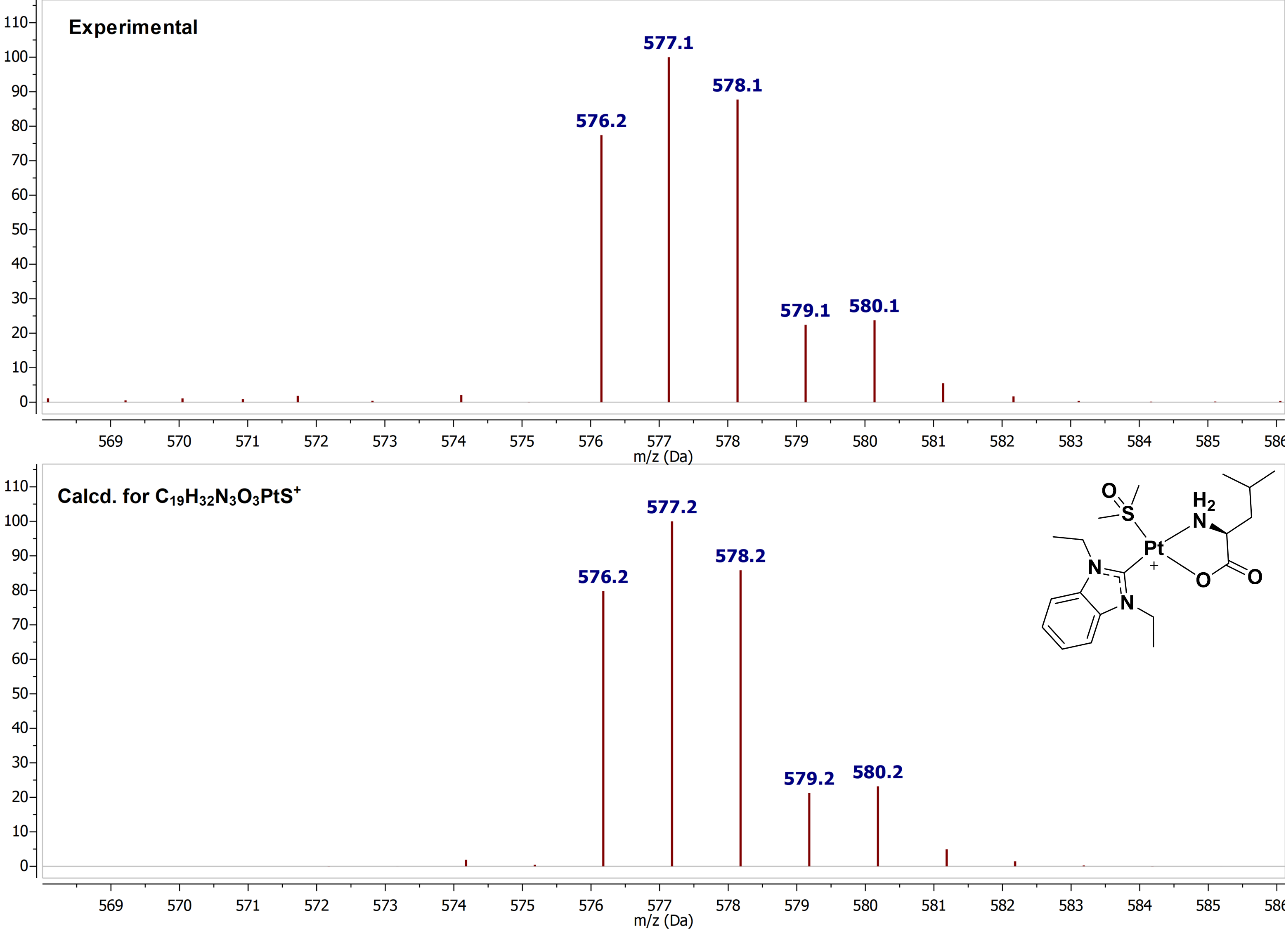
**

**Figure S24.** Interactions of complex **1** with the components of RPMI 1640 after 1 h of incubation at rt. A zoom of the acquired ESI-MS spectra of the HPLC peak with t_R_ = 8.3 min corresponding to adduct with the amino acid L-leucine or L-isoleucine. Experimental spectrum (top) vs. calculated (bottom) isotopic pattern is shown.


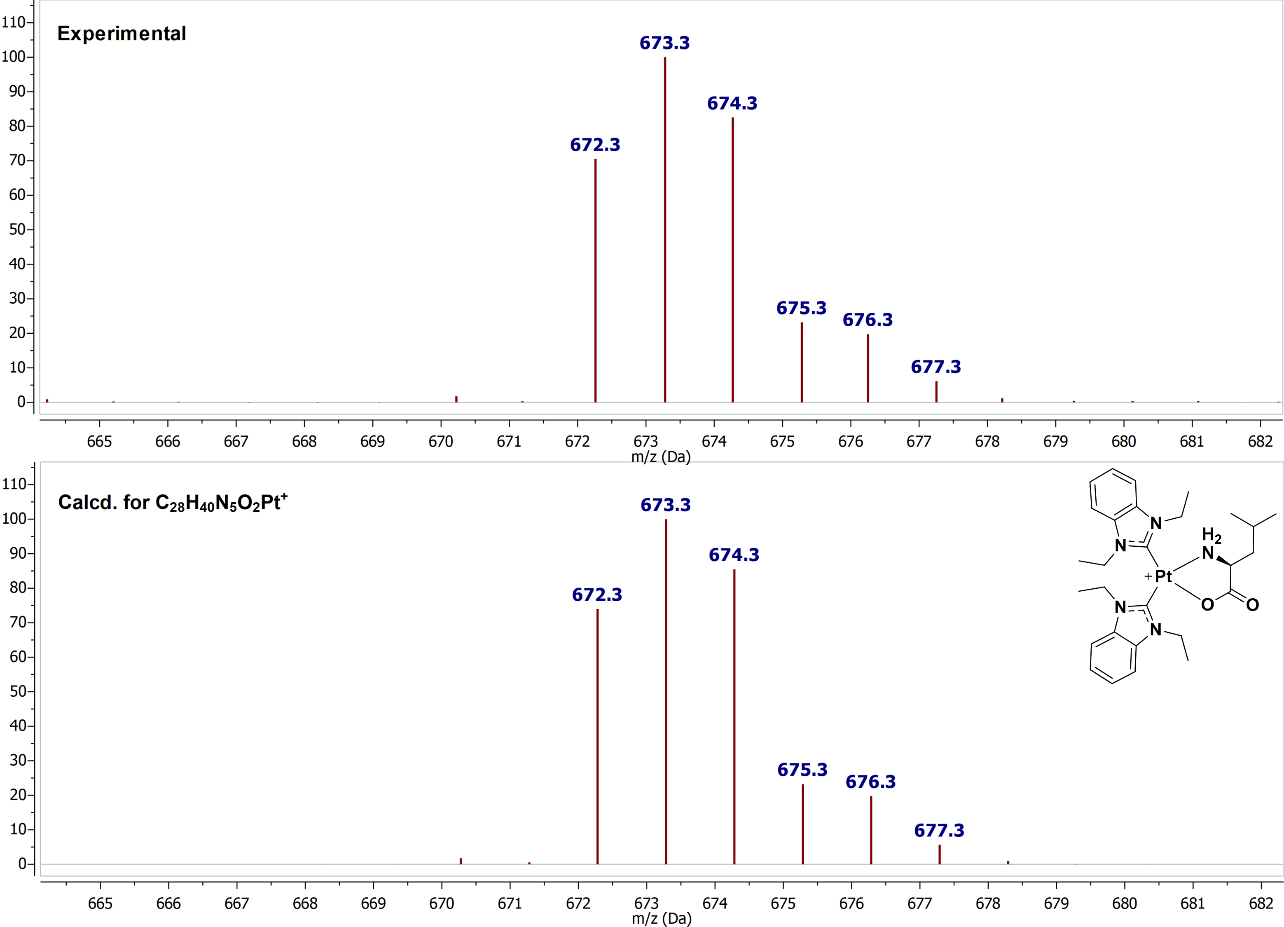


**Figure S25.** Interactions of complex **2** with the components of RPMI 1640 after 24 h of incubation at rt. A zoom of the acquired ESI-MS spectra of the HPLC peak with t_R_ = 10.9 min corresponding to adduct with the amino acid L-leucine or L-isoleucine. Experimental spectrum (top) vs. calculated isotopic pattern (bottom) is shown.

# Additional biological data

## Comparison of cytotoxicity of complexes **1**-**4** and carboplatin across the tested cell lines

**Figure S26.** Metabolic activity of non-cancerous HS-5 cells vs. A2780wt and A2780cis cells after treatment with complexes **1**-**4** and carboplatin at 50 µM for 72 h, determined in an MTT assay. ***: highly significant (p < 0.001); **: very significant (p < 0.01); *: significant (p < 0.05); ns: no significant difference. Values were calculated as the mean ± SEM of four independent experiments.

## Concentration-effect curves of complexes **1**-**4** and carboplatin on A2780wt and A2780cis cells

**Figure S27.** Concentration-effect curves of complexes **1** and **2** (left) and **3**, **4** and carboplatin (right) on A2780wt cells. Values represent mean ± SEM of four independent experiments.

**Figure S28.** Concentration-effect curves of complexes **1** and **2** (left) and **3**, **4** and carboplatin (right) on A2780cis cells. Values represent mean ± SEM of four independent experiments.

## Additional image of comet assay


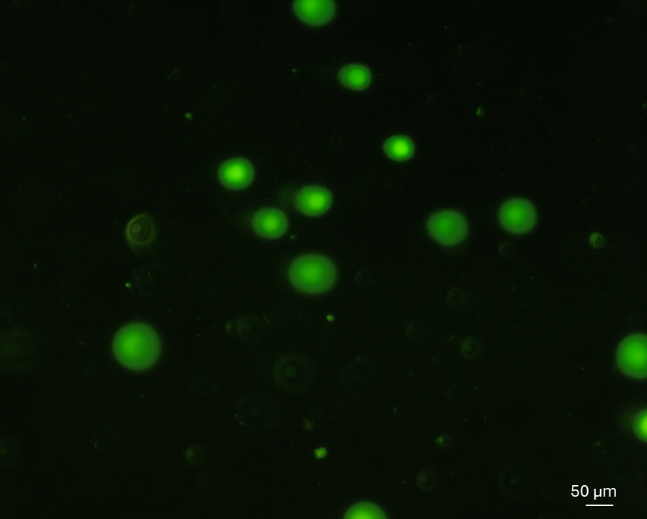


**Figure S29**. Fluorescence microscopic imaging as part of the comet assay using A2780wt cells after 48 h of incubation with carboplatin at a concentration of 50 µM.

## Induction of apoptosis and necrosis

**Table S5**. Induction of apoptosis and necrosis in A2780wt cells after incubation with complexes **1**-**4** in their free and loaded forms for 24 h at 25 µM and carboplatin as a reference at 50 µM. Values represent mean ± SEM of three independent experiments.

|  | Compound | w/o BGs | *EcN* | *Ec NM522* |
| --- | --- | --- | --- | --- |
| early apoptosis | **1** | 15.9 ± 1.55 | 8.70 ± 1.05 | 9.83 ± 0.40 |
|  | **2** | 9.37 ± 0.31 | 7.70 ± 1.15 | 8.83 ± 1.36 |
|  | **3** | 15.3 ± 1.17 | 10.2 ± 1.54 | 11.3 ± 0.77 |
|  | **4** | 13.2 ± 0.22 | 5.93 ± 0.35 | 6.53 ± 0.78 |
|  | carboplatin | 7.90 ± 0.29 | - | - |
| late apoptosis/ necrosis | **1** | 29.1 ± 2.56 | 23.9 ± 3.55 | 27.6 ± 4.68 |
|  | **2** | 25.9 ± 5.70 | 25.5 ± 4.59 | 22.6 ± 4.19 |
|  | **3** | 28.8 ± 2.50 | 31.8 ± 6.98 | 23.9 ± 3.92 |
|  | **4** | 46.9 ± 3.01 | 23.5 ± 4.41 | 26.0 ± 2.71 |
|  | carboplatin | 16.8 ± 0.69 | - | - |

**Table S6.** Induction of apoptosis and necrosis in A2780cis cells after incubation with complexes **1**-**4** in their free and loaded forms for 24 h at 25 µM and carboplatin as a reference at 50 µM. Values represent mean ± SEM of three independent experiments.

|  | Compound | w/o BGs | *EcN* | *Ec NM522* |
| --- | --- | --- | --- | --- |
| early apoptosis | **1** | 21.3 ± 1.18 | 8.53 ± 1.92 | 8.90 ± 2.06 |
|  | **2** | 6.37 ± 1.17 | 6.27 ± 1.59 | 6.47 ± 0.87 |
|  | **3** | 11.4 ± 1.82 | 6.17 ± 1.04 | 7.10 ± 1.93 |
|  | **4** | 22.9 ± 1.67 | 8.87 ± 1.34 | 6.03 ± 0.70 |
|  | carboplatin | 6.47 ± 1.75 | - | - |
| late apoptosis/ necrosis | **1** | 15.0 ± 1.65 | 14.2 ± 2.81 | 14.4 ± 2.83 |
|  | **2** | 10.0 ± 1.77 | 15.5 ± 1.49 | 15.1 ± 1.77 |
|  | **3** | 10.6 ± 0.95 | 13.6 ± 2.50 | 10.8 ± 1.14 |
|  | **4** | 18.1 ± 1.46 | 18.2 ± 2.86 | 14.3 ± 0.95 |
|  | carboplatin | 12.6 ± 1.17 | - | - |

## Caspase-3 induction

**Table S7.** x-fold caspase-3 induction compared to untreated cells on A2780wt cells after treatment with the free and loaded compounds. Values represent the mean ± SD of two independent experiments.

| Compound | w/o BGs | *E*. *coli* Nissle 1917 | *E*. *coli* NM522 |
| --- | --- | --- | --- |
| w/o compound | 1.00 ± 0.00 | 0.89 ± 0.14 | 1.31 ± 0.40 |
| **1** | 2.64 ± 0.32 | 3.20 ± 0.62 | 3.61 ± 0.31 |
| **2** | 0.39 ± 0.13 | 2.14 ± 0.61 | 2.34 ± 0.78 |
| **3** | 3.06 ± 1.20 | 2.34 ± 0.89 | 3.25 ± 1.19 |
| **4** | 1.89 ± 0.03 | 2.55 ± 0.86 | 3.14 ± 0.04 |

## Representative images of surface calreticulin exposure


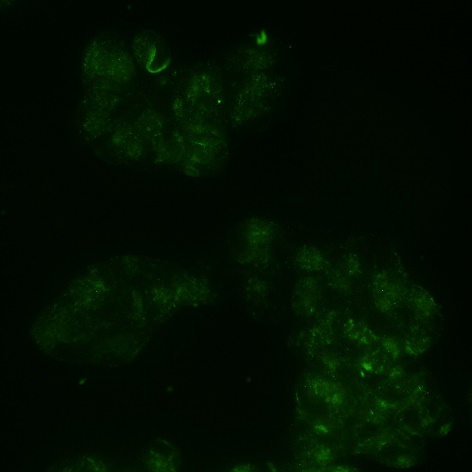

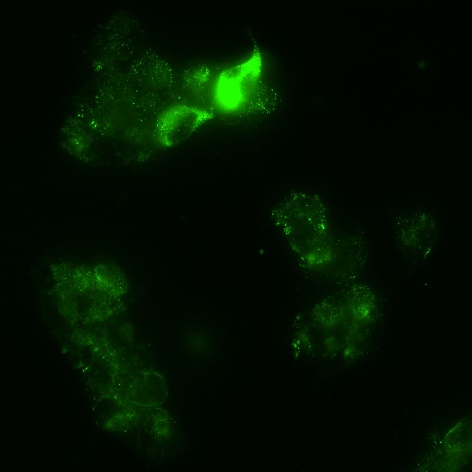

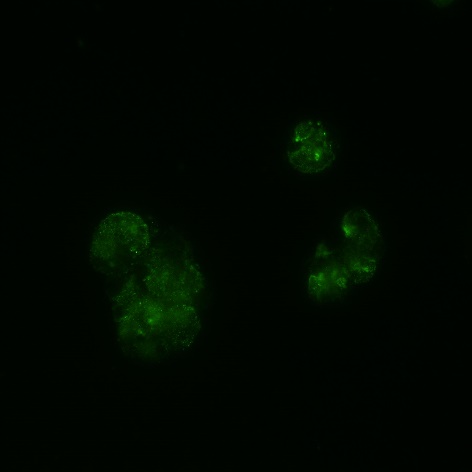

a b c


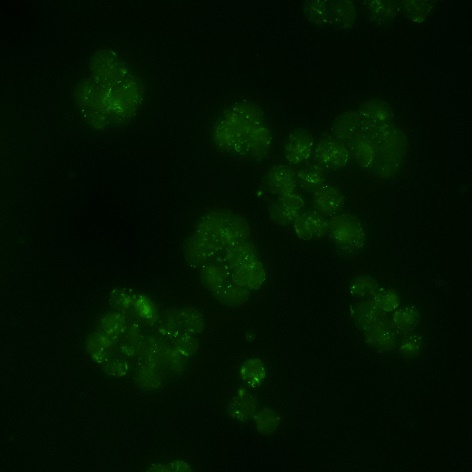

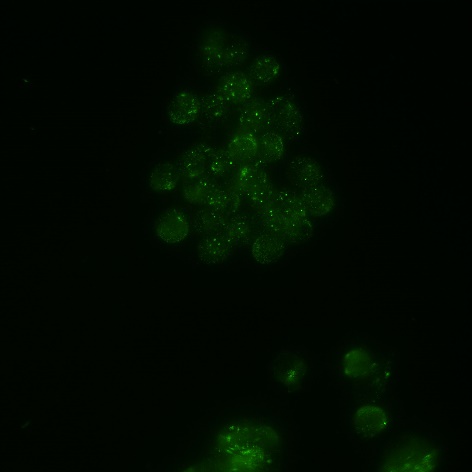

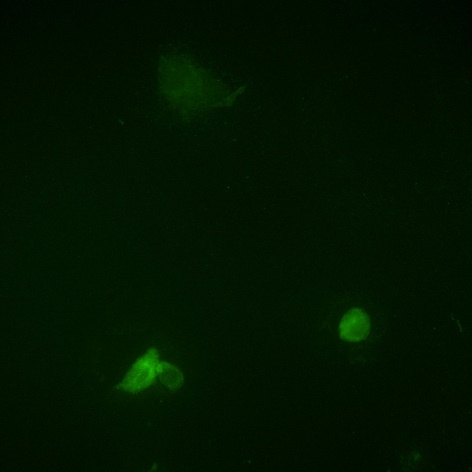

d e f

**Figure S30.** Fluorescence microscopic imaging showing an increase in the calreticulin concentration on A2780wt cells after treatment with unloaded **1** (a), **1** loaded in *EcN* (b), **1** loaded in *Ec NM522* (c), as well as an untreated control (d) and unloaded BGs (*EcN*, e; *Ec NM522*, f).

# Time-temperature program for the AAS measurement

**Table S8.** Graphite furnace program for the analysis of platinum (wavelength λ= 265.9450 nm).

| Step | Operation | Temperature | Heating rate | Holding time | Argon flow |
| --- | --- | --- | --- | --- | --- |
| 1 | Drying | 80 °C | 10 °C/s | 10 s | Maximum |
| 2 | Drying | 90 °C | 10 °C/s | 10 s | Maximum |
| 3 | Drying | 105 °C | 2 °C/s | 10 s | Maximum |
| 4 | Drying | 120 °C | 15 °C/s | 5 s | Maximum |
| 5 | Pyrolysis | 500 °C | 100 °C/s | 10 s | Maximum |
| 6 | Pyrolysis | 1000 °C | 100 °C/s | 10 s | Maximum |
| 7 | Pyrolysis | 1700 °C | 300 °C/s | 10 s | Maximum |
| 8 | Autozero | 1700 °C | 0 °C/s | 5 s | Stop |
| 9 | Atomization | 2400 °C | 1500 °C/s | 4 s | Stop |
| 10 | Cleaning | 2600 °C | 1000 °C/s | 5 s | Maximum |
